# Cancer-driver-agnostic targeting of amplified surface proteins identifies MPZL1 for selective CAR-T cell therapy

**DOI:** 10.1038/s41467-026-77465-5

**Published:** 2026-09-10

**Authors:** Sonia Jiménez-Vázquez, Anna Berthel, Christos Patsis, Mara Mitstorfer, Melanie Grimm, Luise Butthof, Lena Wendler-Link, Lio Böse, Hendrik Wiethoff, Ilse Hofmann, Kai Breuhahn, Thomas Longerich, Matthias M. Gaida, Peter Schirmacher, Judith Feucht, Meggy Suarez-Carmona, Patrick Schmidt, Fee Klupp, Martin Schneider, Markus Wallwiener, Dirk Jaeger, Niels Halama, Marco Breinig, Darjus F. Tschaharganeh

**Affiliations:** 1 Institute of Pathology, University Hospital Heidelberg, Heidelberg, Germany; 2 Helmholtz-University Group “Cell Plasticity and Epigenetic Remodeling”, German Cancer Research Center (DKFZ), Heidelberg, Germany; 3 Department of Tumor Immunology and Tumor Immunotherapy, Helmholtz Institute for Translational Oncology (HI-TRON) and German Cancer Research Center (DKFZ), Mainz, Germany; 4 Cluster of Excellence iFIT (EXC2180) “Image-Guided and Functionally Instructed Tumor Therapies”, University of Tübingen, Tuebingen, Germany; 5 Department of Pediatric Hematology, Oncology, Gastroenterology, Nephrology and Rheumatology, University Children’s Hospital Tuebingen, University of Tübingen, Tuebingen, Germany; 6 Antibody Core Facility, German Cancer Research Center (DKFZ), Heidelberg, Germany; 7 Institute of Pathology, University Medical Center Mainz, JGU-Mainz, Mainz, Germany; 8 TRON – Translational Oncology at the University Medical Center of the Johannes Gutenberg-University Mainz GmbH, Mainz, Germany; 9 Department of Medical Oncology, National Center for Tumor Diseases (NCT) and University Hospital Heidelberg, Heidelberg, Germany; 10 GMP and T cell Therapy, German Cancer Research Center (DKFZ), Heidelberg, Germany; 11 Department of Surgery, University Hospital Heidelberg, Heidelberg, Germany; 12 Department of Obstetrics and Gynecology, University Hospital Heidelberg, Heidelberg, Germany; 13 UCT and Department of Hematology, Medical Oncology and Pulmonology, University Medical Center Mainz, Mainz, Germany

**Keywords:** Cancer genomics, Targeted therapies, Cancer

## Abstract

Most cancer therapies target genetic alterations driving tumor growth, but many drivers are undruggable or rare, limiting their therapeutic reach. Here, we demonstrate that amplified genes encoding cell surface proteins provide a rich, cancer-driver–agnostic source of targets. We identify MPZL1 (myelin protein zero–like 1), amplified in up to 75% of patients with certain solid tumor types, as abundantly expressed on the surface tumor cells across diverse cancer types while being largely absent from healthy tissues, offering a favorable therapeutic window. We develop a monoclonal antibody targeting MPZL1’s extracellular domain and engineer chimeric antigen receptor (CAR)-T cells that selectively eliminate MPZL1-positive cancer cells in vitro. These CAR-T cells exhibit antitumor activity in human xenograft, autochthonous mouse, and patient-derived tissue explant preclinical models. This work establishes MPZL1 as a viable CAR-T cell target and provides a generalizable framework for exploiting amplified cell surface receptors as broadly applicable therapeutic targets.

## Abstract (web summary)

Current precision oncology strategies benefit only subsets of patients because cancer driving alterations are often infrequent or not readily tar-getable. Here, the authors identify MPZL1 as an amplified cell surface receptor broadly expressed across solid cancers and show that MPZL1-directed CAR T cells eliminate MPZL1 expressing tumors in multiple preclinical models.

## Introduction

Somatic genetic alterations are the underlying cause of cancer, and consequently they are widely used to define respective therapies for precision medicine. Most therapies focus on driver genes, which are fueling the progression of tumors and thus are attractive targets for cancer therapy^1,2^. In particular, point mutations in specific oncogenic driver genes, e.g., *KRAS* or *BRAF*, have been therapeutically exploited by developing corresponding inhibitors, which show great promise in patients harboring cancers with these mutations^3–6^. Moreover, driver genes, which are amplified by somatic copy number alterations (SCNAs) and thus exhibit increased dosage of their genetic material, are also targets of drug development efforts^7^. The most prominent gene in this regard is *HER2/ErbB2*, which is amplified in ~15% of breast cancer patients^8^, and can be targeted either by small molecule inhibitors or by monoclonal antibodies and antibody-drug conjugates^9,10^. However, despite progress in precision cancer therapy, approximately two-thirds of cancer patients lack actionable targets (e.g., due to druggability) and therefore are not amenable to precision oncology^11^.

More recently, substantial efforts have focused on circumventing the need to directly target oncogenic driver mutations. Many of these strategies exploit the concept of synthetic lethality (SL), in which cancer cells develop a dependency on specific genes or pathways as a consequence of their genetic context, even in the absence of a directly targetable driver event^12^. A well-established example of SL is the interaction between PARP and BRCA1/2, in which inhibition of PARP selectively impairs the survival of BRCA-deficient cells while sparing BRCA-proficient cells^13,14^. Moreover, collateral lethality is a subtype of SL which specifically targets genes associated with passenger deletions^15^, and often involves paralog pairs such as ENO1-ENO2^16^, ME2-ME3^17^ or MFRN1-MRFN2^18^. Although these findings are promising, their clinical translation remains constrained by the difficulty of developing selective inhibitors for many emerging targets.

Whereas targeting of ‘bystander’ alterations in large genomic deletions is already widely accepted, approaches harnessing collateral vulnerabilities created by genomic amplifications have only emerged recently^19,20^. Notably, large genomic amplifications not only comprise driver genes but also lead to dosage elevation of many other genes^7^, which could potentially be exploited for novel therapeutic strategies. Consequently, we hypothesized that, regardless of their cancer-driving potential, cell surface proteins (CSPs) encoded within amplified genomic regions could serve as selective entry points for therapies targeting cancer cells. Amplification of these genes, and associated elevated protein expression in cancer cells, could create a therapeutic window that enables selective targeting of tumor cells while minimizing effects on normal tissues, thereby revealing previously overlooked opportunities for precision medicine.

Here, we show that MPZL1, a cell surface receptor frequently amplified in solid cancers, represents a cancer-driver-agnostic therapeutic target amenable to CAR-T cell therapy. MPZL1-specific CAR-T cells exhibit antitumor activity in MPZL1-positive cancer cells in vitro, in xenograft, autochthonous mouse, and patient-derived tissue explant models.

## Results

### An integrative in silico approach identifies MPZL1 as a potentially targetable amplified CSP

**Fig. 1:**
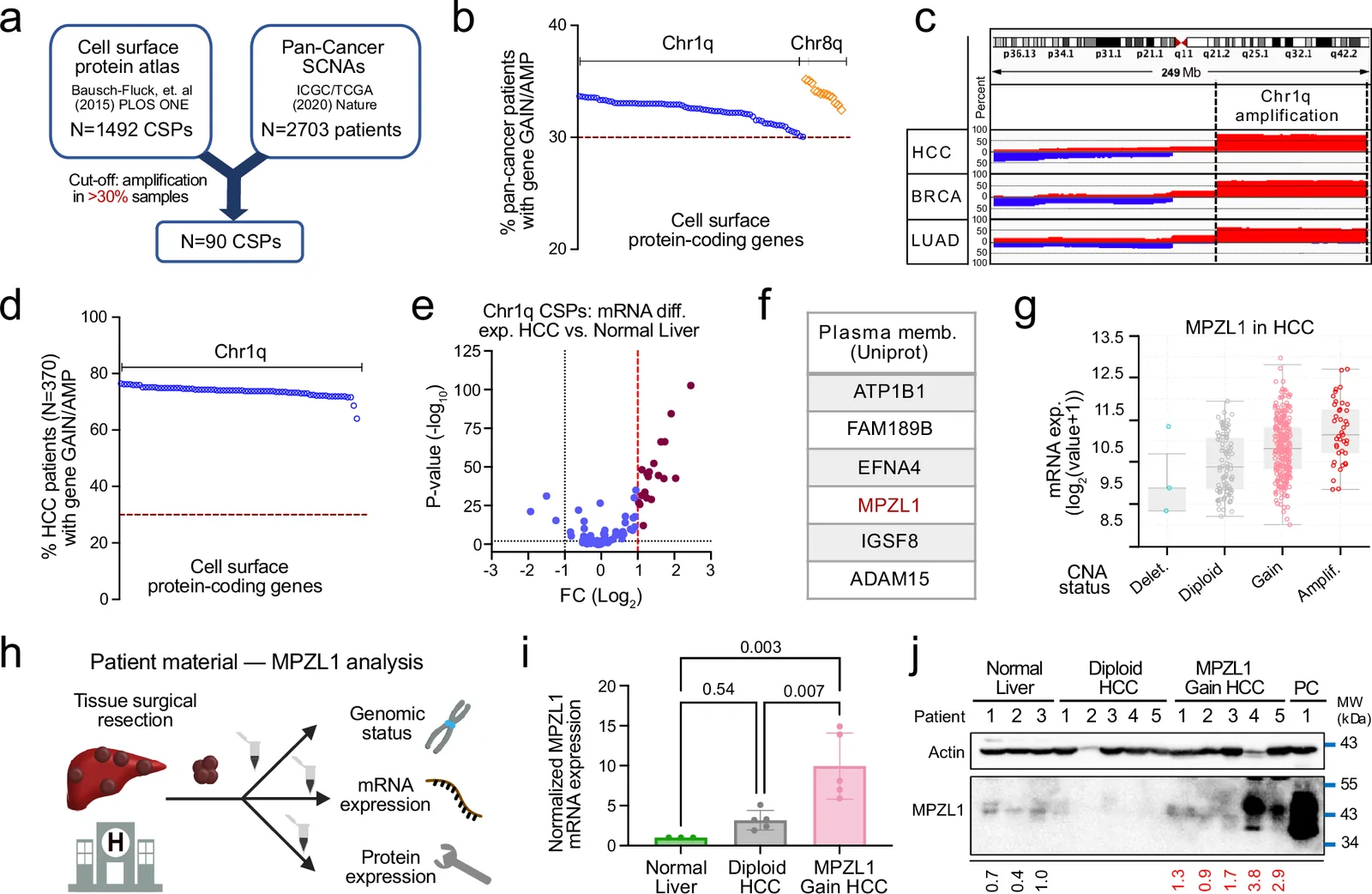
Identification of MPZL1 as a targetable amplified cell-surface protein. **a** Schematic of the in silico screening strategy. Copy number variation (CNV) status of 1492 cell-surface protein (CSP)-encoding genes was analyzed across a pan-cancer cohort (*n* = 2703). CNVs were classified using GISTIC2.0 as shallow deletion (−1), diploid (0), gain (1), or amplification (2). Candidate hits were defined as genes with gain or amplification in >30% of samples (*n* = 90). **b** Chromosomal distribution of hits, highlighting chromosome 1q as a recurrent hotspot (*n* = 77 genes). **c** Frequency of chromosome 1q somatic copy number alterations (SCNAs) in hepatocellular carcinoma (HCC, *n* = 370), breast invasive carcinoma (BRCA, *n* = 1080), and lung adenocarcinoma (LUAD, *n* = 516). Red indicates amplification (log2(CN/2) > 0.1) and blue indicates deletion (log2(CN/2) < 0.1). **d** Frequency of copy number gains and amplifications among hits in the HCC cohort (*n* = 370). **e** Differential mRNA expression of chromosome 1q-amplified CSP genes in liver tumors (*n* = 369) compared with normal liver tissue (*n* = 160). Data were analyzed using GEPIA2 and are expressed as log2 fold change (log2FC) with two-sided ANOVA *p* values; cutoff log2FC > 1 (*n* = 19 genes). **f** Six candidate CSPs annotated as plasma membrane-localized proteins based on UniProt. **g** Association between MPZL1 CNV status and mRNA expression in HCC (*n* = 360). Box plots show medians, interquartile range, and whiskers extending to 1.5× Interquartile Range. **h** Workflow to analyze MPZL1 expression for human liver samples. **i** MPZL1 mRNA expression (total *n* = 13, with *n* = 3, 5, and 5 per group). Dot plots show individual data points; bars indicate mean ± SD. Statistical differences among groups were assessed using one-way ANOVA followed by Tukey’s multiple-comparisons test (two-sided test). Adjusted *p* values are indicated. **j** MPZL1 protein expression was assessed by immunoblotting with actin as a loading control. (total *n* = 13 samples with *n* = 3, 5, and 5 per group, as in **i**). PC (positive control) indicates HLE cells overexpressing human MPZL1 (Supplementary Fig. 3). Quantification of MPZL1 protein expression was based on samples derived from gels/blots that were processed in parallel. Source data and uncropped blots are provided as a Source Data file.

To explore large chromosomal amplifications as a reservoir of cell surface targets, we leveraged the mass spectrometry–derived Cell Surface Protein Atlas, an open-access catalog of 1492 human membrane-bound proteins^21^, and analyzed copy number alterations of the corresponding genes across 2703 ICGC/TCGA pan-cancer samples^22^ (Fig. 1a–g, Supplementary Fig. 1). This analysis identified 90 CSP-coding genes (Supplementary Table S1 and S2), as amplified in up to 30% of tumors, with a strong clustering on chromosome 1q (*n* = 77), 20 of which were likewise identified to localize to the plasma membrane in the Human Protein Atlas (HPA)^23,24^. Notably, the chromosome 1q region emerged as a hotspot for amplified CSP-coding genes, amplified in >70% of hepatocellular carcinoma (HCC), breast invasive carcinoma, and lung adenocarcinoma cases across TCGA datasets^25–28^. Integrating differential expression analysis (GEPIA2^29^) with additional protein localization data (UniProt^30,31^) revealed that 19 chromosome 1q CSP genes (Supplementary Table S3) were upregulated in tumors versus normal tissues in HCC, of which 6 localize preferentially to the plasma membrane (Fig. 1e, f; Supplementary Table S4). Although these 6 candidates have been associated with cancer-related processes, none are established oncogenic drivers^32–37^, suggesting them as potential cancer driver-agnostic therapeutic targets.

We selected the cell surface receptor MPZL1 (myelin protein zero–like 1) as a putative candidate for further interrogation, as publicly available DepMap datasets^38^ indicate that MPZL1 knockout does not markedly impair cell growth across hundreds of tumor cell lines (Supplementary Fig. 2a–f), arguing against its classification as a classical cancer driver. Further, MPZL1 overexpression failed to cooperate with MYC overexpression in driving tumorigenesis in an autochthonous HCC mouse model, in contrast to constitutive active Notch1 (NICD) or mutant CTNNB1, two established HCC cancer-drivers which triggered rapid tumor formation in this genetic context (Supplementary Fig. 2g). While additional work is required to definitively establish a causal relationship between MPZL1 expression and metastatic potential, as has previously been suggested^35,39,40^, MPZL1-directed therapy may accordingly target tumor cells with elevated metastatic capacity lending further support for focusing on this cell surface receptor.

As genomic amplifications do not inevitably lead to higher mRNA or protein expression, we investigated the expression change of MPZL1 in relation to its genomic status. First, when correlating the *MPZL1* CNV status with its mRNA expression levels, we observed a progressive mRNA elevation from diploid to amplified cancers across different tumor types (Fig. 1g, Supplementary Fig. 1c, f). Next, to solidify observations based on publicly available data, we investigated MPZL1 in a patient cohort for which we had access to the genomic status, mRNA expression, and protein levels. Specimens were classified into “normal liver”, “diploid HCC”, or “MPZL1 gain HCC” via comparative genomic hybridization (CGH). Subsequent mRNA microarray and Western blot analyses revealed a marked increase in *MPZL1* mRNA expression, as well as MPZL1 protein expression, in gained HCCs compared to normal liver and diploid HCCs (Fig. 1h–j). Thus, these data show that genomic amplifications in MPZL1 not only increase its transcriptional levels but also protein levels, further suggesting MPZL1 as a suitable target for therapeutic strategies.

### MPZL1 is expressed at the plasma cell membrane in a variety of human solid cancers

**Fig. 2:**
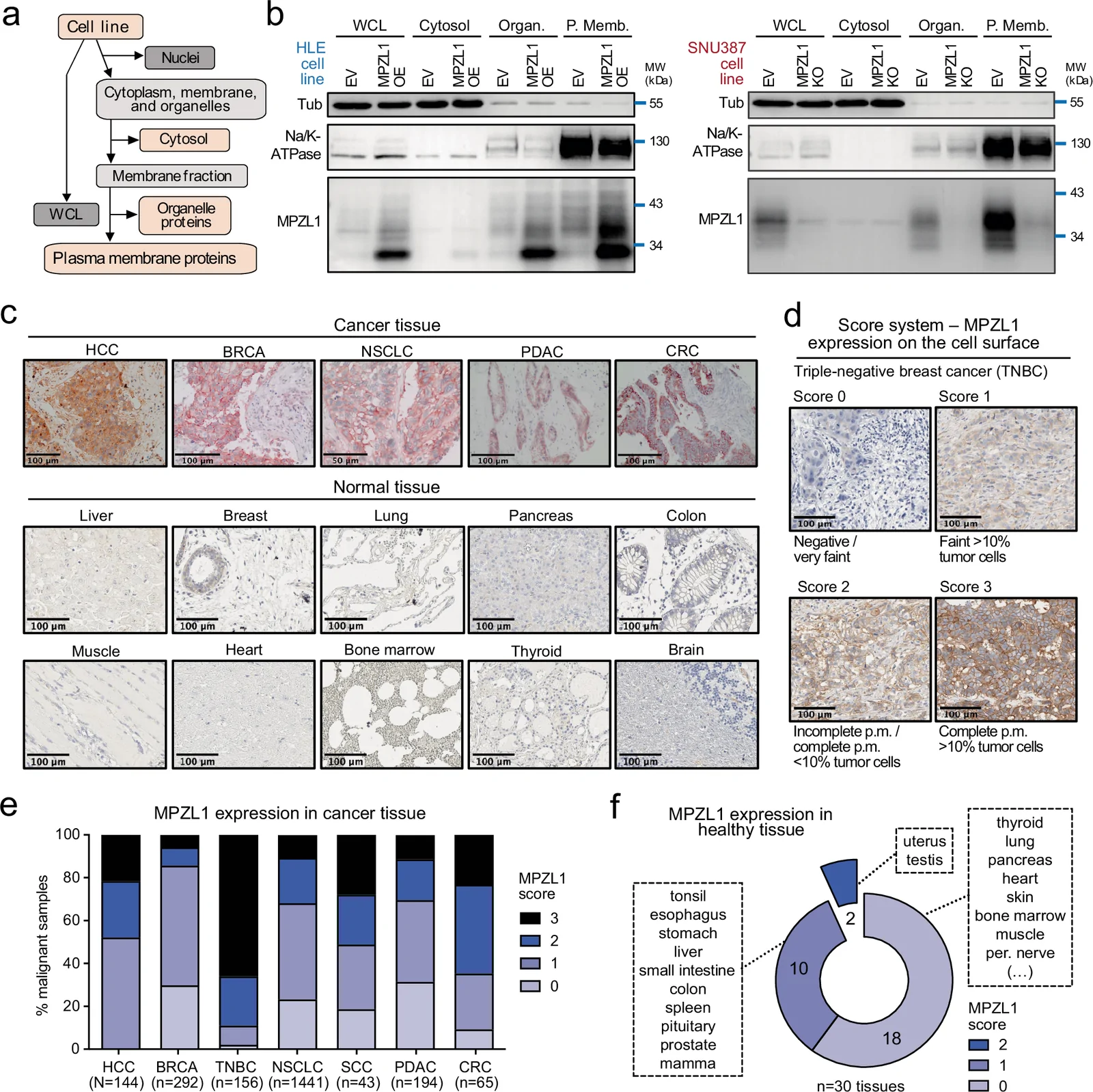
MPZL1 is expressed and localized at the plasma membrane in human solid cancers. **a** Schematic of the subcellular fractionation workflow used to analyze MPZL1 expression. **b** Immunoblot analysis of MPZL1 in fractionated lysates from isogenic liver cancer cell lines. HLE cells (low endogenous MPZL1 expression) were transduced with MPZL1 (overexpression, OE) or empty vector (EV), and SNU387 cells (high endogenous MPZL1 expression) were edited using CRISPR/Cas9 (MPZL1 knockout, KO). Fractions include whole-cell lysate (WCL), cytosolic, organelle, and plasma membrane compartments. Tubulin and Na⁺/K⁺-ATPase were used as cytosolic and plasma membrane markers, respectively. Immunoblots are representative of two independent experiments with similar results. **c** Representative immunohistochemistry (IHC) images of MPZL1 in tumor and normal tissues from tissue microarrays (TMAs). Scale bars, 100 μm. **d** Scoring system for plasma membrane-associated MPZL1 expression (scores 0–3) with representative triple-negative breast cancer (TNBC) samples. Scale bar, 100 μm. **e**, **f** Distribution of MPZL1 scoring in tumor (**e**) and normal (**f**) tissues. Percentages of samples per category are shown in donut plots. HCC hepatocellular carcinoma, BRCA breast cancer, TNBC triple-negative breast cancer, NSCLC non-small cell lung cancer, SCC squamous cell carcinoma, PDAC pancreatic ductal adenocarcinoma, CRC colorectal cancer. Source data and uncropped blots are provided as a Source Data file.

Focusing on MPZL1, we next investigated its protein expression in cancer cells in greater detail. First, using established human cancer cell lines, along with CRISPR/Cas9-mediated MPZL1 knockout and MPZL1 overexpression, we validated the specificity of a commercially available MPZL1 antibody and confirmed localization of MPZL1 at the plasma membrane. Further analyses indicated MPZL1 glycosylation^41^ and suggested a relationship between gene amplification and MPZL1 protein expression in cancer cell lines (Fig. 2a, b, Supplementary Fig. 3). Notably, in line with publicly available DepMap data (Supplementary Fig. 2), perturbation of MPZL1 did not affect proliferation of the tested cell lines (Supplementary Fig. 3d, e).

Next, we queried the HPA, which reports immunohistochemistry (IHC) data from tissue microarrays of 144 normal individuals across 44 tissue types and 216 cancer patients across 20 cancer types^23^, which indicated predominantly cytoplasmic MPZL1 expression with a granular pattern in several tissues and suggested that high MPZL1 expression is associated with unfavorable prognosis in several tumor types (e.g., HCC; https://www.proteinatlas.org/ENSG00000197965-MPZL1).To provide a complementary perspective on MPZL1 protein expression, we directly assessed MPZL1 expression in human tissues by performing IHC on paraffin-embedded samples using a commercial antibody whose specificity we validated in MPZL1 knockout as well as forced MPZL1 overexpression cell lines (Supplementary Fig. 3). Our analyses encompassed 2335 resected cancers across six tumor entities and 30 normal tissues (Fig. 2c–f). As we observed strong cell surface MPZL1 expression in multiple tumor samples, we developed a scoring system for MPZL1 levels at the plasma membrane (Fig. 2d), analogous to the well-established HER2 scoring system used in breast cancer^42^. Taken together, our analysis revealed that MPZL1 is expressed at the plasma membrane in human tumors, with high expression restricted to specific cancer subgroups (e.g., HCC, CRC, TNBC), extending previous reports that demonstrate elevated MPZL1 transcript expression in tumors^40^. In contrast, normal tissues generally exhibited low expression as compared to tumor tissue, with the exception of the uterus and testis (Fig. 2e, f), further supporting MPZL1 as a promising target as limited expression in normal tissues as opposed to markedly elevated tumor-specific expression could provide a therapeutic window for exploitation.

### Generation of a specific antibody targeting extracellular domains of MPZL1-protein

**Fig. 3:**
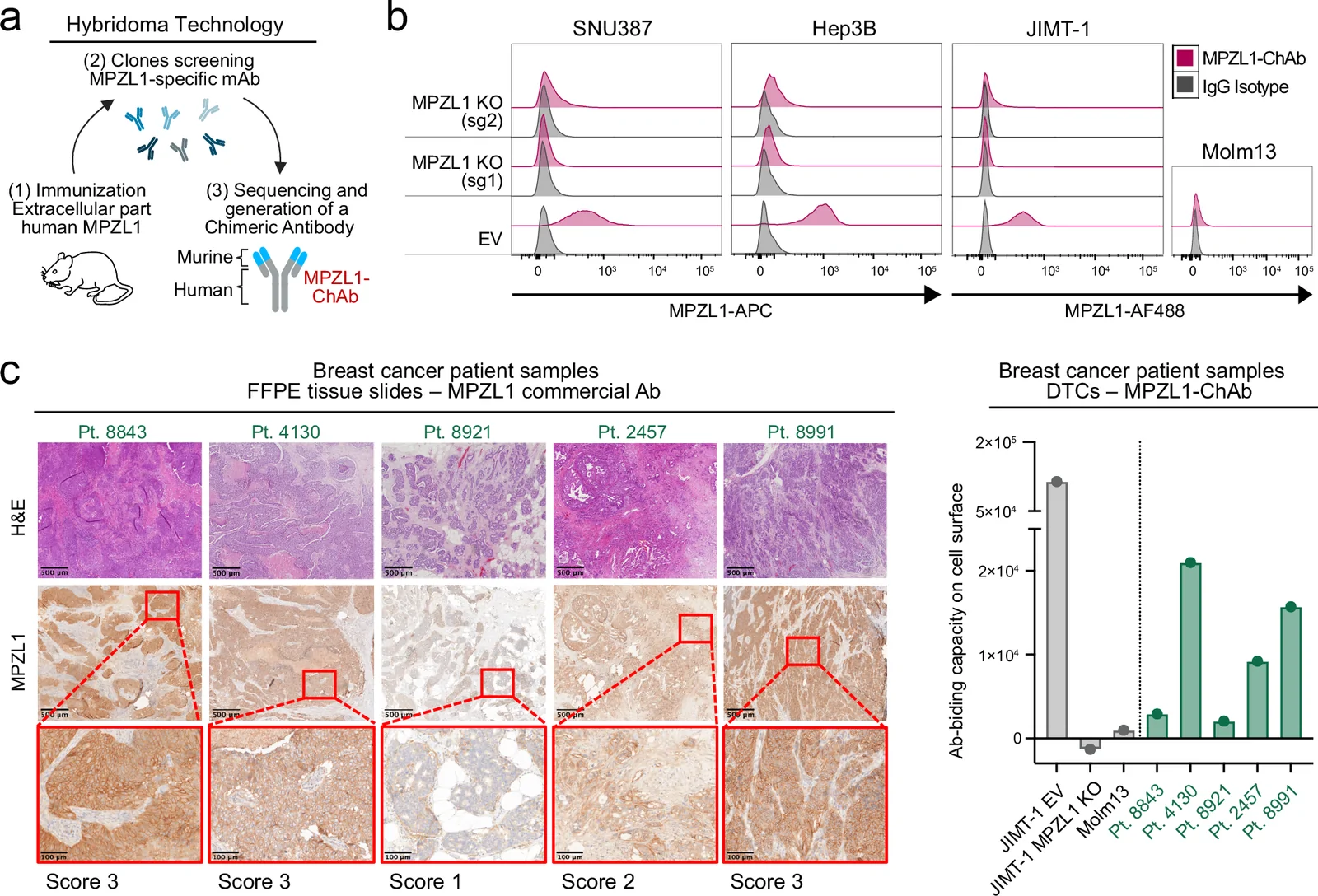
Generation and validation of a chimeric antibody targeting the extracellular domain of MPZL1. **a** Schematic of the antibody generation strategy. **b** Flow cytometry analysis showing binding of MPZL1-ChAb to three human cancer cell lines, including mock-transduced parental cell lines (EV, empty vector) and corresponding MPZL1 knockout (KO) cells generated using two independent CRISPR/Cas9 guides (sg1 and sg2). Molm13 cells lacking endogenous MPZL1 expression serves as a negative control. IgG Isotype serves as control for non-specific antibody binding. Data are representative of at least two independent experiments with similar results. **c** Validation of MPZL1-ChAb specificity in primary breast cancer specimens. Matched formalin-fixed paraffin-embedded (FFPE) tumor tissues and dissociated tumor cells (DTCs) from patients were analyzed (*n* = 5 independent patient samples). Left, representative H&E and MPZL1 IHC staining (two magnifications; Scale bars, 500 μm and 100 μm, respectively). Right, quantification of MPZL1-ChAb binding to DTCs by flow cytometry (*n* = 5 independent patient samples). JIMT-1 EV, JIMT-1 MPZL1-KO, and Molm13 cells were included as controls. Bar plots show individual values for each sample (*n* = 1). Source data are provided as a Source Data file.

Next, we aimed to unlock the therapeutic potential of elevated MPZL1 protein expression using antibodies that recognize its extracellular domain. The commercially available MPZL1-antibody that we used to profile MPZL1 on FFPE tissue (Fig. 2) binds to the cytoplasmatic domain of the human protein. Consequently, we generated a recombinant chimeric antibody (MPZL1-ChAb) that specifically targets the extracellular domain of MPZL1 (Fig. 3, see Methods). Testing the specificity and functionality of our MPZL1-ChAb in established cancer cell lines using FACS-based analysis to measure cell surface binding, along with CRISPR/Cas9-mediated MPZL1 knockout and MPZL1 overexpression, we observed that it solely detected human MPZL1 protein (Fig. 3b, Supplementary Fig. 4) and was unfortunately not suitable for IHC or Western blot. Our attempts to generate additional antibodies that recognize murine MPZL1 or are suitable for IHC failed. Nonetheless, we verified that the binding capacity^43^ of our MPZL1-ChAb to tumor cells reflected that of the commercially available MPZL1 antibody we used for IHC of human tumors (Fig. 3c), indicating that the MPZL1 commercial antibody can be used to screen patients potentially suitable for MPZL1-targeted therapies. Of note, treatment of established cancer cell lines with our MPZL1-ChAb did not affect cell survival (Supplementary Fig. 4), indicating that this antibody is unlikely to be useful for neutralizing MPZL1 function to kill cancer cells, consistent with its non-essential role in cancer cell growth (Supplementary Fig. 2). In summary, these results demonstrate the specificity of our MPZL1-ChAb towards cell surface-exposed human MPZL1 protein.

### Generation of MPZL1 CAR-T cells that show high efficiency and specificity in vitro

**Fig. 4:**
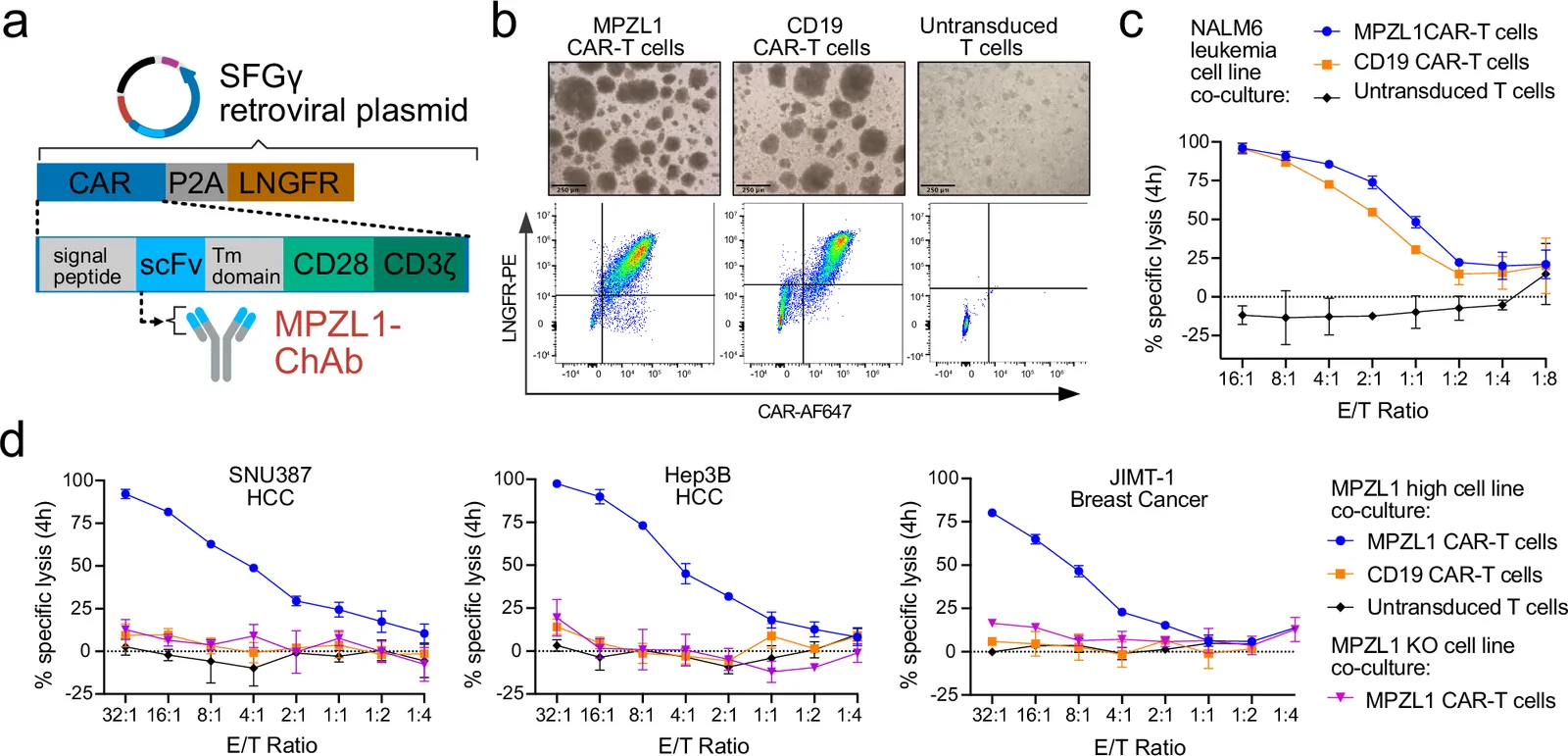
MPZL1 CAR-T cells mediate antigen-specific tumor cell killing in vitro. **a** Schematic of the MPZL1 CAR construct. **b** CAR-T cell characterization five days after transduction. Top, representative bright-field images of MPZL1 CAR-T, CD19 CAR-T, and untransduced T cells. Bottom: flow cytometry analysis of LNGFR⁺ CAR-T cells. Data are representative of at least three independent experiments with similar results. **c**, **d** T-cell cytotoxicity assays using luciferase-expressing target cells at decreasing effector-to-target ratios for 4 h. MPZL1 CAR T-cells are compared with CD19 CAR T-cells and untransduced T cells. RPMI medium and 0.2% Triton X-100 served as overall negative and positive controls, respectively. **c** Cytotoxicity against NALM6 cells (MPZL1^high^; CD19^high^). **d** Cytotoxicity against MPZL1^high^; CD19^low^ cancer cell lines (SNU387, Hep3B, JIMT-1) and matched MPZL1 knockout controls. Data show the mean ± SD of three technical replicates. Data shown are representative of at least two biological replicates per cell line with similar results. Source data are provided as a Source Data file.

In order to develop an MPZL1-specific therapeutic modality, we chose a CAR-T cell approach, providing the ability to directly target and eradicate cells that express the specific antigen for which they are designed on their surface^44–47^. As we were not able to generate antibodies that recognize murine MPZL1, we focused on targeting human MPZL1 and used the sequences of the variable heavy (V_H_) and *kappa* light (V_L_) chains of the MPZL1-ChAb. We built specific scFvs, which were introduced in a second-generation CAR composed of co-stimulatory CD28 and CD3ζ signaling domains linked to truncated LNGFR as a marker peptide. These components were incorporated in a SFGγ retroviral vector, which was used to generate MPZL1 CAR-T cells (Fig. 4a, Supplementary Fig. 5, see Methods). To functionally validate the specificity and killing capability of MPZL1 CAR-T cells, they were co-cultured with luciferase-expressing target cells, and cytotoxicity was assessed via bioluminescence-based assays. First, using NALM6 leukemia cells, which express both MPZL1 and CD19 antigens (Supplementary Fig. 6), we observed up to 100% lysis for both, CD19 as well as of MPZL1 CAR-T cell treatment (Fig. 4c). Next, we utilized MPZL1^high^ cell lines from different cancer types including HCC, breast, lung, pancreas and glioblastoma and found that MPZL1 CAR-T cells exhibited robust killing capacity against these cancer cell lines, whereas CD19 CAR-T cells or untransduced T cells did not (Supplementary Fig. 6). Thus, regardless of the origin of the cancer entity MPZL1 CAR-T cells can execute their antitumor function. In order to directly test the antigen-specific killing capacity of MPZL1 CAR-T cells, we used isogenic pairs of cancer cell lines with MPZL1 KO (Supplementary Fig. 3) in co-culture assays. MPZL1 CAR-T cells exhibited 80% to 100% lysis of all MPZL1^high^ target cell lines at the highest E/T ratios utilized (blue curve). In contrast, we did not observe specific killing of the respective isogenic MPZL1 KO cell line pairs (Fig. 4d). Alongside additional validation steps to comprehensively characterize CAR-T cells^48,49^ (Supplementary Figs. 5 and  6), these results demonstrate the ability and specificity of our MPZL1 CAR-T cells to rapidly eliminate MPZL1-expressing cancer cells in vitro.

### MPZL1 CAR-T cells are efficient and specific to target MPZL1-expressing human xenograft tumors and eradicate autochthonous MPZL1-expressing HCC tumors in vivo

**Fig. 5:**
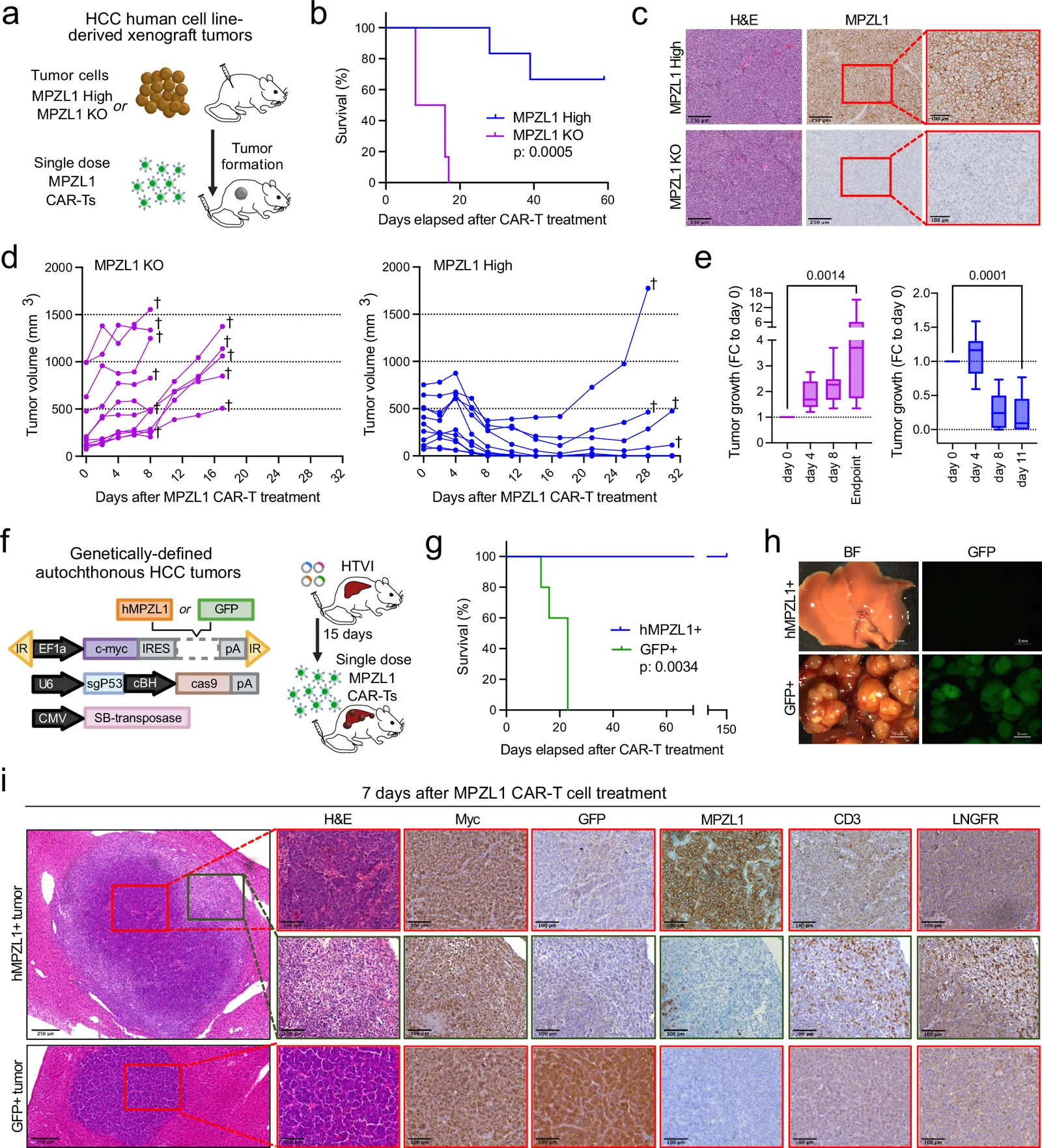
MPZL1 CAR-T cells mediate potent antitumor activity in HCC mouse models. **a**–**e** Xenograft HCC model. **a** Experimental design. NSG mice bearing subcutaneous Hep3B EV (MPZL1^high^) or MPZL1 KO tumors (~360 mm³) received a single intravenous dose of MPZL1 CAR-T cells (*n* = 6 per group). **b** Overall survival analysis. Statistical differences among groups (*n* = 6 per group) were assessed using a two-sided Log-rank (Mantel–Cox) testing. *p* value are indicated. **c** Representative H&E and MPZL1 IHC staining. Scale bars, 250 μm and 100 μm. **d** Tumor growth kinetics following treatment (day 0 = treatment). Individual tumor sizes are plotted with *n* = 11 independent tumors per group measured over time. Cross indicates termination of experiment. **e** Quantification of tumor growth expressed as fold change from baseline. Each graph represents a different group (left, MPZL1 KO; right, MPZL1^high^), with *n* = 11 independent tumors per group measured over time. Within each group, statistical differences among time points were assessed using one-way ANOVA followed by Tukey’s multiple-comparisons test (two-sided test). Adjusted *p* value are indicated. Box plots show the median, the 25th and 75th percentiles, and whiskers extending to the minimum and maximum values. **f**–**i** Autochthonous HCC model. **f** Experimental design. Liver tumors were induced by hydrodynamic delivery of c-MYC and p53 KO together with either MPZL1 or GFP (MycOE;p53⁻/^−^;MPZL1 or GFP; *n* = 5 per group). CAR-T cells were administered on day 15. **g** Overall survival analysis. Statistical differences among groups (*n* = 5 per group) were assessed using a two-sided Log-rank (Mantel–Cox) testing. *p* value are indicated.**h** Representative bright-field and GFP stereomicroscopy of livers. Scale bar, 5 mm. **i** Early tumor analysis 7 days after CAR-T treatment showing representative H&E and immunostaining for Myc, GFP, MPZL1, CD3 (T cell marker), and LNGFR (CAR-T cell marker). Source data are provided as a Source Data file.

Encouraged by our in vitro observations, we next tested the effect of MPZL1 CAR-T cells in vivo. First, we used a human HCC cell line-derived mouse xenograft model. HCC tumors were generated by subcutaneously injecting either Hep3B MPZL1^high^ cells or the respective isogenic Hep3B MPZL1 KO cells in the flanks of immunodeficient NSG (NOD.Cg-*Prkdc*^*scid*^*Il2rg*^*tm1Wjl*^/SzJ) mice^50^. When tumors reached an average volume of 360 mm^3^, a single dose of 1 × 10^7^ MPZL1 CAR-T cells was intravenously applied and tumor growth monitored over time (Fig. 5a). Treatment with MPZL1 CAR-T cells provided a significant survival advantage in mice harboring MPZL1^high^ tumors compared to those with *MPZL1* KO. While mice having Hep3B MPZL1 KO tumors did not respond to treatment, those with Hep3B MPZL1^high^ tumors rapidly experienced tumor regression. In relation to the time of CAR-T cell administration (day 0), Hep3B MPZL1 KO tumors continued growing until the animals were sacrificed due to their large tumor volumes. However, although Hep3B MPZL1^high^ tumors still increased in size by an average of 1.1-fold by day 4, this was followed by a 0.28-fold decrease already by day 8 (one-fourth of the initial volume, *p* value: <0.0001). Strikingly, 7 out of the 11 MPZL1^high^ tumors were completely eradicated without signs of relapse (follow-up until day 60), and only the remaining 4 tumors underwent regrowth. IHC staining confirmed the specific expression of MPZL1 on the cell surface of cancer cells within relapsed Hep3B MPZL1^high^ tumors, in contrast to Hep3B MPZL1 KO tumors where MPZL1 expression was absent (Fig. 5b–e). We performed a similar experiment with human breast cancer tumors by injecting either JIMT-1 MPZL1^high^ cells or the respective JIMT-1 MPZL1 KO cells in NSG mice. Again, MPZL1 CAR-T cell treatment provided a significant survival advantage to mice harboring MPZL1^high^ tumors compared to those with MPZL1 KO tumors (Supplementary Fig. 7a–d), indicating that a single dose of MPZL1 CAR-T cells sufficed to induce a specific therapeutic response in vivo.

**Fig. 6:**
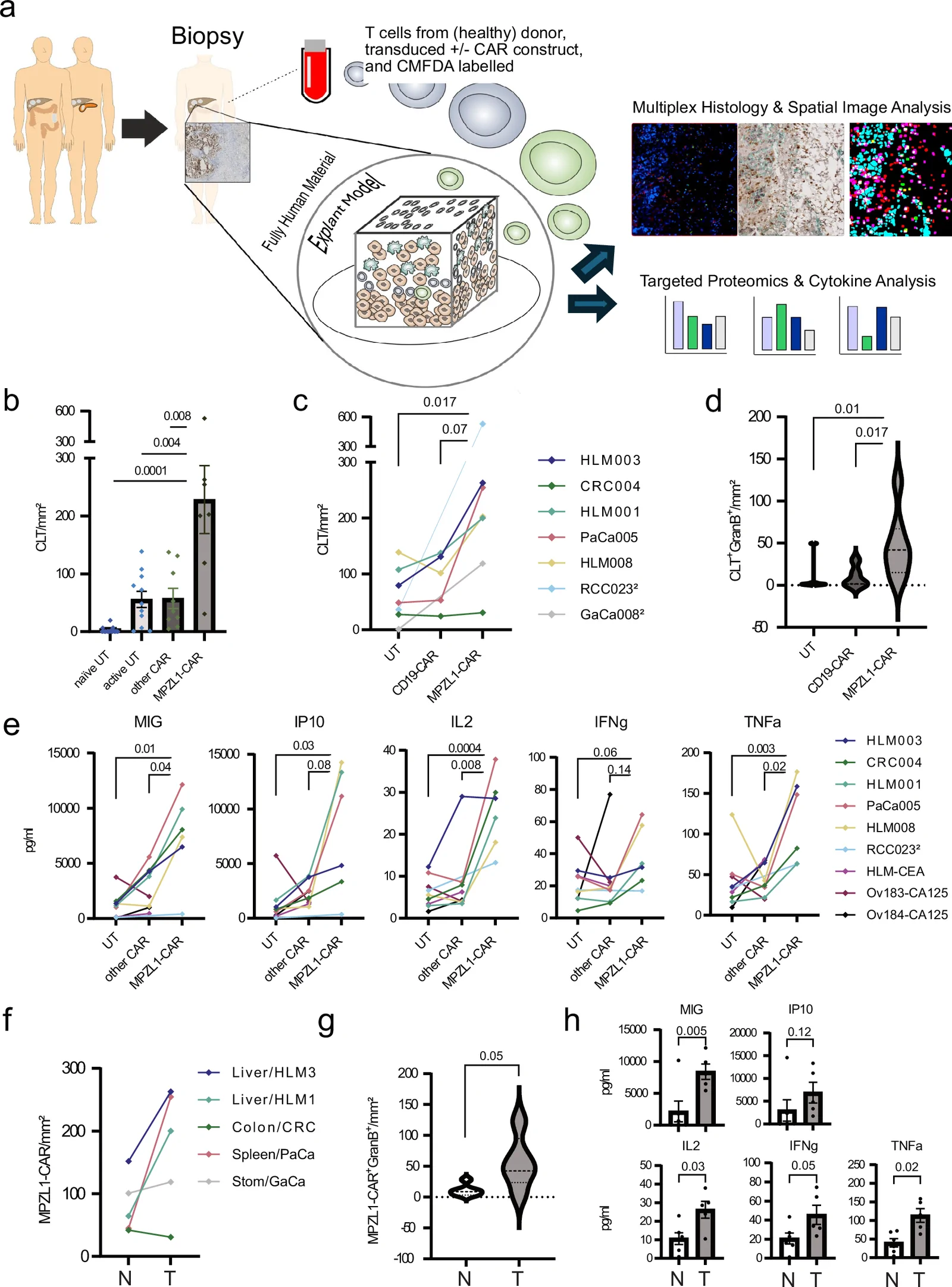
MPZL1 CAR-T cells demonstrate tumor-selective activation in human tissue explants. **a** Schematic of the human tissue explant and T cell tracing model. **b**–**e** Tumor explants from patients across six tumor types (1xCRC: Colorectal Cancer, 21xHLM: Colorectal Cancer Liver Metastases, 1xPaCa: Pancreatic Ductal Adenocarcinoma, 2xOv: Ovarian Cancer, 1xGaCa: Gastric Cancer and 1xRCC: Renal Cancer) were analyzed following treatment with CMFDA-labeled T cells (CLT). Antigen expression in human samples was confirmed for MPZL1, CA125, and CEA (Fig. S8). **b** Infiltration of MPZL1 CAR-CLT compared with naïve UT (*n* = 29) active UT (*n* = 11) and other CAR-CLT populations as indicated (CA125-CAR: *n* = 2, CEA-CAR: *n* = 2, CD19-CAR: *n* = 5). Mean cell numbers per mm² are shown. **c** Infiltration of MPZL1 CAR-CLT (*n* = 7), CD19 CAR-CLT (*n* = 5) and (active) UT-CLT (*n* = 7) in matched patient explants. **d** Quantification of granzyme B (GranB)-positive CLT. MPZL1 CAR-CLT (*n* = 7), CD19 CAR-CLT (*n* = 5) and (active) UT-CLT (n = 7). *N* = 7 samples (1xCRC, 3xHLM, 1xPaCa, 1xGaCa, 1xRCC). **e** Cytokine expression following treatment. MPZL1 CAR-CLT (*n* = 6), (active) UT-CLT (*n* = 9) or other CAR-CLT (CA125-CAR: *n* = 2, CEA-CAR: *n* = 1, CD19-CAR: *n* = 5). Samples as in (**b**). **f**–**h** Normal versus tumor tissue comparison. Normal (N) tissues (*n* = 6 patients, 3x Liver, 1xColon, 1xSpleen, 1xStom = Stomach) and matched tumor (T) explants (*n* = 5, 2x HLM, 1x CRC, 1x GaCa, 1x PaCa) were analyzed following treatment with CMFDA-labeled MPZL1 CAR-T cells. **f** Infiltration of MPZL1 CAR-CLT in normal (*n* = 5) and tumor (*n* = 5) tissue. **g** Quantification of GranB-positive CLT in normal (*n* = 6) and tumor (*n* = 5) tissue. **h** Cytokine expression following treatment in normal (*n* = 6) and tumor (*n* = 5) tissue. Colored lines relate to explants from the same patients. Data are shown as mean ± SEM unless otherwise indicated. Violin plots show median, 75th and 25th percentiles (middle, top and bottom line). Statistical differences were assessed using the Mann–Whitney *U* test, and two-tailed *p* values are indicated. ²UT-CLT and CD19-CAR were not tested, and comparison of MPZL1-CAR with the untreated control of the same patient is shown. Source data are provided as a Source Data file.

While human xenograft models provide valuable information to evaluate CAR-T cell killing efficiency, subcutaneously implanted tumor cells do not resemble physiological tumor formation and potential barriers imposed by tissue complexity. To overcome this shortcoming, we generated an autochthonous liver tumor model to test the impact of MPZL1 CAR-T cells on liver tumors (Fig. 6f–i). Using hydrodynamic injection of plasmids encoding c-MYC coupled either to MPZL1 cDNA (MycOE;p53−/−;MPZL1) or GFP (MycOE;p53−/−;GFP) in conjunction with CRISPR/Cas9 mediated knockout of p53, we produced liver tumors either with or without MPZL1 expression originating from hepatocytes in their actual liver tissue context. To ensure that forced expression of MPZL1 per se had no discernible effect on liver tumor formation, we first generated a cohort of MycOE;p53−/−;GFP mice and MycOE;p53−/−;MPZL1 mice without CAR-T cell treatment and found that tumor onset and survival of both groups did not show significant differences. IHC staining of resulting MycOE;p53−/−;MPZL1 tumors revealed strong membranous expression of human MPZL1 protein on the cell surface of the tumor cells, whereas no signal was detected in MycOE;p53−/−;GFP tumors (Supplementary Fig. 7e, f). These results further suggest that elevated MPZL1 expression is not a bona fide cancer driver in the context of our HCC model. Further, detailed necropsy revealed no pulmonary metastases in both the MycOE;p53−/−;MPZL1 and MycOE;p53−/−;GFP cohorts, arguing against a critical role for MPZL1 in metastatic progression in this genotypically defined mouse model (data not shown). Next, we used this liver tumor model and administered a single intravenous dose of 1 × 10^7^ MPZL1 CAR-T cells (directly after primary in vitro expansion) 15 days after HTVI. While MycOE;p53−/−;GFP mice rapidly developed large tumors and had to be sacrificed by day 23 after treatment, MycOE;p53−/−;MPZL1 mice did not show any signs of tumor development and survived until the experimental endpoint (day 100) (Fig. 5g). Indeed, MycOE;p53−/−;GFP mice showed multiple GFP-positive tumors, while no tumor nodules were found in MycOE;p53−/−;MPZL1mice at the end of the experiment (Fig. 5h).

Finally, to better understand the early effects of MPZL1 CAR-T treatment on tumors, we analyzed tumor-bearing livers shortly after therapy. Therefore, tumors were induced via HTVI as described above (Fig. 5f), mice received a single dose of 1 × 10⁷ MPZL1 CAR-T, and livers were harvested 7 days after treatment. Microscopic examination of MycOE;p53−/−;GFP tumors revealed multiple tumor nodules with no significant immune cell infiltration. However, most of the detected MycOE;p53−/−;MPZL1 tumor nodules displayed a ring of immune cells surrounding the tumor and invading its inner core, while other hMPZL1+ tumor nodules were completely infiltrated by immune cells, with no histologically detectable tumor cells. Further immunohistochemical analyses of tumor sections revealed that MycOE;p53−/−;GFP tumors strongly expressed GFP and were negative for MPZL1, whereas MycOE;p53−/−;MPZL1 tumors were negative for GFP but showed a strong membranous expression of MPZL1 (Fig. 5i). Characterization of immune cells revealed positivity for CD3 and LNGFR (a marker specifically expressed in our CAR-T, Fig. 4a), indicating these cells as MPZL1 CAR-T cells infiltrating the tumor core (Fig. 5i). In summary, these results demonstrate that MPZL1 CAR-T cells have high efficiency and specificity and are capable of completely eradicating MPZL1-positive tumors not only in human xenografts but also in autochthonous liver tumors in vivo.

### Human tumor-specific MPZL1 CAR-T cell infiltration and effector molecule induction

Due to the human specificity of our CAR construct and the lack of cross-reactivity with murine MPZL1, comprehensive in vivo testing in immunocompetent mice was not feasible. To address this limitation and provide a preclinical model closely reflecting human biology, we utilized a fully human T cell-tracing tissue explant system, including both MPZL1-positive tumor samples from different entities and patient-matched healthy tissue samples (Fig. 6, Supplementary Fig. 8). To this end, freshly resected explant sections were treated with CMFDA-labeled T cells (CLT) and cultivated overnight following examination of infiltration across different human patient samples (Fig. 6a, see Methods). Further, T cell activity was assessed via granzyme B (GranB) immunofluorescence staining (Supplementary Fig. 8c) and multiplex cytokine analysis. We observed that MPZL1 CAR-T cells displayed markedly higher infiltration into MPZL1-positive tumor explants compared with naïve and activated UT T cells, as well as CAR-T cells targeting carcinoembryonic antigen (CEA), ovarian cancer antigen 125 (CA125), or CD19 that we tested to additionally confirm MPZL1 CAR-T cell specificity (Fig. 6b, c). Positive tissue expression of MPZL1, CA125, and CEA in corresponding patient tissues was confirmed by IHC(Supplementary Fig. 8a, b). Of note, the high infiltration of MPZL1 CAR-T cells was accompanied by significantly elevated levels of effector molecules, including granzyme B (GranB), CXCL9 (MIG), CXCL10 (IP10), interferon gamma (IFNγ), tumor necrosis factor alpha (TNFα), and interleukin 2 (IL-2), relative to other CAR-T or UT T cells (Fig. 6d, e). Importantly, comparison with patient-matched healthy tissue explants revealed lower MPZL1 CAR-T cell infiltration and significantly reduced effector molecule induction in normal tissues (Fig. 6f–h). Together, these initial human tissue sample-based results indicate selective activity of MPZL1 CAR-T cells toward tumor cells as opposed to healthy patient-matched human tissue.

### Discussion

Targeting cancer driver alterations has been the cornerstone of precision oncology. However, many drivers are difficult to drug, and their prevalence is often limited to small patient subsets, restricting broad therapeutic impact^1,11,51^. Here, we aimed to expand the concept of targetable vulnerabilities by focusing on CSP-encoding genes located within chromosome amplifications, which are widely observed across solid tumors. Using a multiomics approach integrating chromosomal amplification data, RNA expression, and cell surface annotation, we mapped potential CSPs suitable as entry points and identified MPZL1 (myelin protein zero-like 1), a glycosylated receptor belonging to the immunoglobulin superfamily^41^, as a promising candidate for precision therapy.

MPZL1 is located on chromosome 1q, a region frequently amplified in cancers such as hepatocellular carcinoma, triple-negative breast cancer, and lung adenocarcinoma, which we found to harbor many cell surface-expressed protein-coding genes. Notably, MPZL1 is not a classical oncogenic driver, consistent with our data showing that its overexpression or knockout does not alter tumor initiation or growth in vitro or in vivo. Yet, MPZL1 has been proposed to promote migratory and invasive capabilities^35,39,40,52^. Therefore, MPZL1-directed therapy may preferentially target tumor cells with enhanced metastatic capacity, potentially providing an added therapeutic benefit. Independent of its potential contribution to cancer hallmarks, its elevated cell surface expression in tumors, relative to normal tissues, creates a therapeutic window that can be exploited. IHC across 2335 human samples confirmed strong MPZL1 membranous expression in tumors, whereas normal tissues generally showed absent or faint staining, with few exceptions in reproductive tissues.

To leverage elevated MPZL1 surface expression as a therapeutic target, we developed a high-affinity antibody against its extracellular domain. A major limitation of this MPZL1-ChAb is that it does not react with murine MPZL1 and is suitable only for flow cytometry and cannot be used to stratify patients via IHC. This lack of functionality in other techniques may be due to its recognition of conformational or discontinuous epitopes^53^. Additionally, as MPZL1 is a highly glycosylated protein, we cannot exclude the possibility that MPZL1-ChAb recognizes a specific glycosylation pattern, which could affect binding when the protein is not in its native state, or even that it targets a tumor-specific glycopattern, as it has been reported for various antibodies in the literature^54^. Anyhow, using paired FFPE tumor tissue slides and dissociated tumor cells, we found that the commercially available MPZL1 antibody, which in our hands reveals robust IHC performance, mirrors the MPZL1 protein expression detected by our newly established antibody. Thus, we anticipate that the commercial MPZL1 antibody could serve as a companion diagnostic tool to stratify patients who are likely to benefit from MPZL1-targeted therapies.

Limitations aside, we were able to use our newly developed MPZL1-ChAb to generate MPZL1-specific CAR-T cells. These CAR-T cells demonstrated potent and antigen-specific cytotoxicity across multiple MPZL1-positive human cancer cell lines in vitro. In vivo, systemic administration of a single dose of MPZL1 CAR-T cells induced regression or complete elimination of human xenograft tumors, as well as MPZL1-positive tumors in HTVI-induced autochthonous liver cancer models. Importantly, short-term analyses in human tissue explants confirmed selective infiltration and activation of MPZL1 CAR-T cells in tumor tissue as opposed to patient-matched normal tissue, supporting a favorable tumor specificity. While our studies do not fully address systemic on-target/off-tumor toxicities—given the lack of cross-reactivity with murine MPZL1, the use of immunocompromised mouse models, and the limited availability of healthy patient-matched tissue samples from multiple organs suitable for tissue explant assays—existing examples from other tumor-associated antigens suggest that limited expression in normal tissues as opposed to markedly elevated tumor-specific expression can be compatible with tolerable safety profiles for surface protein targeting therapeutic approaches^9,55–60^.

Collectively, our findings highlight that large chromosomal amplifications represent an underexplored reservoir of cell surface targets that can be effectively mined for therapeutic entry points. Exploiting these amplified genes enables the development of cancer driver–agnostic therapies, broadening the scope of precision medicine to a wider range of solid tumors irrespective of tissue origin. Although additional studies will be required to fully address potential safety considerations, the successful development of MPZL1 CAR-T cells that eradicate MPZL1-positive tumor cells described here demonstrates the feasibility of this strategy and provides a blueprint for targeting amplified CSPs in human cancers.

## Methods

### Animal ethics statement

All animal experiments were conducted in compliance with regional regulations and approved by the regional authority (Regierungspräsidium Karlsruhe, Germany) under permit numbers G-70/20 and G52-25. Housing conditions for the mice included a 12-hour light/12-hour dark cycle, an ambient temperature of 20–24 °C, and relative humidity of 45–65%. For xenograft experiments, a maximum tumor diameter of 1.5 cm was permitted by the ethics committee, and this limit was not exceeded in any experiment. For autochthonous liver tumor models, hydrodynamic tail vein injections were performed under standardized conditions, and animals were monitored carefully during post-injection recovery. Mice were regularly palpated and inspected for abdominal enlargement and general well-being. Animals were euthanized upon reaching predefined humane endpoint criteria in accordance with institutional and governmental regulations.

### Patient tissues and ethics

Institutional Ethical Review Board approval was obtained from the local Ethical Committees of the Medical Universities of Heidelberg (S205-06) and of the University Medical Center Mainz (2025-18147) in compliance with the Helsinki Declaration.

Patient tissue samples were provided by the Surgery Department and Women´s Hospital of the University Hospital Heidelberg and the University Medical Center Mainz. For naive allogeneic T cells, peripheral blood of anonymous healthy donors was obtained from the “DRK-Blutspendedienst” in Mannheim, Germany. Buffy coats from anonymous healthy donors to obtain T cells for CAR-T experiments were obtained from the Zentrum für Klinische Transfusionsmedizin Tübingen (ZKT) and the Blutspendezentrale IKTZ Heidelberg, respectively located in Tübingen and Heidelberg. Consent of all patients was recorded prior to analysis. No compensation was provided. Sex, gender, age, and ethnicity were not explicitly considered.

### Integrative in silico approach for CSP target identification

Human CSP-coding genes, encoding the respective CSPs annotated in the CSPA (*N* = 1492)^21^, were surveyed for their genomic status in a publicly available pan-cancer dataset (ICGC/TCGA) comprising copy number variation (CNV) data from 2703 patients^22^. The analysis was done via the online platform cBioPortal, using the Onco Query Language “GAIN AMP”^61^. This tool employs GISTIC 2.0, an algorithm that follows a statistical approach to identify significant copy-number alterations in cancer genomes^62^, assigning the following scores: −2 (homozygous deletion), −1 (heterozygous deletion), 0 (diploid state), 1 (copy-number gain) or 2 (high-level amplification). We considered amplified genes as those with assigned scores of 1 or 2. The chromosomal locations of the hits were retrieved using the *BioMart data mining tool* from Ensembl^63^. To identify tumor types with recurrent amplifications of chromosome 1q, publicly accessible copy number variation data from all cancer entities available at TCGA Firehose Legacy was explored with the Integrative Genomics Viewer Software (IGV)^25,64^. For cancer types with frequent chr1q amplification, the CNV status of the identified hits was surveyed in the corresponding datasets (HCC, *n* = 370; BRCA, *n* = 1080; LUAD, *n* = 516) via cBioPortal^26–28^. Next, differential expression analysis was performed using GEPIA2^29^, a web server that contains RNA sequencing data from TCGA and GTEx projects^65,66^. The ANOVA method was employed to obtain differential expression data for all genes under study, depicting the mRNA expression in tumor (T) relative to corresponding normal (N) tissues – HCC (T:*n* = 369; N:*n* = 160), BRCA (T:*n* = 1085; N:*n* = 291), LUAD (T:*n* = 483; N:n = 347). Next, subcellular localization of differentially expressed CSPs was retrieved from online protein databases, such as UniProt^30^, HPA^23,24^, and Gene Ontology^31^. Focusing on MPZL1 as a CSP target, the correlation between its CNV status and mRNA expression levels in the same datasets was explored via cBioPortal, considering only samples with both data types available (HCC, *n* = 360; BRCA, *n* = 960; LUAD, *n* = 230). Additionally, the CNV status of MPZL1 in human cancer cell lines was retrieved from the Cancer Cell Line Encyclopedia^67^. Publicly available data from DepMap^38^ were analyzed to assess whether MPZL1 exhibits cancer driver–like properties by evaluating gene essentiality, dependency scores, and associations with cell proliferation across human cancer cell lines.

### Cell culture

All human cancer cell lines were originally obtained from ATCC/DSMZ/JCRB and incubated at 37 °C with 5% CO_2_ and maintained in sterile conditions. Depending on the specific cell line, high-glucose Dulbecco′s Modified Eagle′s Medium (DMEM, Sigma-Aldrich) or Roswell Park Memorial Institute Medium (RPMI 1640, Gibco^TM^) was used, in every case supplemented with 10% FCS (Gibco^TM^) and 1% penicillin/streptomycin (10,000 U/mL penicillin and 10 mg/mL streptomycin, Sigma-Aldrich). All human liver and breast cancer cell lines, as well as U-87 MG and CFPAC-1, were cultured in DMEM medium, while all human lung cancer cell lines, as well as NALM6 and Molm13, were cultured in RPMI medium.

Murine liver cancer cell lines (Kras^G12D^; p53^−/−^) were derived from tumors generated via HTVI in female C57Bl/6 animals^18^. Murine cells were cultivated in DMEM, with 10%FBS and 1% penicillin/streptomycin on collagen-precoated (PureCol, Cell Systems; 0.05 mg/ml) dishes (Greiner).

### Genetic modification of cancer cell lines

For CRISPR/Cas9-mediated knockout of MPZL1, sgRNAs were designed using the CHOPCHOP web tool^68^, and subsequently cloned into the pLentiCRISPR v2 vector^69^, as previously described^70^. For lentiviral overexpression of MPZL1, NEBuilder^®^ HiFi DNA Assembly was used to introduce an MPZL1 PCR product, generated from pENTR223-MPZL1 (obtained from DKFZ core facility), into the pLenti6.2/V5-DEST vector. Lentivirus production, transduction of cancer cells, and antibiotic-based selection were performed as previously described^70^, generating isogenic cancer cell lines either lacking or overexpressing MPZL1 protein. Finally, the efficiency of genetic modification was assessed via immunoblotting.

- sgMPZL1.1_top guide (5’ → 3’): CACCGGGGGGCCGACACTACTGT
- sgMPZL1.1_bottom guide (5’ → 3’): AAACACACAGTAGTGTCGGCCCC
- sgMPZL1.2_top guide (5’ → 3’): CACCGGCACCGACCACAGCCAGC
- sgMPZL1.2_bottom guide (5’ → 3’): AAACGCGCTGGCTGTGGTCGGTG

### Immunoblotting

Harvested cells were lysed in lysis buffer (Cell Signaling Technology), supplemented with protease (cOmplete^TM^ Mini, Roche) and phosphatase inhibitors, by incubation on ice for 30 minutes, followed by centrifugation at 16,000 × *g* to collect protein lysates. Quantification of protein was done using the Bradford reagent (Bio-Rad). Samples with equal concentration were prepared with Laemmli buffer^70^ and denatured by boiling at 95 °C for 5 minutes. 20 μg protein samples were separated via SDS-PAGE using manually cast polyacrylamide gels and EZ-Run™ protein ladder (Fisher BioReagents™) as an indicator of molecular weight. Separated proteins were transferred to ROTI^®^PVDF membranes (Carl Roth) via Western Blot. Membranes were then blocked for 1 hour in 5% Milk (Carl Roth), followed by incubation with the primary antibody overnight at 4 °C (Supplementary Table S5). The day after, membranes were washed with TBS/T buffer and incubated with the corresponding HRP-conjugated secondary antibody (Supplementary Table S6). for 1 hour at RT, followed by additional washing and chemiluminescent signal detection using the Clarity™ Western ECL Substrate (Bio-Rad) and ChemiDoc^TM^ Gel Imaging System (Bio-Rad). Quantification of protein bands was done via densitometry-based analysis with ImageJ, where the relative intensity of MPZL1 was normalized to that of Vinculin, used as a loading control.

### Deglycosylation assay

Deglycosylation of protein lysates was achieved by treatment with PNGase F enzyme. First, denaturation reactions were prepared by mixing 20 µg of lysate and 1 µL of Glycoprotein Denaturing Buffer (NEB) in a final volume of 10 µL of H_2_O. Samples were then boiled at 100 °C for 10 minutes. Second, deglycosylation reactions were set up by adding 2 µL of GlycoBuffer (NEB), 2 µL of NP-40 (NEB), 6 µL of H_2_O, and 1 µL of PNGase F (NEB), followed by incubation at 37 °C for 1 hour. Non-deglycosylated samples were subjected to the same denaturation protocol as an internal experimental control. Samples were further analyzed via immunoblotting.

### Proliferation assays

For the clonogenic assay, 1 × 10^3^ cells were plated in triplicate into six-well plates and allowed to grow for 2 weeks, with regular medium exchange. The cell colonies were then fixed and stained with a 0.05% (w/v) crystal violet solution containing 1% formaldehyde. Plates were scanned to generate digital images using the Perfection V850 Pro scanner (Epson). For the short-term proliferation assay, 1 × 10^3^ cells were seeded in triplicate into independent 96-well plates corresponding to the different time points (0, 24, 48, 72, and 96 hours). The readout was performed every 24 hours using CellTiter-Blue^®^ (Promega), following the manufacturer’s instructions. After incubation for 4 hours at 37 °C, fluorescence intensity (560/590 nm) was measured using the EnSpire^®^ Multimode Plate Reader (PerkinElmer). *Time 0* was defined as the day after plating the cells, and the results normalized to this time point.

### Cell fractionation assay

To investigate the subcellular localization of MPZL1, the Minute^TM^ Plasma Membrane Protein Isolation and Cell Fractionation Kit (Invent Biotechnologies) was used. Cells grown in 15 cm dishes were harvested and processed according to the manufacturer’s protocol. After multiple rounds of centrifugation and incubation in the buffers provided, the different protein fractions were obtained: cytosol, organelle membrane, and plasma membrane. Additionally, *“whole cell lysates”* from the same cell lines were prepared as experimental controls. Samples were analyzed via immunoblotting, using tubulin and Na/K-ATPase as markers for cytosolic and plasma membrane fractions, respectively.

### MPZL1 CSP target in a human patient cohort

Thirteen samples of patient livers were obtained upon tissue surgical resection^71,72^. Copy number variation status of the MPZL1 gene was assessed via comparative genomic hybridization. Next, MPZL1 mRNA expression was evaluated based on microarray data^71,72^, while MPZL1 protein levels were assessed through immunoblotting.

### Immunohistochemistry

IHC was performed as previously described^70^. Briefly, deparaffinization of tissues was achieved by incubating the slides in xylene, followed by rehydration using a descending series of alcohol and a washing step with water. For antigen retrieval, slides were boiled for 8 minutes in a pressure cooker using a sodium citrate buffer (10 mM trisodium citrate dihydrate, 0.5%(v/v) TWEEN 20, pH 6.0), followed by rinsing in water for 5 minutes to cool down. Next, endogenous HRP was blocked by a 10-minute incubation in 3% hydrogen peroxide diluted in H_2_O. The slides were then rinsed in water for another minute and washed twice for 2 minutes with PBS. Tissue sections were blocked for 1 hour at room temperature in 5% BSA (Carl Roth) containing 0.5% Triton X-100, followed by overnight incubation at 4 °C with the corresponding primary antibody diluted in blocking buffer (Supplementary Table S7). Then, the slides were washed three times with PBS containing 0.05% Triton X-100 for 5 minutes, and incubated with the corresponding ImmPRESS^®^ HRP Horse IgG Polymer Detection Kit, Peroxidase (Anti-Rabbit or Anti-Mouse) (VectorLabs) for 30 mins at RT, again followed by three 5-mins washing steps with PBS/Triton X-100. The antibody signal was detected with ImmPACT^®^ DAB/HRP substrate (VectorLabs), according to the manufacturer’s instructions, with the reaction time adjusted individually for each antibody. Counterstaining with hematoxylin solution (Carl Roth) was then carried out for 1 or 2 minutes to obtain improved staining contrast. The slides were rinsed with running water and dehydrated using an ascending alcohol series, finishing with xylene. When dried, the coverslips were mounted on the slides using Micromount^®^ mounting media (Leica). Histological slides were scanned with the Hamamatsu NanoZoomer Digital Pathology (NDP) system, and visualized and analyzed using QuPath^73^ and FiJi ImageJ^74^ software.

### Tissue microarray (TMA) analysis

The expression and subcellular localization of MPZL1 in primary human tissue were analyzed by IHC using TMAs, comprising cancer-specific and non-cancer tissue punches (Supplementary Table S8). The histochemical staining was quantified by an experienced pathologist using a scoring system to evaluate MPZL1 expression (MPZL1-score) on the surface of epithelial cells, where 0 indicates negative staining, 1 and 2 represent intermediate levels, and 3 corresponds to complete plasma membrane staining in >10% of the cells (Fig. 2d). Institutional Ethical Review Board approval was obtained at local Ethical Committees of the Medical Universities of Heidelberg (S205-06), in compliance with the Helsinki Declaration. Written informed consent was obtained from all individuals.

### Generation of an antibody targeting MPZL1

The hybridoma technology was used to generate antibodies against the extracellular domain of MPLZ1 protein. Briefly, 6 mice (3 each from strain Balb/C or C57BL6/N) were immunized with the extracellular fragment of human MPZL1 protein (extracellular domain of human MPZL1 protein; aa 36-165: SALEV YTPKEIFVAN GTQGKLTCKF KSTSTTGGLT SVSWSFQPEG ADTTVSFFHY SQGQVYLGNY PPFKDRISWA GDLDKKDASI NIENMQFIHN GTYICDVKNP PDIVVQPGHI RLYVVEKENL PVFPV in E. coli (pQE-vector system, HIS tag) by 4 subcutaneous injections (day 0 with Freund’s Adjuvant complete, day 7 with Freund’s Adjuvant incomplete, day 28 and 32 with Quil A, day 33 test sera, day 34 fusion; The immune response was tested by Western blot using cell lysates form overexpressed hMPZL1 fused with a tag. B lymphocytes of the animal showing the strongest immune response in Western blotting were isolated and fused with immortal myeloma cells to generate hybridoma clones capable of producing unique monoclonal antibodies (mAbs), and the clones were evaluated via FACS. The identification of a positive clone was followed by two rounds of subcloning, and the most specific subclone against MPZL1 was selected. Subsequently, the RNA from this hybridoma was sequenced to identify the variable heavy (V_H_) and *kappa* light (V_L_) chains (Supplementary Table S9–12). These sequences were cloned in frame into two independent human backbone vectors coding for the constant heavy (C_H_) and *kappa* light (C_L_) chains^75^, respectively. Finally, PEI-mediated transfection of HEK293 cells with these two plasmids led to the production of MPZL1-ChAb, composed of a human constant domain and a mouse variable region

### Cell viability assay

To evaluate cellular susceptibility to MPZL1-ChAb, 1 × 10^3^ cells per well were seeded in a 96-well plate and allowed to attach for 24 hours. Consecutively, treatments with increasing concentrations of MPZL1-ChAb (0.05–100 μg/mL) were applied in triplicate, with DMEM medium as the negative control. The readout was performed after 72 hours using CellTiter-Blue^®^ (Promega), following the manufacturer’s instructions. Fluorescence intensity (560/590 nm) was measured with the EnSpire^®^ Multimode Plate Reader (PerkinElmer).

### Cell surface staining and FACS analysis

Extracellular staining of MPZL1 in isogenic cancer cell lines was performed to evaluate the specificity of MPZL1-ChAb. Briefly, adherent cells were washed with PBS and incubated in Versene-EDTA (Lonza) at RT for 10 to 15 minutes prior to harvesting using FACS buffer (3% FCS in PBS). 3 × 10^5^ cells per condition were transferred to previously annotated wells in a V-bottom 96-well plate. Upon washing, cells were stained with 100 µg/mL MPZL1-ChAb for 45 minutes at 4 °C. Next, cells were washed and stained with a secondary antibody (AF488^®^ or APC anti-human IgG Fc, BioLegend) during 45 minutes at 4 °C in the dark. After a final washing step, cells were resuspended in 200 µL FACS buffer containing 1:1000 SYTOX^®^ Blue (Invitrogen), and transferred to cell strainer snap cap FACS tubes (Corning) for FACS analysis using a BD LSRFortessa flow cytometer (BD Biosciences). As negative controls for specificity, all the experimental samples were stained in parallel with human IgG1 isotype (BioLengend) at the same concentration as the primary antibody. When CD19 staining was included, the samples underwent a third incubation round with PE anti-human CD19 antibody (BioLegend) during 20 min in the dark. The same protocol was followed to evaluate MPZL1 expression in cell lines from different cancer types, as well as in CAR-T cells. Data analysis was, in all cases, carried out with FlowJo v10.

### FFPE tissue blocks and matched DTCs

Cryopreserved dissociated tumor cells (DTCs) and formalin-fixed paraffin-embedded (FFPE) tissue blocks from human breast tumors of five independent patients (Discovery Life Sciences) were used to investigate MPZL1 expression on the surface of cancer cells within the tumor architecture. Anonymous patients were named as: 8843, 4130, 8921, 2457, 8991. MPLZ1 expression on tissue slides obtained from the FFPE blocks was evaluated via IHC with MPZL1 commercial antibody. DTCs were thawed and stained with fluorochrome-conjugated MPZL1-ChAb to measure antibody binding capacity (ABC) on the cell surface via FACS analysis.

### Measurement of ABC

To quantitatively measure the ABC in DTCs, both MPZL1-ChAb and human IgG isotype control (Invitrogen) were covalently labeled with the AF488^®^ fluorochrome, using the AlexaFluor^TM^ 488 protein labeling kit (Invitrogen), according to the manufacturer’s instructions. Following labeling and purification, the sample absorbance was determined utilizing a Nanodrop ND-1000 Spectrophotometer (Thermo Fisher Scientific), with wavelengths of 280 nm for the protein and 494 nm for the dye. The protein concentration and the degree of labeling (DOL), representing the moles of dye bound per mole of protein, where calculated following protocol provided in the kit. For cell surface staining, 1 × 10^5^ DTCs per condition were used, with JIMT-1 and Molm13 cells included as positive and negative controls, respectively. All samples were first incubated with 5 µg/mL of Human BD Fc Block^TM^ (BD Pharmingen^TM^) for 10 minutes at RT to minimize nonspecific antibody binding to Fc receptors. Directly afterwards, cells were incubated with 5 µg/mL of labeled antibody for 1 hour at 4 °C in the dark. After two washing steps with PBS, cells were resuspended in 200 µL FACS buffer containing 1:1000 of SYTOX^®^ Blue (Invitrogen). Samples were acquired in a BD LSRFortessa flow cytometer (BD Biosciences). A calibration curve was built to correlate the instrument’s acquisition channels to standardized fluorescence intensity units known as MESF (molecules of equivalent soluble fluorochrome), employing the Quantum™ Alexa Fluor^®^ 488 MESF kit (Bangs Laboratories), according to the manufacturer’s instructions. This calibration enabled the interpolation of MESF values from the median channel values obtained from the acquired samples. Finally, the ABC in each sample was calculated dividing the MESF value by the DOL value previously determined. Additionally, the ABC for the IgG isotype control was subtracted from that of the MPZL1-ChAb in each sample to account only for specific target binding. This method allowed to determine the binding efficiency of MPZL1-ChAb to patient cancer samples with various levels of MPZL1 expression on the cell surface of cancer cells.

### Generation of MPZL1-specific CAR constructs

The scFv generated is composed of the variable regions of the heavy and light chains of MPZL1-ChAb (Supplementary Table S9-12), connected by a short linker peptide. Two distinct approaches were followed, resulting in V_L_ + V_H_ and V_H_ + V_L_ configurations, with two constructs designed for each combination. Briefly, MPZL1.24 and MPZL1.66 contain one additional amino acid at the C-terminus compared with MPZL1.23 and MPZL1.44, reflecting the inclusion of terminal nucleotides of the MPZL1-ChAb sequence whose coding status could not be unambiguously resolved during sequence verification. The MPZL1-scFv is preceded by a leader sequence and followed by a hinge-transmembrane region, along with the CD28 and CD3ζ domains. The construct also includes the expression of a reporter gene (LNGFR). Briefly, these components were incorporated into a SFGγ retroviral vector, which was used for the stable transfection of gpg29 (H29) fibroblasts (kindly provided by Michel Sadelain (Columbia University) to J.F^76,77^. The generated retroviral supernatants were employed to establish stable retrovirus-producing Phoenix-AMPHO cell lines, and the resulting supernatants utilized for the transduction of T cells^77^.

### Isolation and expansion of human T cells

Buffy coats from anonymous healthy human donors were purchased from either the ZKT Tübingen or the IKTZ Heidelberg (Zentrum für Klinische Transfusionsmedizin, and Institut für Klinische Transfusionsmedizin und Zelltherapie, respectively). Peripheral blood mononuclear cells (PBMCs) were separated with Lymphocyte Separation Medium (Corning) via density gradient centrifugation. Isolated PBMCs were cryopreserved in FCS containing 10% DMSO. T cells were purified from thawed PBMCs by negative selection using magnetic-activated cell sorting (MACS) with a Pan T Cell Isolation Kit (Miltenyi Biotec), following the manufacturer’s instructions. Isolated T cells were stimulated with CD3/CD28 human T cell Activator Dynabeads (Gibco) in a 1:1 ratio, and plated at 1 × 10^6^ cells per mL in RPMI medium supplemented with FCS and penicillin/streptomycin, and 5 ng/mL of interleukin-7 (IL7) and interleukin-15 (IL15) cytokines (Miltenyi Biotec). Specific stimulation continued for 72 hours before proceeding to T-cell genetic modification. Cells were in all cases maintained in sterile conditions and incubated at 37 °C with 5% CO_2_.

### Genetic modification of T cells

After 72 hours in culture, the Dynabeads were magnetically removed from the activated T cells. Subsequent T cell transduction was performed utilizing recombinant human fibronectin fragment (RetroNectin^®^, Takara Bio), which aids in the co-localization of target cells and viral particles when subjected to spinoculation. Briefly, non-tissue culture-treated multi-well plates were coated with 15 µg/mL RetroNectin^®^ diluted in PBS for 2 hours at RT. The coating solution was then removed, and the following transduction components were added: (1) CAR-coding retroviral supernatant, (2) T cells at a concentration of at 1 M T cells/mL supplemented with 5 ng/mL of IL7 and IL15 cytokines, and (3) RPMI medium to reach the same final volume across all conditions. Spinoculation took place for 1 hour at 130 g and 37 °C. Upon transduction, the cytokine-supplemented medium was replenished every second day.

### CAR expression, proliferation and viability of unstimulated CAR-T cells

CAR-T cells are defined as unstimulated when they have not encountered their specific antigen, and are maintained in culture in the presence of IL7 and IL15 cytokines. Expression of the CAR construct on the surface of T cells was determined via flow cytometry starting from day four after transduction. T cells were stained with both AF647^®^ goat anti-mouse IgG F(ab’)2 fragment specific (AffiniPure/Jackson Immuno) and PE mouse anti-human CD271 (LNGFR, BD Biosciences). Cell acquisition was performed on a Cytek^®^ Aurora (Cytek Biosciences) or a LSRFortessa (BD Biosciences) flow cytometer, and subsequent data analysis was carried out with FlowJo v10. For proliferation and viability assays, T cells were counted using an automated cell counter (Countess™, Invitrogen) at multiple time points after transduction, and viability percentages were automatically calculated based on trypan blue exclusion.

### Bioluminescence-based cytotoxicity assays

To generate target cells, isogenic cancer cell lines were stably transduced to express firefly luciferase (FFLuc) linked to GFP protein. The GFP+ population was sorted using a BD FACSAria™ Fusion cell sorter (BD Biosciences) to have a pure population of Luc-expressing cells. For the cytotoxic assays, 2 × 10^4^ adherent target cells per well were seeded in black-walled transparent-bottom 96-well plates and incubated at 37 °C and 5% CO_2_ during 24 hours to allow for attachment. After that, medium was removed, and the co-culture with CAR-T cells was established in triplicate at the indicated range of effector-to-target cell ratios (E:T ratio), in a final volume of 100 µL/well. In every case, the required amount of T cells was calculated based on their percentage of CAR-positive T cells, adjusting the total number of effector cells across treatments via compensation with UT T cells. Target cells alone and 0.2% Triton X-100 were utilized for the minimal and maximal lysis controls (RLU, relative light units), respectively. In the case of target suspension cells, no pre-seeding was required. Following the co-culture of cells during 4 or 18 hours (short- and long-term killing assays, respectively), 100 µL of D-luciferin (ZellBio, GoldBio) diluted 1:10 in PBS was added per well. Bioluminescence was measured using a microplate reader (Infinite^®^ 200 PRO, Tecan), and cell lysis was determined as (1 − (RLU_sample_)/(RLU_max_)) × 100.

### Phenotypic characterization

For phenotypic analysis, flow cytometry was performed after staining MPZL1 CAR-T cells with the following mouse anti-human fluorochrome-conjugated antibodies: BUV395 CD4, BV650 CD45RA, and PE CD271 (LNGFR) (BD Biosciences); PE/Cyanine7 CD8a, and PerCP-eFluor 710-LAG3 (Invitrogen); and BV510 CD25, BV711 CD69, APC/Cyanine7 PD-1, and BV421 CD62L (BioLegend), along with 7-AAD cell viability solution (BD Biosciences). Besides, compensation controls using UltraComp eBeads^TM^ (Invitrogen) and fluorescence minus one (FMO) controls were included. Cell acquisition was done on a Cytek^®^ Aurora flow cytometer (Cytek Biosciences), and subsequent data analysis was carried out with FlowJo v10.

### Antigen-dependent proliferation and intracellular cytokine secretion assay

Antigen stimulation assays consisted in the co-culture of MPZL1 CAR-T cells with either MPZL1^high^ JIMT-1 breast cancer cells or the respective *MPZL1* knockout cells. For the antigen-dependent proliferation assay, JIMT-1 target cells were irradiated with a dose of 30 grays during 4 minutes to inhibit their proliferative capacity prior to the co-culture. Next, 3 × 10^5^ irradiated cells per well were seeded in 24-well plates, and incubated during 24 hours to allow for attachment. DMEM was removed after one day, and the co-culture was established in triplicate wells by adding 1 × 10^6^ MPZL1 CAR + T cells without cytokines in 500 µL of RPMI per well, with fresh RPMI added every other day. T-cell counts were performed every six days with a cell counter (Countess™, Invitrogen). Subsequently, the co-culture was reestablished by adding 1 × 10^6^ prestimulated CAR-T cells (after a 6-day stimulation period) to freshly irradiated target cells. Restimulation was conducted over three consecutive 6-day periods, with the cumulative number of CAR + T cells calculated as the product of the current CAR-T cell count and the accumulated expansion factor, which incorporates the growth rates of previous stimulations. For the intracellular cytokine secretion assay, a 16-hour co-culture of 5×10^5^ MPZL1 CAR-T cells and 5×10^5^ respective JIMT-1 target cells, seeded one day in advance, was established in 24-well plates. Treatment with PMA/Ionomycin was utilized as a positive control to induce cytokine secretion. During the last 4 hours, a cocktail of Brefeldin A and Monensin (protein transport inhibitor cocktail, Invitrogen) was added to the co-cultures to retain the cytokines within the cells. Next, T cells were washed, stained for cell surface markers (phenotypic analysis), fixed and permeabilized with a fixation and permeabilization buffer set (Invitrogen), according to the manufacturer’s instructions. Intracellular cytokine staining was performed with the following antibodies: APC Mouse Anti-Human/Mouse GZMB and PerCP Rat Anti-Human IL-2 (BioLegend), and BUV737 Mouse Anti-Human IFNγ and BV650 Mouse Anti-Human TNFα (BD Biosciences), along with ViaDye™ Red Fixable Viability Dye (Cytek Biosciences). Both compensation and FMO controls were included. Cell acquisition was done on a Cytek^®^ Aurora flow cytometer (Cytek Biosciences), and subsequent data analysis was carried out with FlowJo v10.

### Human cell line-derived mouse xenograft model

Tumors of human origin were established in immunodeficient 7- to 8-week-old NSG mice (Janvier). To this end, 5 × 10^6^ genetically manipulated cells in 100 μL of PBS were subcutaneously injected into each flank of the mouse under isoflurane anesthesia. The xenograft tumors were regularly measured with a caliper, and tumor volume was calculated as (length×(width)^2^)/2. When the average tumor volume reached a specific size (120 mm^3^ for breast and 360 mm^3^ for HCC xenografts), a single dose of 1 × 10^7^ MPZL1 CAR-T cells was intravenously administered to all the animals in the experiment on the same day (time 0). MPZL1 CAR-T cells used for animal treatments were always administered directly after primary ex vivo expansion. Tumor size was monitored over time, and the mice were euthanized by cervical dislocation when any of their tumors reached a maximum diameter of 1.5 cm or, alternatively, if ulcerations appeared. The tumors were resected, photographed, weighed, and finally fixed in 4% paraformaldehyde (PFA) for further processing in IHC applications.

### HTVI-induced autochthonous HCC mouse model

Tumors were generated directly in the livers of 7–8-week-old NSG mice by hydrodynamic tail vein injection (HTVI), where 2 mL of NaCl solution (~10% of the mouse body weight) containing naked plasmid DNA was rapidly injected (within 5–7 seconds) into the tail vein, enabling efficient transfection of hepatocytes in vivo^78^. Tumor formation was monitored following overexpression of c-MYC, alongside human MPZL1, or NICD or mutant CTNNB1 employing the Sleeping Beauty DNA transposon system^79^ (Supplementary Fig. 2). Tumor formation was induced by overexpression of c-MYC, coupled with either human MPZL1 cDNA or GFP, employing the Sleeping Beauty DNA transposon system^79^, and CRISPR/Cas9-mediated KO of p53, with the respective plasmids generated as previously described^70^. (Fig. 5; Supplementary Fig. 7). The groups were respectively named MycOE;p53−/−;MPZL1 and MycOE;p53−/−;GFP. For the intervention experiment, a single dose of 1×10^7^ MPZL1 CAR-T cells was intravenously administered to all the animals 15 days after HTVI, and tumor formation was evaluated by regular palpation of the mice's bellies. MPZL1 CAR-T cells used for animal treatments were always administered directly after primary ex vivo expansion. When tumors were detected, the mice were euthanized by cervical dislocation. The livers were resected, and bright-field and GFP images were acquired using a stereomicroscope (MZ10F, Leica), and fixed in 4% PFA to be processed for IHC studies. In the short-term experiment, a single dose of 1×10^7^ MPZL1 CAR-T cells was intravenously administered one month after HTVI. One week after CAR-T treatment, all animals were sacrificed and the livers underwent the same processing.

### Preparation of naïve T cells, CEA and CA125 CAR-T cells for explant model

For preparation of autologous naive UT T cells, an approximately 0.5 cm² piece of macroscopically detected adjacent liver of liver metastasis explants was placed in a Petri dish and minced with a scalpel. Sheared tissue was gently added on a cell strainer (40 μm mesh) and flushed into a 50 ml falcon tube using RPMI (Sigma-Aldrich). Flow-through was flushed a second time through a new cell strainer, and isolated cells were stained with 5 µM CellTracker™ Green CMFDA (Thermo Fisher Scientific) for 1 hour in cell culture flasks. Non-adherent CMFDA-labeled T cells (CLT) were isolated by negative bead-based isolation using the Dynabeads Untouched Human T Cells Kit (Thermo Fisher Scientific). Autologous naïve UT-CLT were directly re-suspended in explant culture medium and added to the corresponding patient-matched explants without pre-activation.

Naïve allogeneic UT T cells were prepared from peripheral blood of a healthy donor by density gradient centrifugation using Ficoll Paque Plus (Sigma-Aldrich) following 1.5 hours separation of non-adherent cells in cell culture flasks and negative bead-based T cell isolation. T cells were cultured for 48 hours in X-vivo 15 medium (Biozym) with anti-human CD3 antibody (1:10,000, clone OKT3, BioLegend) and addition of 300 Units IL-2 (PeproTech). T cells were stained with 5 µM CMFDA for 1 hour and cryopreserved in FBS (Biochrom) with 10% DMSO. Frozen allogeneic naive UT-CLT were thawed, directly re-suspended in explant culture medium, and added to the explant cultures without re-activation.

CEA and CA125 CAR-T cells were manufactured from peripheral blood of appropriate patients prior to surgery, as documented below, activated for 48 hours by TransAct™ in TexMACS™ medium containing IL-7 and IL-15 (all from Miltenyi Biotec), stained with CMFDA, resuspended in explant culture medium, and added to patient-matched explants. Patient-matched UT T cells were generated similarly from the same peripheral blood samples but without CAR transduction.

### Explant model and T-cell tracing

Fresh resected patient tissues were cultured as follows: explants were directly transferred from the operating room to the laboratory in 0.9% sodium saline solution and on ice. Each explant was cut into small pieces containing equal proportions of macroscopically detected tumors. One piece of explant was directly cryopreserved in OCT embedding compound (VWR) and stored as a control. Explant pieces were placed into 96-well plates containing MEM culture medium supplemented with 1% L-GlutaMAX (Thermo Fisher Scientific) and cultured under sterile conditions at 37 °C and 5% carbon dioxide. For T cell tracing, the medium of the explant cultures was removed and CLT were gently added with fresh medium. As a negative control for each patient, one explant piece received only fresh culture media without cells (untreated control). After approximately 24 hours, explants were harvested and cryopreserved in OCT embedding compound^80,81^.

MPZL1 and CD19 CAR-CLT were added to the explants directly after CMFDA staining. For treatment of the same patients, CAR-CLT were compensated with corresponding UT-CLT to align ratios of CAR expression. Seeded cell numbers varied between 1 × 10^5^ and 1 × 10^6^ cells (for CAR-CLT and UT-CLT) and between 1 × 10³ and 1 × 10^7^ cells (for autologous naive UT-CLT) depending on individual T cell isolation yields, number of living cells, and number of explants treated. For CEA CAR-CLT, the corresponding colorectal cancer liver metastasis tissue was preserved in cryopreservative media (Lonza), thawed, and rinsed for 24 hours in MEM before culture. In total 27 patients with 6 different tumor entities (1xCRC: Colorectal Cancer, 21xHLM: Colorectal Cancer Liver Metastases, 1xPaCa: Pancreatic Ductal Adenocarcinoma, 2xOv: Ovarian Cancer, 1xGaCa: Gastric Cancer and 1xRCC: Renal Cancer) were treated including 6 normal tissues (3xLiver, 1xColon, 1xSpleen, 1xStomach) with 5 corresponding tumors (2xHLM, 1xCRC, 1xGaCa, 1xPaCa) treated with MPZL1 CAR-CLT.

### IHC, immunofluorescence and image analysis

For IHC and IF on frozen tissues, 5 µm cryo sections were prepared and fixed either for 5 min in Methanol containing 30% acetone (Anti-CEA and -CA125) or for 20 min in 4% PFA, following six wash steps in PBS for 10 min (Anti-GranB). All sections were incubated for 5 min in Tris-buffered saline with 0.1% Triton-X (TTX) prior to staining. 10% normal goat serum (Vector Laboratories) was used for blocking nonspecific binding molecules. Brightfield immunostaining (DAB) with monoclonal antibodies recognizing CEA (CD66e, rabbit, 1:200, #11077-R327, Sino Biological) and CA125 (MUC16, mouse, 1:200, #NCL-L-CA125, Leica) was performed with the BondTM Polymer Refine Detection Kit (Leica) in the BOND-MAX immunostainer (Leica). Brightfield imaging was performed using the fully automatic microscopic image scanning system Hamamatsu NanoZoomer Digital Pathology (NDP) with the NDP-Viewer Software (Hamamatsu). For immunofluorescence staining, Alexa Fluor 647-conjugated monoclonal antibody against granzyme B (D6E9W, rabbit, 1:100, #71535, Cell Signaling) was applied for 1 hour following 3 wash steps with TBS and mounting with Fluoromount-G with DAPI (Thermo Fisher Scientific). For samples treated with naïve UT-CLT, CEA- and CA125 CAR-CLT, single DAPI staining was performed. Fluorescence signals were detected with the NDP system using FITC, Cy5, and DAPI filters. Image analysis was performed with ImageJ/Fiji^74^ and HALO software (Indica Labs). To reduce bias from CLT infiltration, only regions 200 µm distance from the tissue border were analyzed.

### Multiplex cytokine quantification

For cytokine analysis, the same explants as used for GranB and DAPI staining were utilized. Samples were collected by cutting explants into several 10–15 μm thick slices using a cryostat microtome, and proteins were isolated using the Bio-Plex™ Cell-Lysis-Kit (Bio-Rad Laboratories). The protein concentration of the supernatant was determined using a bicinchoninic acid (BCA) assay (Thermo Fisher Scientific), and quantification of 48 cytokines was performed in a Luminex® Reader (BioPlex System 100, Luminex® Corporation) using the Bio-Plex Pro™ Human Cytokine 27-plex and 21-plex assay kits (#M500KCAF0Y, #MF0005KMII, Bio-Rad Laboratories)^80,82^.

### Generation of CEA and CA125 (MUC-16) CAR-T cells

The scFv binders 4H11 with specificity to MUC-16 and SCA431 with specificity to CEA have been described previously^83,84^. Standard cloning techniques were used to add the scFv into the respective 2nd generation CAR backbone to create the expression cassettes pRRLSIN-4H11scFv-hIgG1-CD28-4-1BB-CDzeta and pRRLSIN-SCA431scFv-hIgG1-CD28-CD3zeta-OX40, respectively.

A second-generation packaging system was used to generate lentiviral particles. Packaging plasmids pMD2.G and pCMVR8.74 were obtained from Addgene (plasmid # 12259 and # 22036). HEK293T cells were seeded onto 15 cm plates 2 days before transfection (6 × 10^6^ cells/plate, five plates comprise one stack). Transfection of cells was done using 7.5 mM PEI in 150 mM NaCl and with equimolar amounts of plasmids (per stack: 112.5 µg pRRLSIN–39.5 µg pMD2.G–73 µg pCMVR8.74). One day after transfection, the medium was replaced with 14 ml of RPMI1640 (w/o phenol red, w/o FCS) per plate. Two days after transfection, culture supernatant (70 ml per stack) was harvested, filtered (45 µm PVDF filter membranes), and concentrated using sterilized Centricon® Plus-70 Ultracel® PL-100 devices (Merck). Lentiviral particles from one production were pooled and stored at −80 °C. Determination of infectious titer was calculated by FACS measurement of CAR expression^85^.

Primary human CD3-positive T cells were derived from healthy donor PBMCs by using the human Pan T cell Isolation Kit (Miltenyi Biotec) according to the manufacturer´s instructions. T cells were activated for 48 h by TransAct™ in TexMACS™ medium at a density of 10^6^ cells/ml containing IL-7 (0.1 mg/ml) and IL-15 (0.1 mg/ml) (all from Miltenyi Biotec). Transduction of primary CD3+ cells was done by incubation with concentrated lentiviral supernatant on day 2 after activation with a MOI of 5, in TexMACS™ Medium containing IL-7 and IL-15. Determination of CAR expression was done 48 hours after transduction by FACS using the APC-conjugated anti-CD3 antibody UCHT1 (BD Biosciences) and a PE-conjugated polyclonal anti-Fc F(ab’)2 fragment (Jackson ImmunoResearch), respectively.

### Statistics

If not indicated otherwise, statistical analyses were performed using GraphPad Prism 10 (Version 10.0.0). Respective statistical tests and exact *p* values are provided in the text and figures.

### Reporting summary

Further information on research design is available in the Nature Portfolio Reporting Summary linked to this article.

## Supplementary information

Supplementary Information

Reporting Summary

Transparent Peer Review file

## Source data

Source Data

## Data Availability

Source data are provided with this paper.
