# Supplementary material for: Cancer-driver-agnostic targeting of amplified surface proteins identifies MPZL1 for selective CAR-T cell therapy
Source: Nat Commun. 2026 Sep 10;17:9695. doi: 10.1038/s41467-026-77465-5 (PMC13562587; doi:10.1038/s41467-026-77465-5)

Supplementary Information

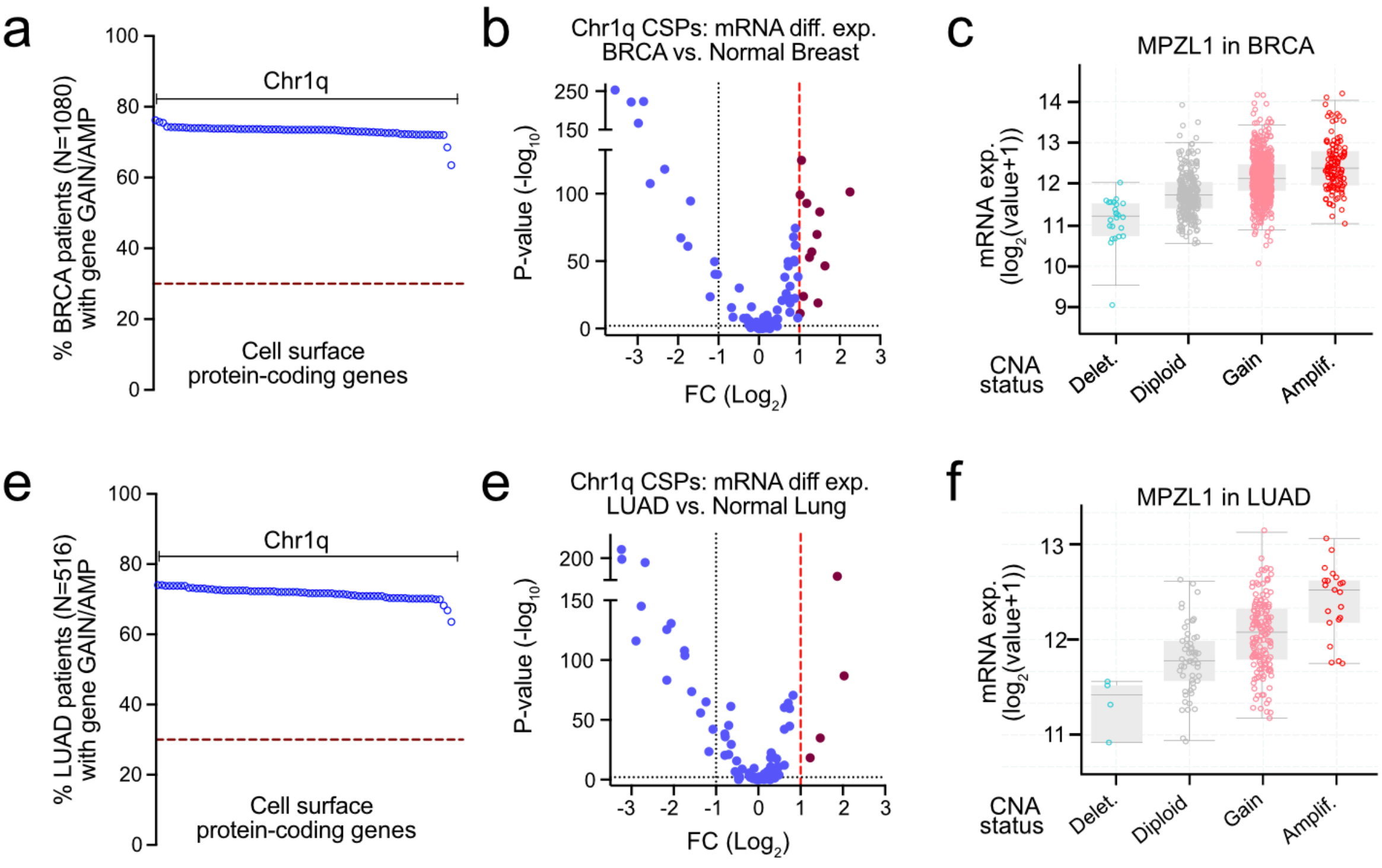

Supplementary Figure 1 | Copy number and expression analyses of CSP genes in cancers with recurrent chromosome 1q amplification.

(a–c) Breast cancer.

(a) Frequency of gain (score 1) or amplification (score 2) of CSP genes identified in Fig. 1b in breast invasive carcinoma (BRCA, n = 1,080). **(b)** Differential mRNA expression of chromosome 1q-amplified CSP genes in breast tumors (n = 1,085) versus normal breast tissue (n = 291). Data were analyzed using GEPIA2 and are expressed as log2 fold change (log2FC) along two-sided ANOVA p-values; cutoff log2FC > 1. **(c)** Association between MPZL1 CNV status and mRNA expression in the TCGA BRCA cohort (n = 960). **(d–f)** Lung adenocarcinoma. **(d)** Frequency of gain or amplification of CSP genes in LUAD (n = 516). **(e)** Differential mRNA expression of chromosome 1q-amplified CSP genes in lung tumors (n = 483) versus normal lung tissue (n = 347). Data were analyzed using GEPIA2 and are expressed as log2 fold change (log2FC) along two-sided ANOVA p-values; cutoff log2FC > 1. **(f)** Association between MPZL1 CNV status and mRNA expression in LUAD (n = 230). CNV status was defined using GISTIC2.0 as shallow deletion (−1), diploid (0), gain (1), or amplification (2). mRNA expression data were obtained from cBioPortal or GEPIA2 and expressed as log2-transformed values or log2 fold change (log2FC), as indicated. Differential expression analysis used ANOVA with cutoff log2FC > 1. Box plots show medians, interquartile range, and whiskers extending to 1.5× Interquartile Range. Source data are provided as a Source Data file.

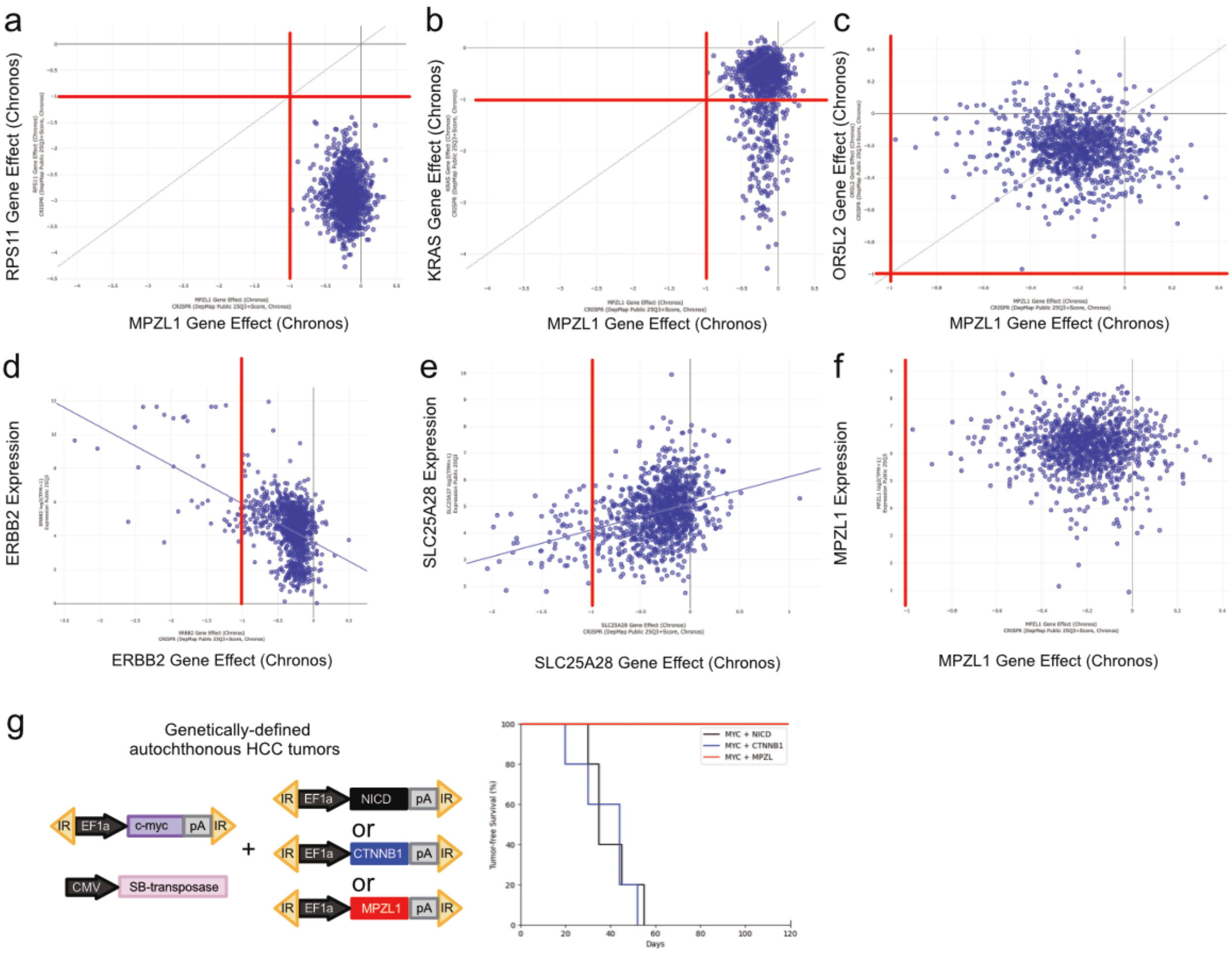

Supplementary Figure 2 | MPZL1 is not a classical cancer driver.

(a–f) DepMap CRISPR-Cas9 essentiality analysis (Chronos scores) across ~1,000 cancer cell lines. **(a)** MPZL1 KO versus RPS11 KO (core essential gene). **(b)** MPZL1 KO versus KRAS KO (essential in KRAS-mutant cell lines). **(c)** MPZL1 KO versus OR5L2 KO (non-essential gene). **(d)** ERBB2 KO versus *ERBB2* mRNA expression. **(e)** SLC25A28 KO versus *SLC25A37* mRNA expression. **(f)** MPZL1 KO versus *MPZL1* mRNA expression. A Chronos score < −1 (red lines) indicates reduced cell fitness upon gene knockout. Data are derived from DepMap public datasets.**(g)** Liver tumor formation in hydrodynamic tail vein injection (HTVI) models. Co-expression of MYC and MPZL1 does not induce tumor formation (n = 5 mice). Co-expression of MYC and NICD or MYC and mutant CTNNB1 induce tumor formation (n = 5 mice).

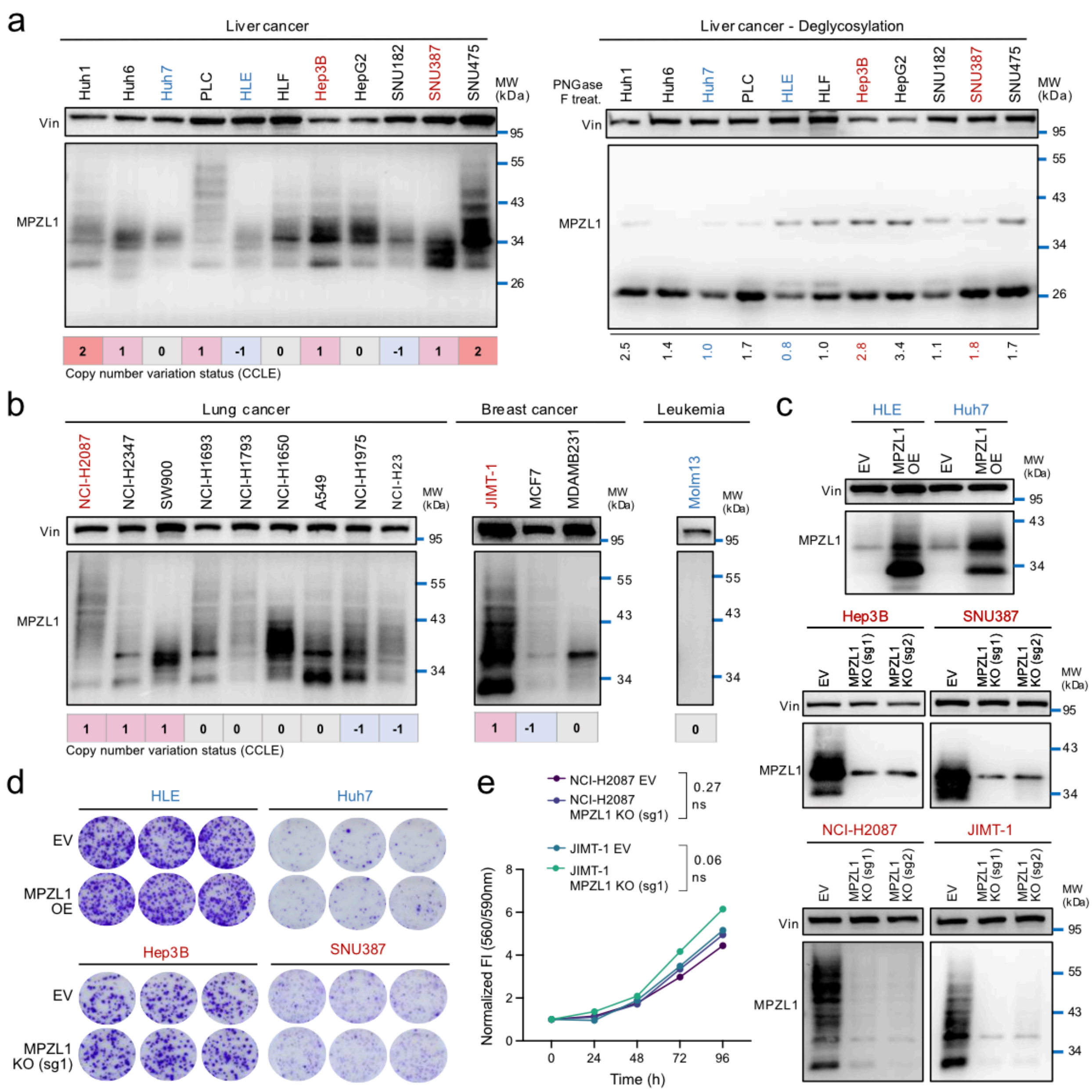

Supplementary Figure 3 | MPZL1 expression in human cancer cell lines.

(a) MPZL1 protein expression in human liver cancer cell lines by immunoblotting of whole-cell lysates (WCL). Copy number variation (CNV) status was obtained from CCLE via cBioPortal and defined using GISTIC2.0 (amplification, gain, diploid, shallow deletion). Right, MPZL1 deglycosylation using PNGase F followed by immunoblotting. Vinculin was used as loading control. Band intensities were quantified and normalized to vinculin. Quantification of MPZL1 protein expression was based on samples derived from gels/blots that were processed in parallel. Immunoblots are representative of at least two independent experiments with similar results. **(b)** MPZL1 expression in breast and lung cancer cell lines by immunoblotting. Molm13 leukemia cells served as negative control. Cell lines were categorized as MPZL1^{high} or MPZL1^{low} based on expression and CNV status. **(c–e)** Generation and characterization of isogenic cell lines. **(c)** MPZL1 expression by immunoblotting in MPZL1 overexpression (OE) and knockout (KO) models (two different CRISPR/CAS guides are used (sg1 and sg2)). EV = empty vector control. Immunoblots are representative of two independent experiments with

similar results. **(d)** Colony formation assays following 2-week culture of 1 × 10^3 cells per well. Data are representative of two independent experiments with similar results. **(e)** Cell proliferation assays measured by CellTiter-Blue over time. Data are shown as mean ± SD of three technical replicates. Data are representative of two independent experiments with similar results. Statistical analysis used paired t-test where indicated (p-values are indicated. ns, not significant). Source data and uncropped blots are provided as a Source Data file.

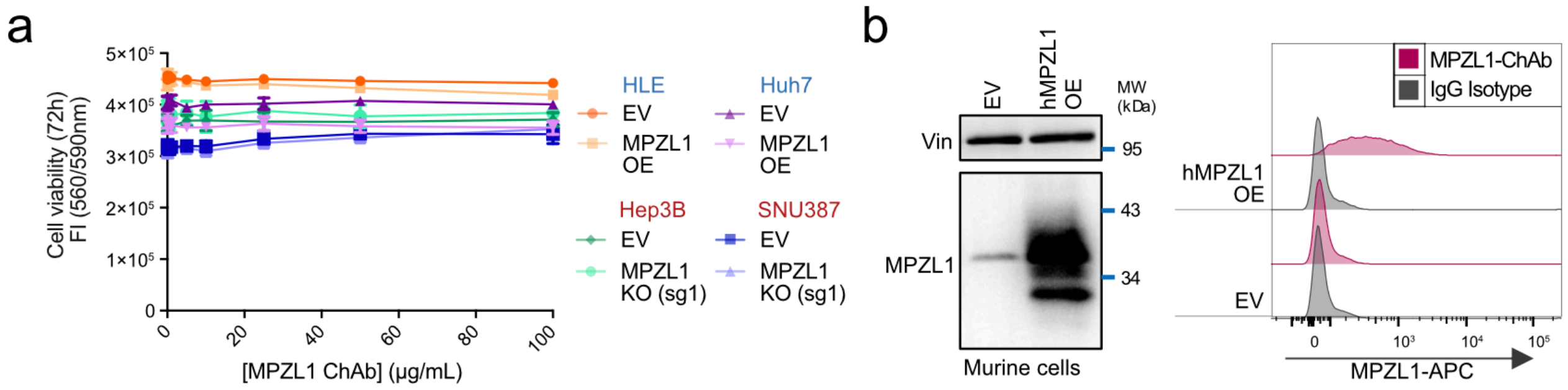

Supplementary Figure 4 | Characterization of MPZL1-ChAb binding and specificity.
(a) Dose-dependent cytotoxicity of MPZL1-ChAb in isogenic liver cancer cell lines (See Fig. S3). Cells transduced with empty vector (EV) or human MPZL1 (OE) or MPZL1 CRISPR/Cas9 constructs (MPZL1 KO sg1) were treated with 0.05–100 µg/mL antibody for 72 h. Cell viability was measured using CellTiter-Blue assay. Data are shown as mean ± SD of three technical replicates. **(b)** Specificity of MPZL1-ChAb binding in murine HTVI-derived liver cancer cells (Kras^{G12D}; p53$^{-/-}$). Cells were transduced with empty vector (EV) or human MPZL1 (OE). Left, immunoblot confirming human-specific MPZL1 detection. Right, flow cytometry histograms showing MPZL1-ChAb binding compared to isotype control. Data are representative of at least two independent experiments with similar results. Source data and uncropped blots are provided as a Source Data file.

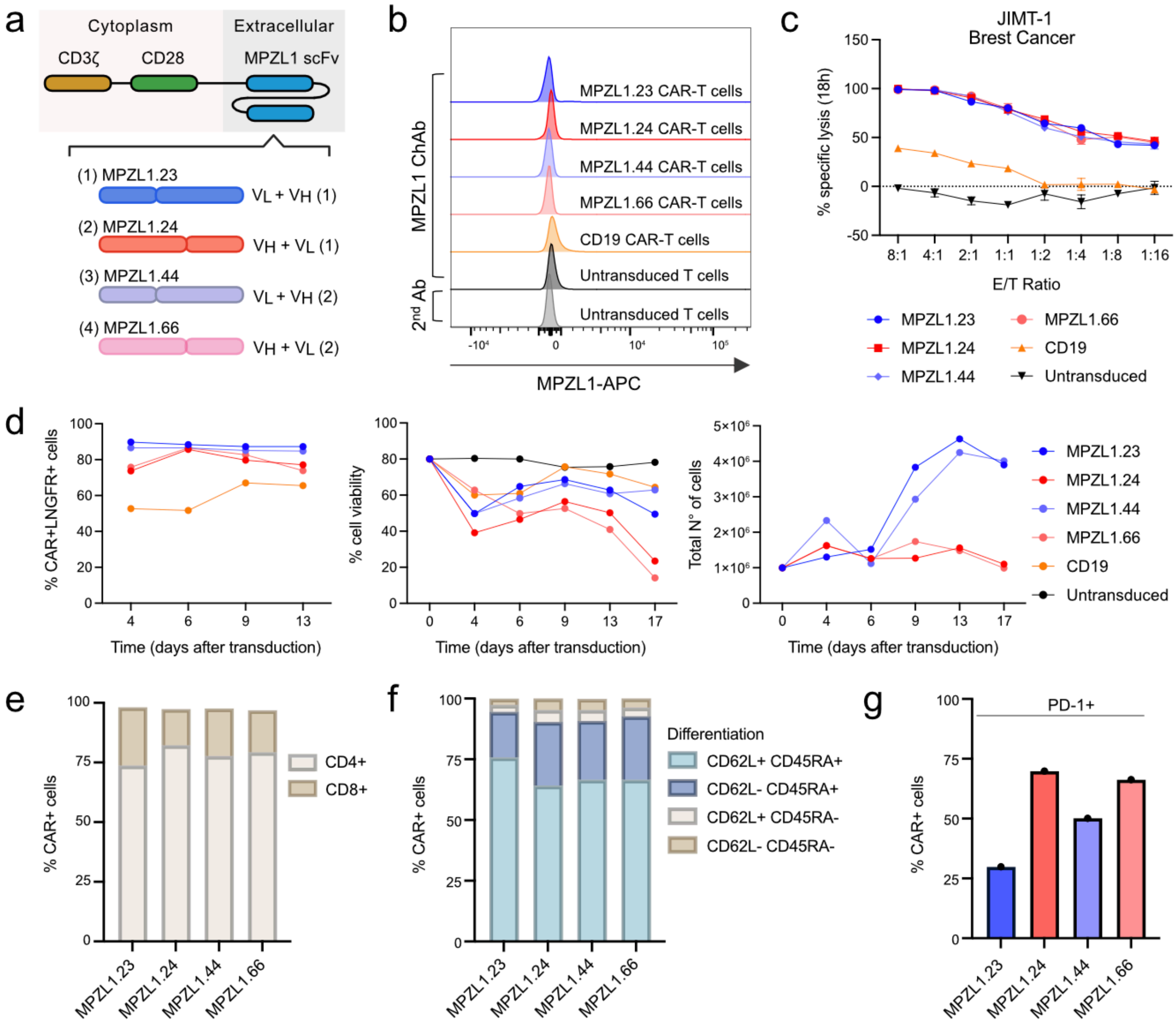

Supplementary Figure 5 | MPZL1 CAR constructs and in vitro characterization

(a) Schematic of MPZL1 CAR constructs with VL–VH/ VH–VL orientation. Note that MPZL1.24 and MPZL1.66 contain one additional amino acid at the C-terminus compared with MPZL1.23 and MPZL1.44, reflecting the inclusion of terminal nucleotides of the MPZL1-ChAb sequence whose coding status could not be unambiguously resolved during sequence verification. **(b,c)** CAR-T cell characterization six days post-transduction (n=1). **(b)** Flow cytometry analysis of MPZL1-ChAb binding to CAR-T cells. **(c)** Bioluminescence-based cytotoxicity against JIMT-1 (MPZL1^{high}) cells at decreasing effector-to-target ratios over 18 h. Data are shown as mean ± SD of three technical replicates. **(d)** CAR-T expansion and viability over time measured by flow cytometry and cell counting.**(e–g)** Flow cytometry phenotypic profiling 11 days post-transduction (n=1). **(e)** CD4/CD8 distribution. **(f)** Differentiation states based on CD62L and CD45RA. **(g)** PD-1 expression.

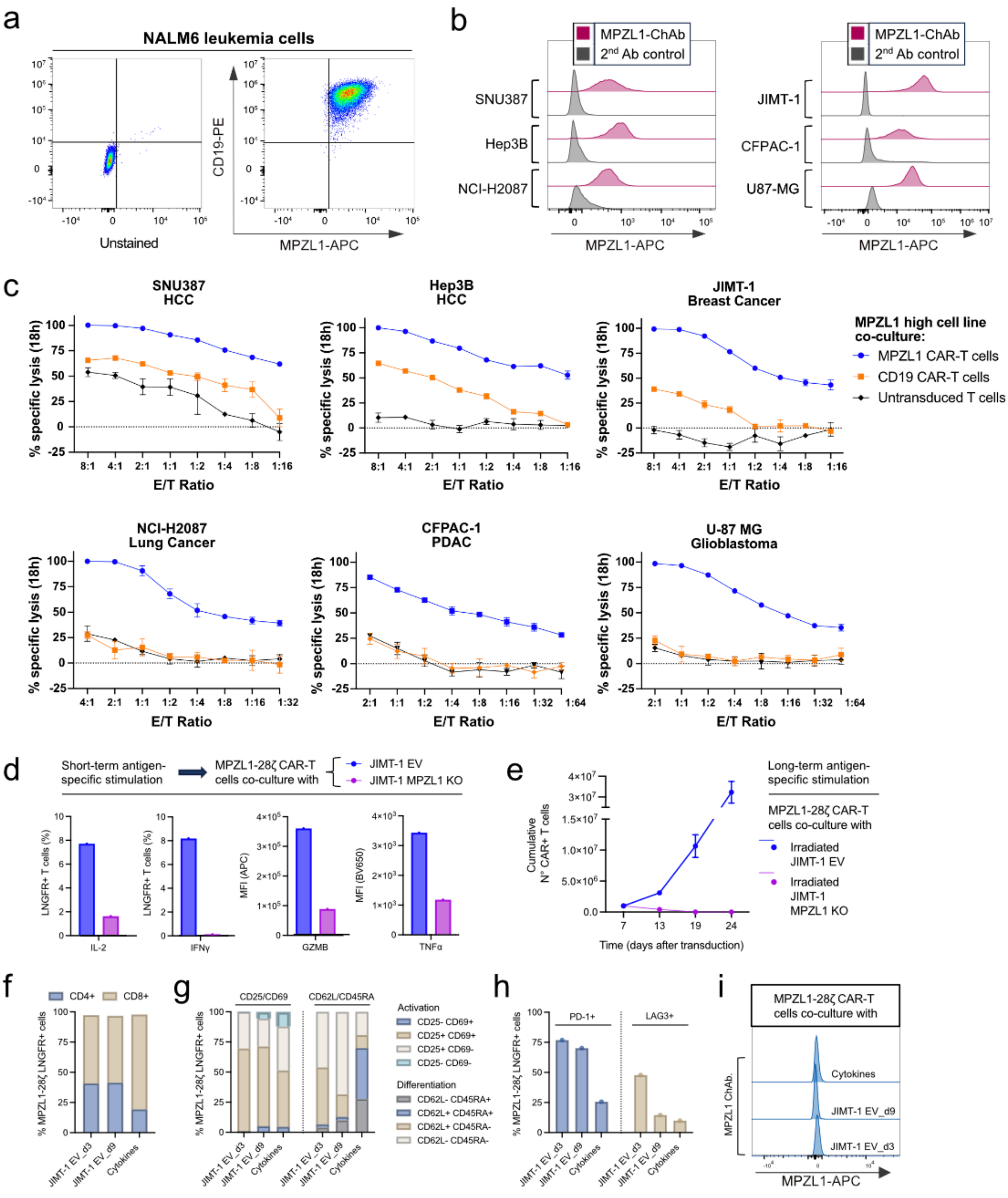

Supplementary Figure 6 | MPZL1 CAR-T cytotoxicity and functional profiling across tumor models.

(a) Flow cytometry analysis of NALM6 cells (MPZL1^{high}; CD19^{high}) stained for MPZL1 and CD19. Data are representative of at least two independent experiments with similar results. **(b)** Binding of MPZL1-ChAb across multiple tumor cell lines. n=2 for SNU387, Hep3B, JIMT-1. n=1 for NCI-H2087,CFPAC-1, U-87MG. **(c)** Bioluminescence-based cytotoxicity assays of MPZL1 CAR-T, CD19 CAR-T, and untransduced T cells against MPZL1^{high} cancer cell lines. Data are shown as mean ± SD of three technical replicates. n=2 for SNU387, Hep3B, JIMT-1. n=1 for NCI-H2087,CFPAC-1, U-87 MG. **(d–i)** Functional and phenotypic analyses of MPZL1 CAR-T using co-culture with JIMT-1 EV or MPZL1 KO target cells (n=1). **(d)**

Intracellular cytokine production (IL-2, IFNγ, Granzyme B, TNFα). **(e)** Antigen-dependent proliferation upon repeated stimulation cycles. Data are shown as mean ± SD of three technical replicates. **(f)** Flow cytometry analysis of CD4/CD8 distribution **(g)** Flow cytometry analysis of activation and differentiation states based on CD25, CD69, CD62L, and CD45RA. **(h)** Flow cytometry analysis of exhaustion markers PD-1 and LAG3. **(i)** Flow cytometry analysis of MPZL1-ChAb binding after repeated stimulation. Source data are provided as a Source Data file.

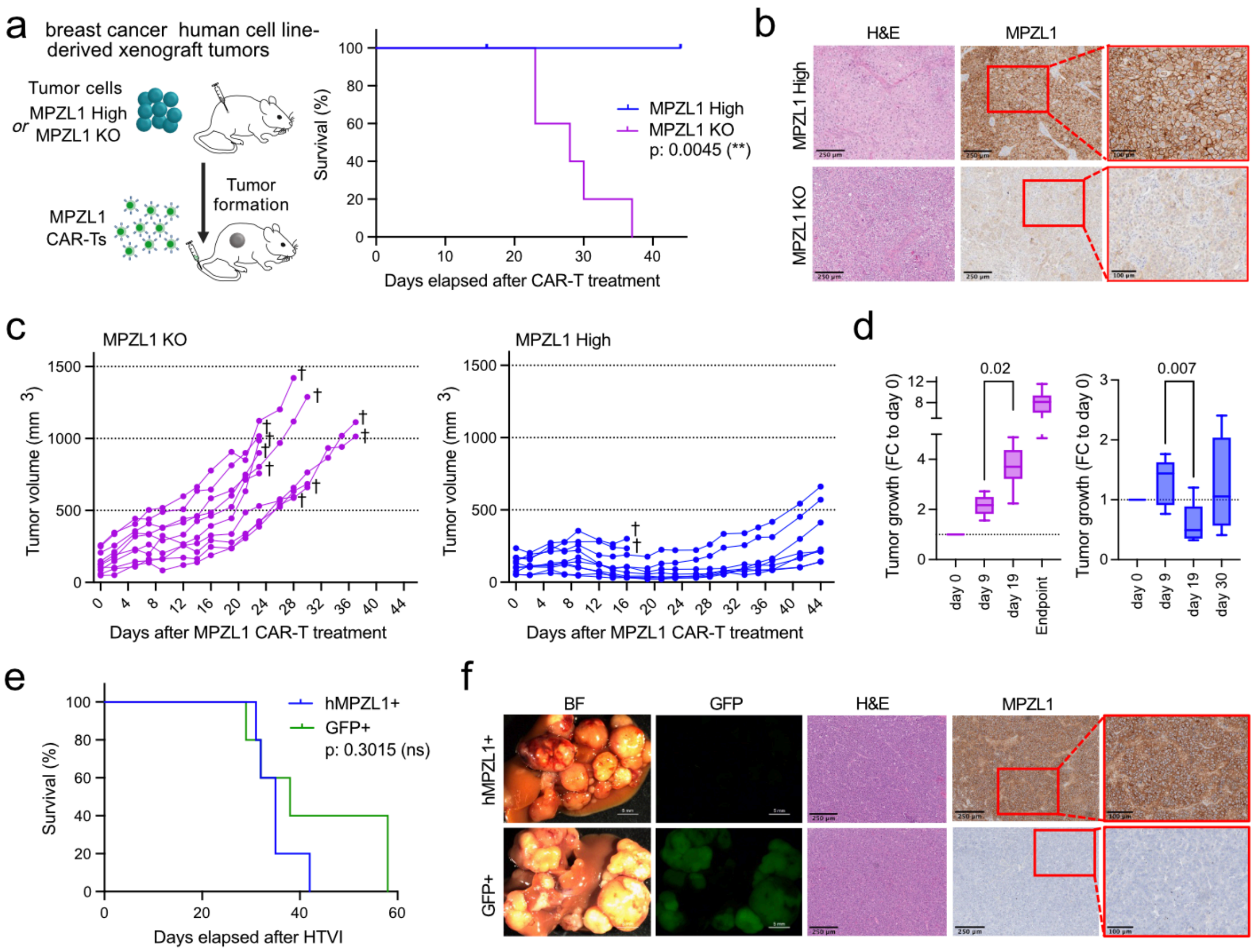

Supplementary Figure 7 | In vivo efficacy of MPZL1 CAR-T cells in human breast cancer xenograft models and characterization of autochthonous HCC models.

(a–d) Xenograft model. **(a)** Overall survival analysis of NSG mice bearing human breast cancer cell JIMT-1 EV or MPZL1 KO tumors treated with MPZL1 CAR-T cells (n = 5 per group). Statistical differences among groups were assessed using a two-sided Log-rank (Mantel-Cox) testing. p-value are indicated. **(b)** Representative H&E and MPZL1 IHC staining of tumor sections. Scale bars, 250 μm and 100 μm respectively. **(c)** Tumor growth kinetics following treatment (day 0 = treatment). Individual tumor sizes are plotted with n=10 independent tumors per group measured along time. Cross indicates termination of experiment. (**d**) Quantification of tumor growth expressed as fold change from baseline. Each graph represents a different group (left, MPZL1 KO; right, MPZL1^{high}), with n= 10 independent tumors per group measured along time. Within each group, statistical differences among time points were assessed using one-way ANOVA followed by Tukey's multiple-comparisons test (two-sided test). Adjusted p-value are indicated. Box plots show the median, the 25th and 75th percentiles, and whiskers extending to the minimum and maximum values. **(e–f)** Autochthonous HCC model without treatment. Liver tumors were induced by hydrodynamic delivery of c-MYC and p53 knockout together with either MPZL1 or GFP (MycOE;p53$^{-/-}$;MPZL1 or GFP; n = 5 per group). **(e)** Overall survival analysis. Statistical differences among groups (n= 5 per group) were assessed using a two-sided Log-rank (Mantel-Cox) testing. p-value are indicated. ns = not significant. **(f)** Representative liver imaging and histology of MPZL1- and GFP-expressing tumors. Scale bars, 5mm for stereomicroscopic images, and 250 μm and 100 μm for IHC images. Source data are provided as a Source Data file.

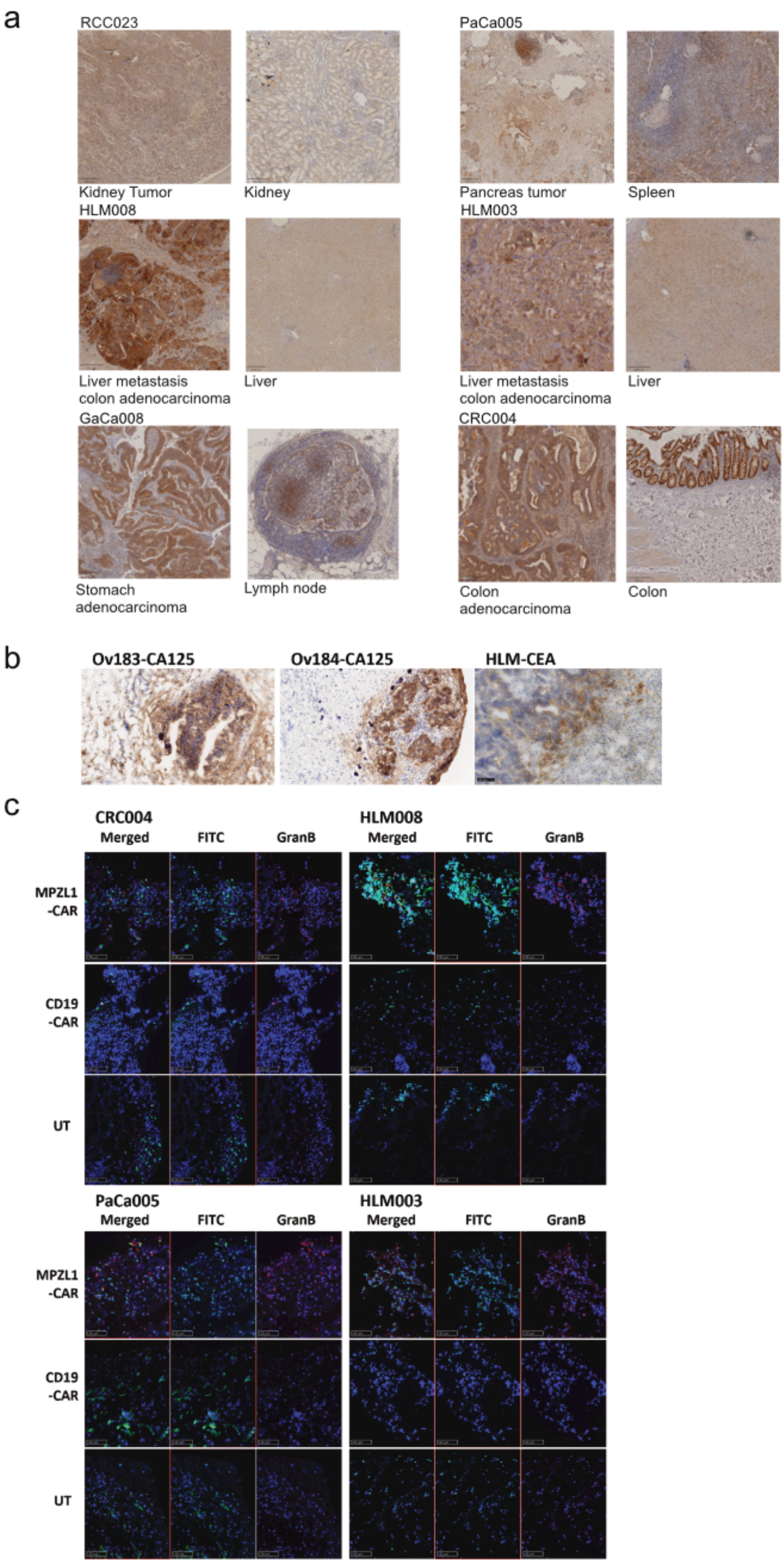

Supplementary Figure 8 | Characterization of human tissue explant samples.
(a) Representative MPZL1 IHC staining in tumor and matched normal tissues from patient-derived explants. scale bar: 100 μm **(b)** Representative IHC for CA125 and CEA in ovarian cancer and liver metastasis explants treated with corresponding CAR-CLT. **(c)** Representative

immunofluorescence staining of granzyme B (GranB) in explants treated with MPZL1 CAR-CLT, CD19 CAR-CLT, or untransduced T cells (n=4). Nuclei were counterstained with DAPI. CLT were labeled with FITC, GranB with Cy5. scale bar: 100 μm. Sample IDs correspond to Fig. 6.

Supplementary Tables

<u>Supplementary Table S1</u>. CSP-coding genes amplified in more than 30% of pan-cancer patient samples (N=90 hits; n=2703 samples).

CSP gene	Samples amplified (%)	Chromosome	CSP gene	Samples amplified (%)	Chromosome
EFNA1	33,67	1	*FCRL2*	32,37	1
EFNA4	33,63	1	*CD1B*	32,33	1
ADAM15	33,59	1	*SV2A*	32,33	1
GBA1	33,56	1	*PRRC2C*	32,33	1
THBS3	33,56	1	*ATP1B1*	32,26	1
ENTREP3	33,56	1	*FCRL5*	32,22	1
PBXIP1	33,52	1	*CD1D*	32,19	1
ADAMTSL4	33,33	1	*SELL*	32,19	1
MPZ	33,33	1	*SELP*	32,19	1
B4GALT3	33,30	1	*F5*	32,19	1
FCGR2A	33,22	1	*SLC19A2*	32,19	1
F11R	33,19	1	*SUCO*	32,11	1
FCGR3A	33,07	1	*TNFSF18*	32,08	1
FCGR3B	33,04	1	*KIRREL1*	32,04	1
LY9	33,04	1	*FCGR1A*	31,85	1
CD244	33,04	1	*TOR1AIP2*	31,52	1
SLAMF7	33,04	1	*TOR1AIP1*	31,52	1
NPR1	33,04	1	*QSOX1*	31,45	1
NCSTN	33,04	1	*TOR3A*	31,26	1
CTSK	33,04	1	*MR1*	31,22	1
SLAMF1	33,00	1	*FAM20B*	31,22	1
IGSF8	33,00	1	*ANGPTL1*	31,15	1
CD48	33,00	1	*LAMC1*	31,08	1
CD84	33,00	1	*COLGALT2*	31,08	1
OLFML2B	33,00	1	*LAMC2*	31,04	1
CGN	33,00	1	*EDEM3*	30,93	1
SLAMF6	33,00	1	*HNRNPU*	30,67	1
FCGR2B	33,00	1	*NFASC*	30,56	1
SSR2	33,00	1	*PTGS2*	30,45	1
GLMP	32,96	1	*HMCN1*	30,37	1
DDR2	32,93	1	*PRELP*	30,11	1

SEMA4A	32,93	1	*ADORA1*	30,04	1
LMNA	32,93	1	*ST3GAL1*	35,15	8
TAGLN2	32,93	1	*TNFRSF11B*	35,04	8
CERS2	32,93	1	*COL14A1*	34,85	8
FCRL6	32,85	1	*LRP12*	34,15	8
FLG2	32,70	1	*ANGPT1*	34,07	8
PEAR1	32,63	1	*TRHR*	33,89	8
CREG1	32,59	1	*SLC45A4*	33,89	8
CADM3	32,59	1	*LY6K*	33,81	8
BCAN	32,56	1	*PKHD1L1*	33,70	8
NTRK1	32,56	1	*EEF1D*	33,56	8
CD1C	32,48	1	*CPQ*	33,04	8
MPZL1	32,45	1	*DPY19L4*	32,89	8
FCRL3	32,41	1	*CDH17*	32,45	8

Supplementary Table S2. Chr1q-amplified CSP-coding genes in HCC (N=77 hits, n=370 samples).

CSP gene	Samples amplified (%)	CSP gene	Samples amplified (%)	CSP genes	Samples amplified (%)
ADAM15	76.49	*CD244*	74.32	*F5*	73.51
EFNA1	76.22	*SLAMF7*	74.32	*SLC19A2*	73.51
EFNA4	76.22	*CREG1*	74.32	*ADAMTSL4*	73.24
PBXIP1	76.22	*FCRL5*	74.32	*FLG2*	73.24
GBA1	75.95	*SUCO*	74.32	*SELP*	73.24
THBS3	75.95	*SLAMF1*	74.05	*ADORA1*	73.24
ENTREP3	75.95	*CD48*	74.05	*CTSK*	72.97
NPR1	75.14	*CD84*	74.05	*CERS2*	72.97
SSR2	75.14	*TAGLN2*	74.05	*PRELP*	72.97
GLMP	75.14	*FCRL6*	74.05	*EDEM3*	72.43
PEAR1	75.14	*FCRL3*	74.05	*HNRNPU*	72.43
NTRK1	75.14	*PRRC2C*	74.05	*TOR3A*	72.16
MPZ	74.86	*ATP1B1*	74.05	*FAM20B*	72.16
FCGR2A	74.86	*TNFSF18*	74.05	*ANGPTL1*	72.16
FCGR3A	74.86	*NCSTN*	73.78	*HMCN1*	72.16
FCGR3B	74.86	*IGSF8*	73.78	*TOR1AIP2*	71.89

FCGR2B	74.86	*SLAMF6*	73.78	*TOR1AIP1*	71.89
SEMA4A	74.86	*CADM3*	73.78	*QSOX1*	71.89
LMNA	74.86	*CD1C*	73.78	*LAMC1*	71.89
BCAN	74.86	*FCRL2*	73.78	*COLGALT2*	71.89
B4GALT3	74.59	*CD1B*	73.78	*LAMC2*	71.89
OLFML2B	74.59	*CD1D*	73.78	*MR1*	71.62
DDR2	74.59	*KIRREL1*	73.78	*PTGS2*	71.62
MPZL1	74.59	*NFASC*	73.78	*SV2A*	68.65
F11R	74.32	*CGN*	73.51	*FCGR1A*	64.05
LY9	74.32	*SELL*	73.51		

Supplementary Table S3. Chr1q-amplified CSP-coding genes differentially expressed between cancer and healthy liver tissues (N=19 hits; n=369 tumor and 160 normal samples). mRNA differential expression is considered as $|Log_2FC|>1$.

Gene	Log_2FC	Gene	Log_2FC	Gene	Log_2FC
GBA	2.452	*PBXIP1*	1.440	*NCSTN*	1.196
LAMC1	2.038	*ATP1B1*	1.370	*F5*	1.152
LMNA	1.913	*FAM189B*	1.313	*FAM20B*	1.114
GLMP	1.742	*ADAM15*	1.286	*SUCO*	1.113
TAGLN2	1.716	*EFNA4*	1.255	*IGSF8*	1.056
SSR2	1.633	*MPZL1*	1.247	*TOR3A*	1.026
OLFML2B	1.568				

Supplementary Table S4. Candidate CSPs for a targeted therapy (N=6 hits).

Gene	Full name	Protein cellular localization
ATP1B1	Na+/K+ transporting ATPase subunit beta-1	Cell membrane
FAM189B	Endosomal transmembrane epsin interactor 3	Membrane (*ENTREP3*)
EFNA4	Ephrin-A4	Cell membrane
MPZL1	Myelin protein zero-like 1	Membrane / Cell surface
IGSF8	Immunoglobulin superfamily member 8	Cell membrane
ADAM15	Disintegrin and metalloproteinase domain-containing protein 15	Plasma membrane / Endomembrane system

<u>Supplementary Table S5</u>. Primary Antibodies used for immunoblotting.

Target	Clone	Species	Manufacturer	Catalog	Dilution
List of Primary Antibodies used for Immunoblotting					
MPZL1 (PZR-XP®)	D17B10	Rabbit	Cell Signaling Technology	#9893	1:1000 (5% Milk)
Na/K-ATPase	Polyclonal	Rabbit	Cell Signaling Technology	#3010	1:1000 (5% Milk)
β-Tubulin	TUB 2.1	Mouse	Santa Cruz Biotechnology	#sc-58886	1:300 (5% Milk)
Vin	hVIN-1	Mouse	Sigma-Aldrich	#V9131	1:5000 (5% Milk)
β-Actin-HRP	AC-15	Mouse	Sigma-Aldrich	#3854	1:5000 (5% Milk)

<u>Supplementary Table S6</u>. Secondary Antibodies used for immunoblotting.

Target	Clone	Manufacturer	Catalog	Dilution
List of Secondary Antibodies used for Immunoblotting				
Goat-HRP-Anti-mouse IgG	Polyclonal	Jackson ImmunoResearch	#115-035-146	1:5000 (5% Milk)
Goat-HRP-Anti-rabbit IgG	Polyclonal	Jackson ImmunoResearch	#111-035-144	1:5000 (5% Milk)

<u>Supplementary Table S7</u>. Antibodies used for immunohistochemistry.

Target	Species	Manufacturer	Catalog	Dilution
List of Primary Antibodies used for Immunohistochemistry				
MPZL1 (PZR-XP®, clone D17B10)	Rabbit	Cell Signaling Technology	#9893	1:60
c-Myc	Rabbit	Abcam	#ab32072	5 µg/mL
GFP (clone D5.1)	Rabbit	Cell Signaling Technology	#2956	1:500
CD3	Rabbit	Epredia	#RM-9107-S1	1:100
LNGFR (CD271)	Mouse	BD Pharmingen	#557194	1:100

Supplementary Table S8. Human TMAs used for MPZL1 IHC.

Tissue entity	N° patients	Description	Reference	Source
TMAs with samples of malignant origin				
HCC	144	—	—	NCT Tissue Bank
TNBC	156	2 cores / patient	Nr. 021/1-4	NCT Tissue Bank
BRCA	292	2 cores / patient	Nr. 023/1-3	NCT Tissue Bank
NSCLC	1441	2 cores / patient	Nr. 08/1-29	NCT Tissue Bank
SCC	43	1 core / patient	Nr. 19/1	NCT Tissue Bank
PDAC	194	2 cores / patient	1-4	NCT Tissue Bank
CRC	65	2-3 cores / patient	Nr. 026/1a-f	NCT Tissue Bank
TMA with samples of healthy origin				
Various	30	3 patients / tissue<br>2 cores / patient	Nr. 0130/1-2	NCT Tissue Bank

Supplementary Table S9. Sequence of the variable regions of the MPZL1 hybridoma for nSeq FR1 region.

Antibody chain	nSeq FR1 region
Kappa light chain	gacatccagatgactcagtctccagcctccctatctgtatctgtgggagaaactgtcaccatcacatgtcgagcaagt
Heavy chain	caggttactctgaaagagtctggccctgggatattgcagccctcccagaccctcagtctgacttgttctttctct

Supplementary Table S10. Sequence of the variable regions of the MPZL1 hybridoma for nSeq CDR1 region and nSeq FR2 region.

Antibody chain	nSeq CDR1 region	nSeq FR2 region
Kappa light chain	gagaatatttacagtaat	ttagcatggtatcagcagaaacagggaaaatctcctcagctcctggtctat
Heavy chain	gggttttcactgagcacttttggtatgggt	gtaggctggattcgtcagccttcagggaagggtctggagtggctggcacac

Supplementary Table S11. Sequence of the variable regions of the MPZL1 hybridoma nSeq CDR2 region and nSeq FR3 region.

Antibody chain	nSeq CDR2 region	nSeq FR3 region
Kappa light chain	gctgcaaca	aacttagcagatggtgtgccatcaaggttcagtggcagtggatcaggcacacagtattccctcaagatcaacagcctgcagtctgaagattttgggagttattac

Heavy chain	atttggtgggatgatgataag	tactataacccagccctgaagagtcggctcacaatctccaaggatacctccaaaaaccaggtattcctcaagatcgccaatgtggacactgcagatactgccacatactac

<u>Supplementary Table S12.</u> Sequence of the variable regions of the MPZL1 hybridoma nSeq CDR3 region and nSeq FR4 region.

**Antibody chain**	**nSeq CDR3 region**	**nSeq FR4 region**
Kappa light chain	tgtcaacatttttggggtactcctcccacgttc	ggagggggggaccaagctggaaataaaac
Heavy chain	tgtgctcgaatagcccctattactacggtagtagcctactggtacttcgatgtctgg	ggcacagggaccacggtcaccgtctcctcag

Uncropped immunoblots for Supplementary Figures:

Fig. S3:

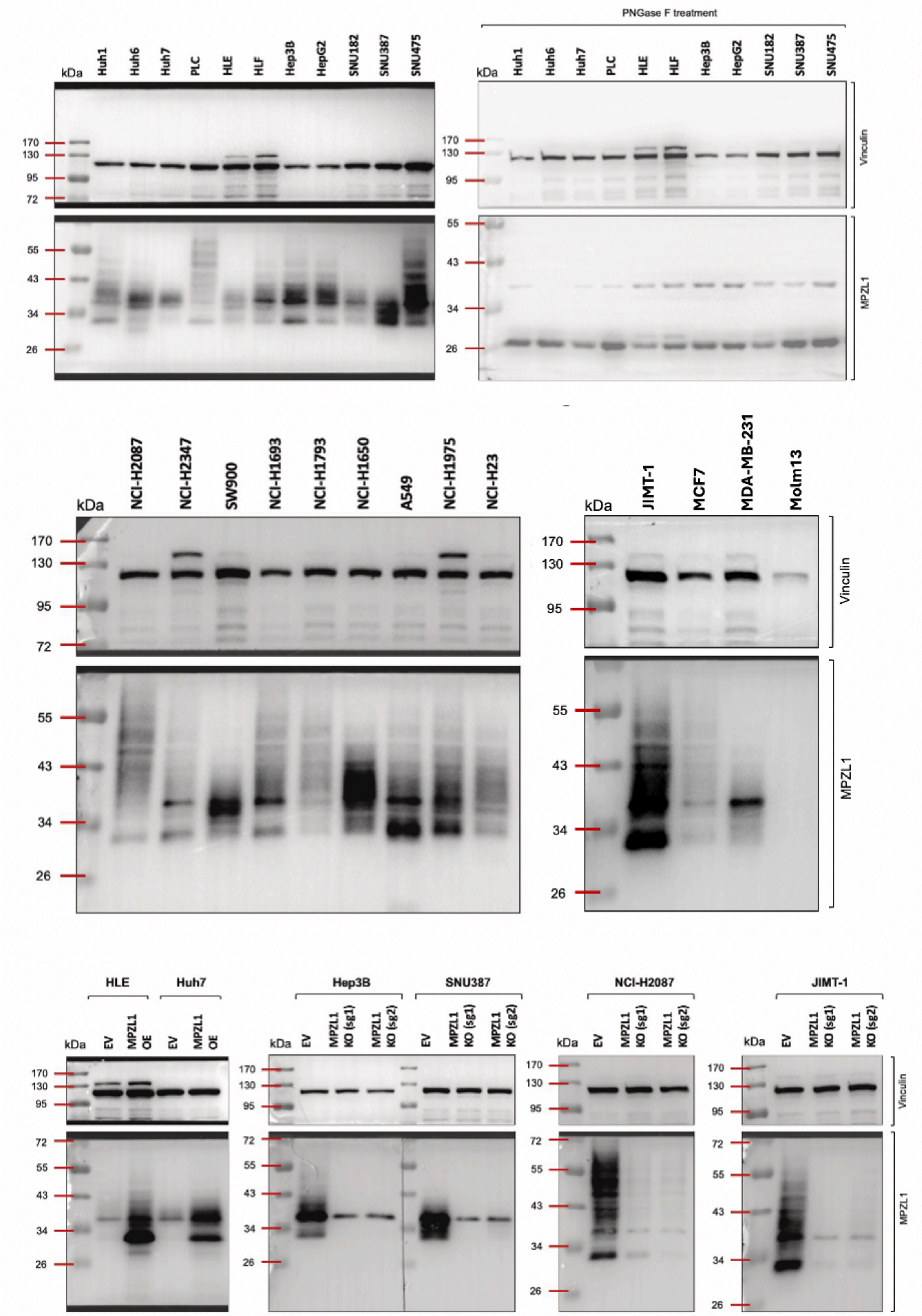

Fig. S4:

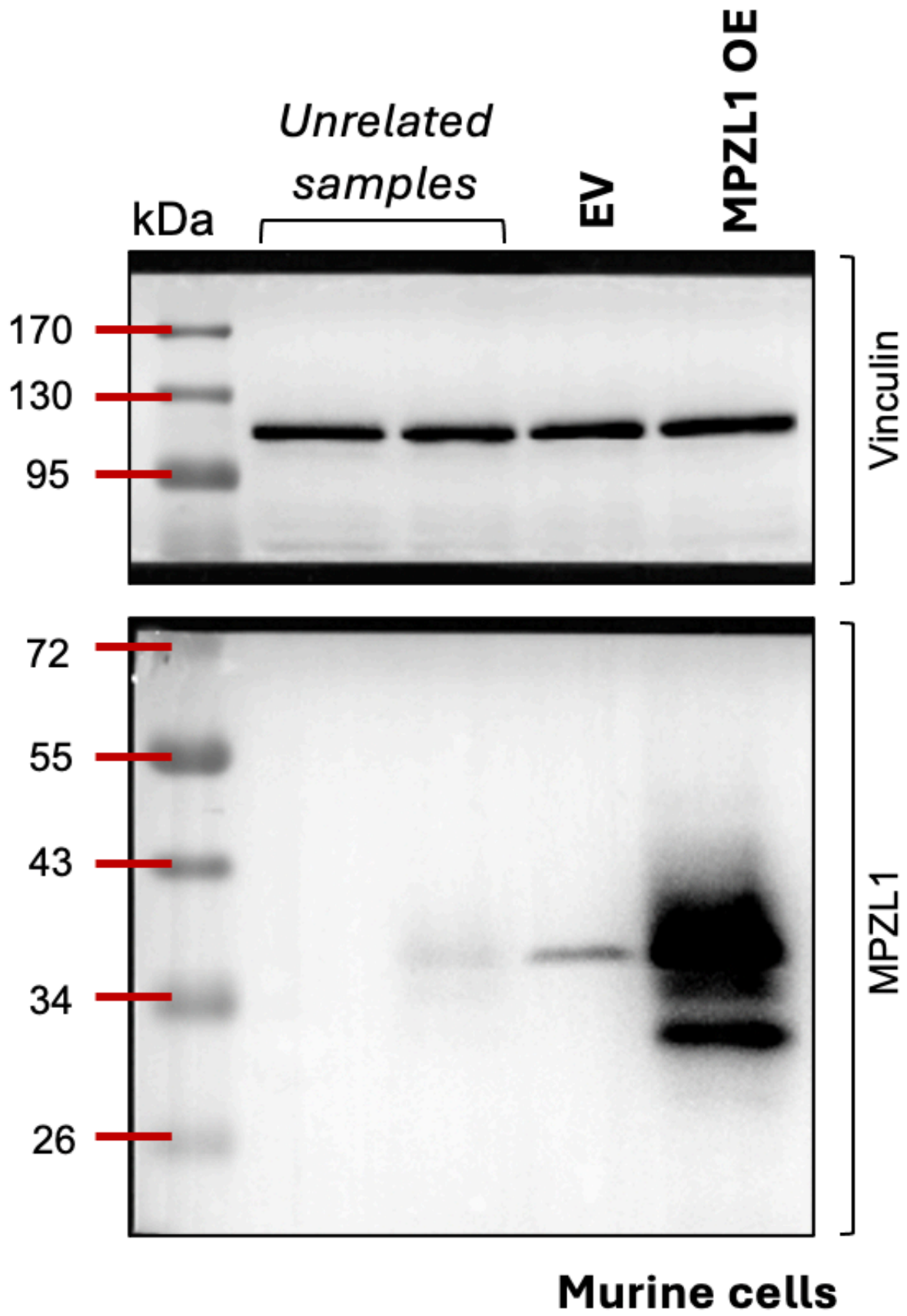

Unrelated samples
EV
MPZL1 OE
kDa
170
130
95
72
55
43
34
26
Vinculin
MPZL1
Murine cells

Representative Gating Strategy for FACS analyses.

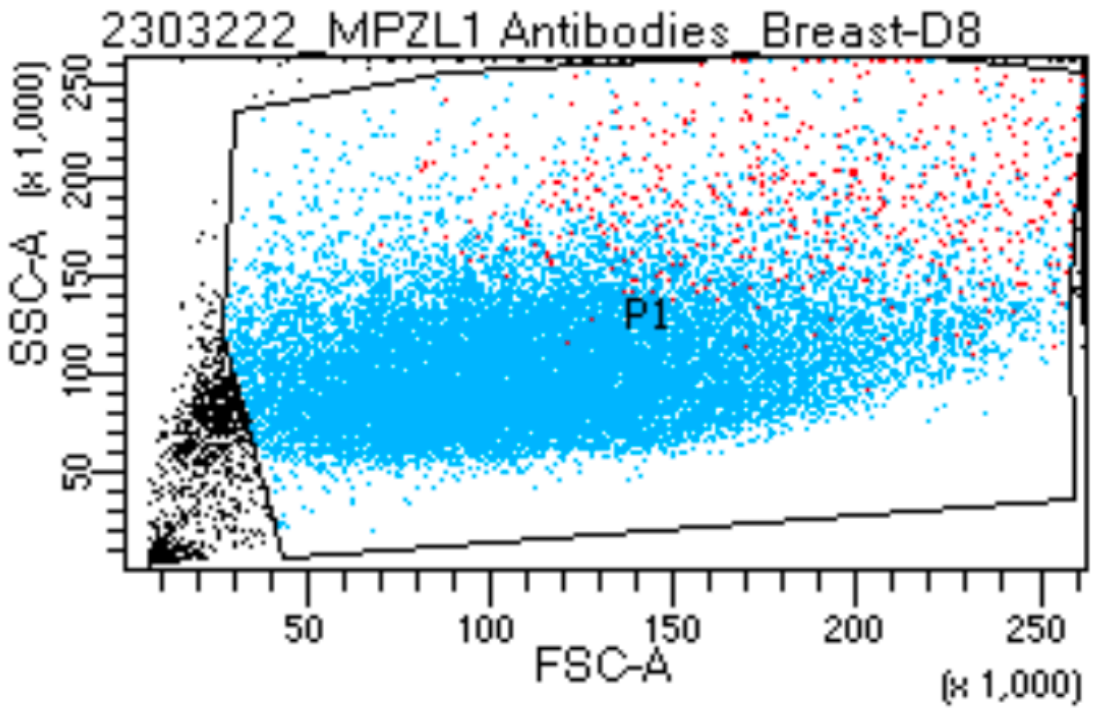

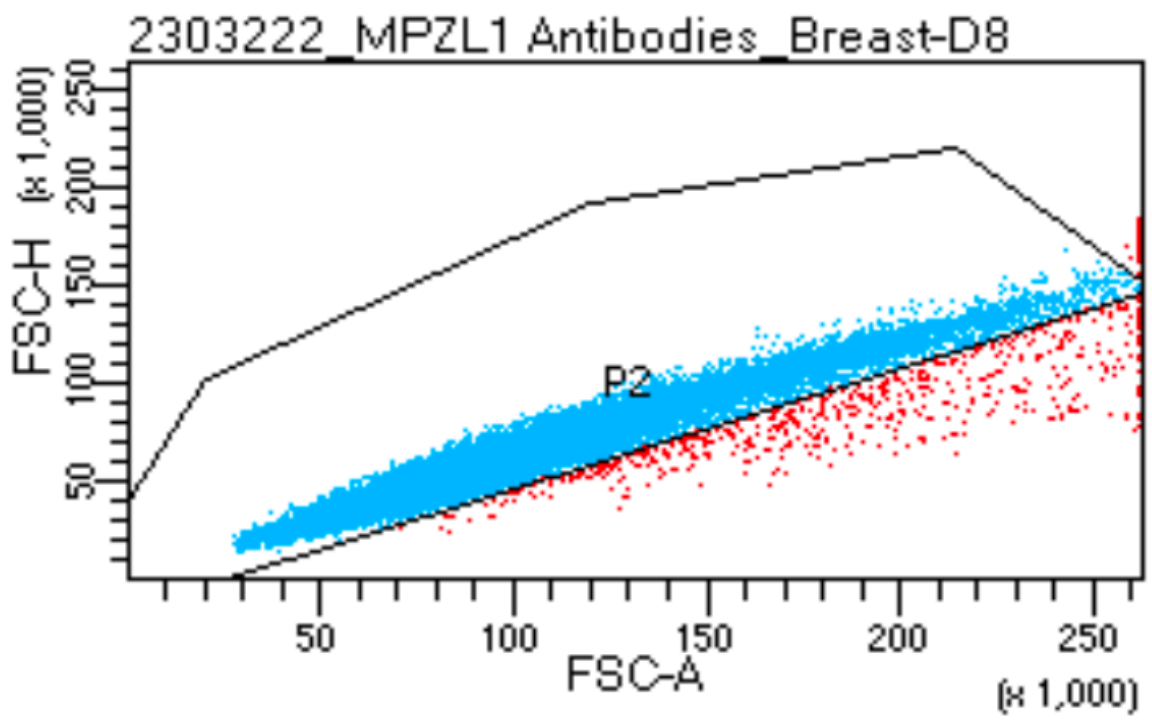

MPZL1^{High} cell line

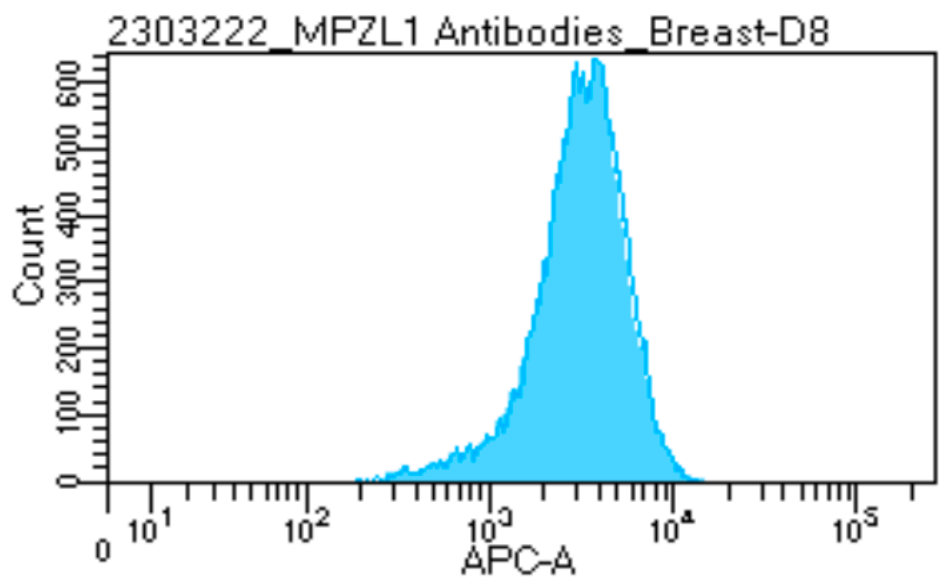

MPZL1 KO cell line

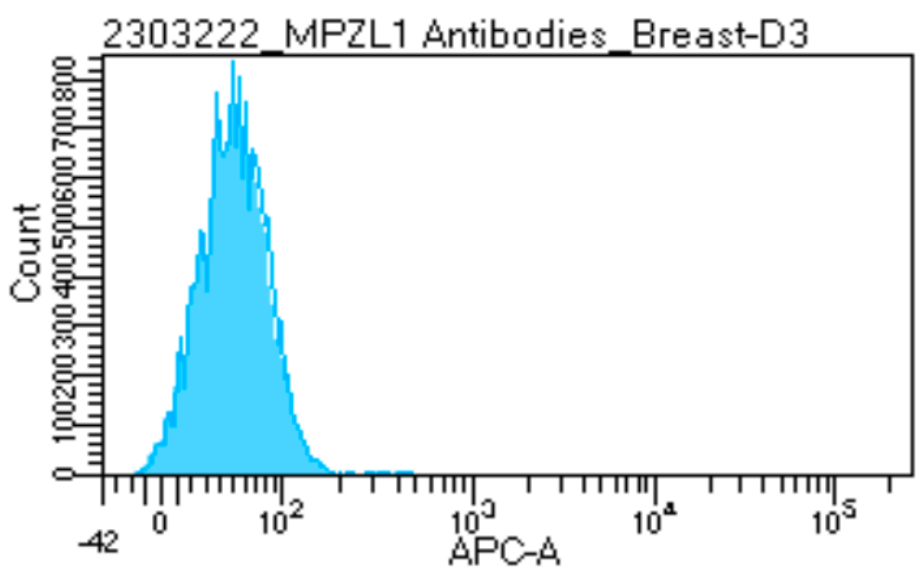

Supplement: Supplementary file 1 [file 41467_2026_77465_MOESM1_ESM.pdf]
