# Supplementary material for: Cancer-driver-agnostic targeting of amplified surface proteins identifies MPZL1 for selective CAR-T cell therapy
Source: Nat Commun. 2026 Sep 10;17:9695. doi: 10.1038/s41467-026-77465-5 (PMC13562587; doi:10.1038/s41467-026-77465-5)
Supplement: Supplementary file 2 [file 41467_2026_77465_MOESM2_ESM.pdf]

nature portfolio

Corresponding author(s): Breinig Marco, Darjus Tschaharganeh

Last updated by author(s): Jul 1, 2026

Reporting Summary

Nature Portfolio wishes to improve the reproducibility of the work that we publish. This form provides structure for consistency and transparency in reporting. For further information on Nature Portfolio policies, see our Editorial Policies and the Editorial Policy Checklist.

Statistics

For all statistical analyses, confirm that the following items are present in the figure legend, table legend, main text, or Methods section.

n/a	Confirmed	
☐	☒	The exact sample size (*n*) for each experimental group/condition, given as a discrete number and unit of measurement
☐	☒	A statement on whether measurements were taken from distinct samples or whether the same sample was measured repeatedly
☐	☒	The statistical test(s) used AND whether they are one- or two-sided<br>*Only common tests should be described solely by name; describe more complex techniques in the Methods section.*
☒	☐	A description of all covariates tested
☒	☐	A description of any assumptions or corrections, such as tests of normality and adjustment for multiple comparisons
☐	☒	A full description of the statistical parameters including central tendency (e.g. means) or other basic estimates (e.g. regression coefficient) AND variation (e.g. standard deviation) or associated estimates of uncertainty (e.g. confidence intervals)
☐	☒	For null hypothesis testing, the test statistic (e.g. *F*, *t*, *r*) with confidence intervals, effect sizes, degrees of freedom and *P* value noted<br>*Give P values as exact values whenever suitable.*
☒	☐	For Bayesian analysis, information on the choice of priors and Markov chain Monte Carlo settings
☒	☐	For hierarchical and complex designs, identification of the appropriate level for tests and full reporting of outcomes
☒	☐	Estimates of effect sizes (e.g. Cohen's *d*, Pearson's *r*), indicating how they were calculated

Our web collection on statistics for biologists contains articles on many of the points above.

Software and code

Policy information about availability of computer code

Data collection	DepMap, Gepia, UniProt, HumanProteinAtlas, cBioportal, CSPA, BioMart
Data analysis	Gepia2. GISTIC2, DepMap, QuPath, FiJi/ImageJ, FlowJo v10

For manuscripts utilizing custom algorithms or software that are central to the research but not yet described in published literature, software must be made available to editors and reviewers. We strongly encourage code deposition in a community repository (e.g. GitHub). See the Nature Portfolio guidelines for submitting code & software for further information.

Data

Policy information about availability of data

All manuscripts must include a data availability statement. This statement should provide the following information, where applicable:

- Accession codes, unique identifiers, or web links for publicly available datasets
- A description of any restrictions on data availability
- For clinical datasets or third party data, please ensure that the statement adheres to our policy

Source data are provided with this paper.

Research involving human participants, their data, or biological material

Policy information about studies with human participants or human data. See also policy information about sex, gender (identity/presentation), and sexual orientation and race, ethnicity and racism.

Reporting on sex and gender	Sex and gender were not explicitly considered in study design.
Reporting on race, ethnicity, or other socially relevant groupings	Race, ethnicity, or other socially relevant groupings were not explicitly considered in study design.
Population characteristics	Population characteristics were not explicitly considered in study design. We provide information related to sex and age in the Source Data File.
Recruitment	Buffy coats from anonymous healthy human donors were purchased from either the ZKT Tübingen or the IKTZ Heidelberg (in German, Zentrum für Klinische Transfusionsmedizin, and Institut für Klinische Transfusionsmedizin und Zelltherapie, respectively).
Ethics oversight	Institutional Ethical Review Board approval was obtained at local Ethical Committees of the Medical Universities of Heidelberg (S205-06), and of the University Medical Centre Mainz (2025-18147) in compliance with the Helsinki Declaration.

Note that full information on the approval of the study protocol must also be provided in the manuscript.

Field-specific reporting

Please select the one below that is the best fit for your research. If you are not sure, read the appropriate sections before making your selection.

☒ Life sciences ☐ Behavioural & social sciences ☐ Ecological, evolutionary & environmental sciences

For a reference copy of the document with all sections, see nature.com/documents/nr-reporting-summary-flat.pdf

Life sciences study design

All studies must disclose on these points even when the disclosure is negative.

Sample size	No statistical methods were used to pre-determine sample size. Sample sizes were estimated based on preliminary experiments, with an effort to achieve a minimum of n=5 mice per treatment group which proved to be sufficient to reproducibly observe a statistically significant difference.
Data exclusions	No data were excluded throughout the studies.
Replication	All in vitro and in vivo experiments were repeated in replicates and/or from different subjects in independent experiments. All attempts at replication were successful. Specific information is provided in the figure legends.<br>Additional characterization of different CAR T cell constructs related to analysis of CD4/CD8 distribution, CD62L and CD45RA and PD-1 expression was performed with n =1.
Randomization	Mice were randomly assigned into treatment groups. Buffy coats were obtained from anonymous donors.
Blinding	Mouse conditions were observed by an operator who was blinded to the treatment groups in addition to the main investigator who was not blind to group allocation. Analysis of data was not performed in blinded fashion. Data analysis are based on objectively measurable data (fluorescence intensity, tumor burden, cell count).

Reporting for specific materials, systems and methods

We require information from authors about some types of materials, experimental systems and methods used in many studies. Here, indicate whether each material, system or method listed is relevant to your study. If you are not sure if a list item applies to your research, read the appropriate section before selecting a response.

Materials & experimental systems

n/a	Involved in the study
☐	☒ Antibodies
☐	☒ Eukaryotic cell lines
☒	☐ Palaeontology and archaeology
☐	☒ Animals and other organisms
☒	☐ Clinical data
☒	☐ Dual use research of concern
☒	☐ Plants

Methods

n/a	Involved in the study
☒	☐ ChIP-seq
☐	☒ Flow cytometry
☒	☐ MRI-based neuroimaging

Antibodies

Antibodies used

Na/K-ATPase (Cell Signaling Technology, #3010), β-Tubulin (Santa Cruz Biotechnology, #sc-58886), Vimentin (Vin) (Sigma-Aldrich, #V9131), β-Actin-HRP (Sigma-Aldrich, #3854), Goat-HRP-Anti-mouse IgG (Jackson ImmunoResearch, #115-035-146), Goat-HRP-Anti-rabbit IgG (Jackson ImmunoResearch, #111-035-144), MPZL1 (PZR-XP®, clone D17B10) (Cell Signaling Technology, #9893), c-Myc (Abcam, #ab32072), GFP (clone D5.1) (Cell Signaling Technology, #2956), CD3 (Epredia, #RM-9107-S1), LNGFR (CD271) (BD Pharmingen, #557194), AF647® goat anti-mouse IgG F(ab')2 fragment specific (AffiniPure), APC Mouse Anti-Human/Mouse GZMB (BioLegend), PerCP Rat Anti-Human IL-2 (BioLegend), BUV737 Mouse Anti-Human IFNγ (BD Biosciences), BV650 Mouse Anti-Human TNFα (BD Biosciences), BUV395 CD4 (BD Biosciences), BV650 CD45RA (BD Biosciences), PE CD271 (LNGFR) (BD Biosciences), PE/Cyanine7 CD8a (Invitrogen), PerCP-eFluor 710-LAG3 (Invitrogen), BV510 CD25 (BioLegend), BV711 CD69 (BioLegend), APC/Cyanine7 PD-1 (BioLegend), BV421 CD62L (BioLegend). Additional information is provided in the methods section.

Validation

We validated MPZL1 antibodies by testing their detection in multiple isogenic MPZL1 wildtype and CRISPR/Cas9 knockout cell lines. All other antibodies are validated. Data are available on the manufacturer's website.

Eukaryotic cell lines

Policy information about cell lines and Sex and Gender in Research

Cell line source(s)

The human cell lines U-87 MG (ATCC HTB-14), CFPAC-1 (ATCC CRL-1918), NALM-6 (ATCC CRL-3273), HEK293 (ATCC CRL-1573), JIMT-1 (DSMZ ACC 589), MOLM-13 (DSMZ ACC 554), Hep3B (ATCC HB-8064), SNU-387 (ATCC CRL-2237), Huh7 (JCRB0403), HLE (ATCC CRL-5924), HuH-1 (JCRB0199), HuH-6 (JCRB0401), PLC/PRF/5 (ATCC CRL-8024), HLF (JCRB0405), HepG2 (ATCC HB-8065), SNU-475 (ATCC CRL-2236), NCI-H2087 (ATCC CRL-5922), NCI-H2347 (ATCC CRL-5942), SW900 (ATCC HTB-59), NCI-H1693 (ATCC CRL-5891), NCI-H1793 (ATCC CRL-5896), NCI-H1650 (ATCC CRL-5883), A549 (ATCC CCL-185), NCI-H1975 (ATCC CRL-5908), NCI-H23 (ATCC CRL-5800), MCF-7 (ATCC HTB-22), and MDA-MB-231 (ATCC HTB-26) were obtained from the indicated repositories.

Authentication

None of the cell lines were authenticated.

Mycoplasma contamination

All cell lines tested negative for mycoplasma infection.

Commonly misidentified lines
(See ICLAC register)

No commonly misidentified cell lines were used.

Animals and other research organisms

Policy information about studies involving animals; ARRIVE guidelines recommended for reporting animal research, and Sex and Gender in Research

Laboratory animals

8-10 week old immunodeficient NSG mice (Janvier) were used in this study.

Wild animals

The study did not involve wild animals.

Reporting on sex

Female and male mice were used in this study.

Field-collected samples

The study did not involve field-collected samples.

Ethics oversight

All animal experiments were approved by the regional board Karlsruhe, Germany (G-81/20 and G52-25)

Note that full information on the approval of the study protocol must also be provided in the manuscript.

Plants

Seed stocks	N/A
Novel plant genotypes	N/A
Authentication	N/A

Flow Cytometry

Plots

Confirm that:

☒ The axis labels state the marker and fluorochrome used (e.g. CD4-FITC).

☒ The axis scales are clearly visible. Include numbers along axes only for bottom left plot of group (a 'group' is an analysis of identical markers).

☒ All plots are contour plots with outliers or pseudocolor plots.

☒ A numerical value for number of cells or percentage (with statistics) is provided.

Methodology

Sample preparation	adherent cells were washed with PBS and incubated in Versene-EDTA (Lonza) at RT for 10 to 15 minutes prior to harvesting using FACS buffer (3% FCS in PBS). 3×105 cells per condition were transferred to previously annotated wells in a V-bottom 96-well plate. Upon washing, cells were stained with 100 µg/mL MPZL1-ChAb for 45 minutes at 4°C. Next, cells were washed and stained with a secondary antibody (AF488® or APC anti-human IgG Fc, BioLegend) during 45 minutes at 4°C in the dark. After a final washing step, cells were resuspended in 200 µL FACS buffer containing 1:1000 SYTOX® Blue (Invitrogen), and transferred to cell strainer snap cap FACS tubes (Corning) for FACS analysis using a BD LSRFortessa flow cytometer (BD Biosciences).<br>For cell surface staining, 1×105 DTCs per condition were used, with JIMT-1 and Molm13 cells included as positive and negative controls, respectively. All samples were first incubated with 5 µg/mL of Human BD Fc BlockTM (BD PharmingenTM) for 10 minutes at RT to minimize nonspecific antibody binding to Fc receptors. Directly afterwards, cells were incubated with 5 µg/mL of labeled antibody for 1 hour at 4°C in the dark. After two washing steps with PBS, cells were resuspended in 200 µL FACS buffer containing 1:1000 of SYTOX® Blue (Invitrogen). Samples were acquired in a BD LSRFortessa flow cytometer (BD Biosciences).<br>Peripheral blood mononuclear cells (PBMCs) were separated with Lymphocyte Separation Medium (Corning) via density gradient centrifugation. Isolated PBMCs were cryopreserved in FCS containing 10% DMSO. T cells were purified from thawed PBMCs by negative selection using magnetic-activated cell sorting (MACS) with a Pan T Cell Isolation Kit (Miltenyi Biotec), following the manufacturer's instructions. Isolated T cells were stimulated with CD3/CD28 human T cell Activator Dynabeads (Gibco) in a 1:1 ratio, and plated at 1×106 cells per mL in RPMI medium supplemented with FCS and penicillin/streptomycin, and 5 ng/mL of interleukin-7 (IL7) and interleukin-15 (IL15) cytokines (Miltenyi Biotec).
Instrument	Cytek® Aurora (Cytek Biosciences) or LSRFortessa (BD Biosciences).
Software	Data analysis was carried out with FlowJo v10
Cell population abundance	Cell populations were isolated by FACS using the gating strategy described in the Methods. Representative flow cytometry plots are provided in the Source Data and Supplementary Data files. No independent additional post-sort purity assessment was performed.
Gating strategy	The starting cell popluation was gated on a SSC-A/FSC-A plot. Cell singlets were identified by FSC/SSC gating. Positive/neagtive populations were determined by FMO controls

☒ Tick this box to confirm that a figure exemplifying the gating strategy is provided in the Supplementary Information.
