# Supplementary material for: Cancer-driver-agnostic targeting of amplified surface proteins identifies MPZL1 for selective CAR-T cell therapy
Source: Nat Commun. 2026 Sep 10;17:9695. doi: 10.1038/s41467-026-77465-5 (PMC13562587; doi:10.1038/s41467-026-77465-5)
Supplement: Supplementary file 3 [file 41467_2026_77465_MOESM3_ESM.pdf]

nature portfolio

Peer Review File

Exploiting amplified cell surface proteins as cancer-driver-agnostic targets uncovers overexpression of MPZL1 for selective CAR-T cell therapy in solid cancers

Corresponding Author: Professor Darjus Tschaharganeh

This file contains all reviewer reports in order by version, followed by all author rebuttals in order by version.

Version 0:

Reviewer comments:

Reviewer #1

(Remarks to the Author)
Applicants zoom in on "passenger" genes that get co-amplified with bona fide oncogenes in cancer that could be targets for therapy. The focus on membrane proteins, given their targetability by antibodies or CART cells. While much "gold" has already been mined here, they come up with an interesting new target, MPZL1, and show that there is considerable tumor-selectivity in its expression. They then generate antibodies to the extracellular domain of the protein and use this to make CART cells. Their in vitro and in vivo data with these CART are compelling and well controlled, given that isogenic cell lines are used with and without expression of MPZL1. An inherent weakness of the study is that the CART does not recognize mouse Mpzl1, which does not allow for a full evaluation of the potential toxicity of a MPZL1 CART in the human setting. The authors acknowledge this shortcoming in the discussion and point towards other targets that have low expression in normal tissues also.

The authors should revise Figure 1J as the numbers are not aligned with the lanes in the gel. Please also specify what was used as a positive control (assuming P stands for positive control?).

Reviewer #2

(Remarks to the Author)
In this study, the authors identify membrane protein MPZL1 as a potential therapeutic target for solid cancers. Through pan-cancer in silico analysis focused on cell surface genes regulated by copy number alterations, the authors identify 6 candidates and select MPZL1 for further investigation. They demonstrate membrane expression of MPZL1 across various solid cancers, generate an antibody (MPZL1-ChAb) targeting the extraceullar domains of MPZL1, and develop MPZL1 CAR-T cells. These CAR-T cells are then evaluated in xenograft models of human HCC and breast cancer, as well as in autochthonous HCC models. Overall, these findings are potentially interesting and clinically relevant. The manuscript presents potentially impactful work developing MPZL1 CAR-T and antibody modalities. However, several conceptual and experimental gaps must be addressed before the manuscript can be considered for publication.
1, The authors need to provide a clear definition of how “driver” and “passenger” copy-number amplifications were distinguished in their analysis. The current rationale for classifying MPZL1 amplification as a passenger event is insufficient, especially given prior studies demonstrating that MPZL1 is frequently amplified and functionally promotes metastasis in liver cancer.
2, The authors conclude that MPZL1 is a non-essential gene and therefore suggest that MPZL1-ChAb is not expected to function as a therapeutic antibody. This conclusion requires much stronger evidence. For example, the authors should provide experimental data showing whether MPZL1-ChAb alone has anti-tumor effects in HCC or other MPZL1-high xenograft models. Reconcile their conclusion with the fact that MPZL1 has been reported to exert oncogenic functions in several cancers, including HCC and NSCLC.
3, Because MPZL1 is expressed in some normal tissues, albeit at lower levels, the authors need to evaluate the potential toxicities of MPZL1-targeting CAR-T cells. Assessment of toxicity in relevant preclinical models is critical.
4, For the autochthonous HCC studies induced by HTVI, the authors use NSG mice, which lack an intact immune system. Given that CAR-T cell function and toxicity are highly influenced by the host immune environment, the authors should use

immunocompetent C57BL/6 mice for the HTVI-based HCC model.

Reviewer #3

(Remarks to the Author)
Summary
This manuscript presents an ambitious end-to-end study that starts from a pan-cancer multiomics screen for amplified cell surface proteins and culminates in the preclinical development of MPZL1-directed CAR T-cells. The authors propose that passenger somatic copy number amplifications affecting genes that encode cell surface proteins can be exploited therapeutically as “Trojan horse” targets, and they use MPZL1, a glycosylated cell surface receptor on chromosome 1q, as a worked example. MPZL1 is reported to be amplified in up to roughly seventy-five percent of cases in several solid cancers, with immunohistochemistry suggesting tumour restricted membrane localisation. The authors generate a monoclonal antibody against MPZL1, engineer MPZL1 directed CAR T cells, and demonstrate efficacy in vitro and in xenograft models.

Overall, this is a broad and technically impressive piece of work with clear translational intent. The end-to-end nature of the pipeline, spanning in silico target discovery, antibody and CAR generation, functional validation, and in vivo efficacy, makes the study feel self-contained and suitable for a high-impact translational journal. At the same time, the scope sometimes makes the manuscript feel like three papers in one. As a reader, I did feel some fatigue simply due to the volume of data. In my view, the main remaining issues relate to the narrative focus and conceptual framing around passenger amplifications and Trojan horse targets, and to translational nuances around MPZL1 expression in normal tissues, antigen density, and target selection.

Major comments

1. The manuscript is very comprehensive, which is a strength, but it can at times feel like three papers in one. It reads as a target discovery study, an antibody and CAR engineering paper, and a translational in vivo efficacy paper, rather than a single tightly focused narrative. Important aspects of the work, particularly limitations, edge cases, and less tidy results, risk being buried within the volume of data. This may make it harder for readers outside the immediate subfield to identify and retain the main message.

Some of the more technical or confirmatory experiments could be moved to the supplementary information without reducing the overall impact.

2. As a concept, the Trojan horse metaphor is somewhat confusing in this setting. Traditionally a Trojan horse is something dangerous hidden inside an apparently benign structure that is deliberately brought inside. MPZL1 is an endogenous protein that the tumour naturally expresses. You are exploiting it as a vulnerability rather than smuggling in a deceptive payload.

If one wants to stay with Greek metaphors, MPZL1 seems more analogous to an Achilles heel than a Trojan horse. This may be a matter of taste, but the current framing did make me pause. It may help to sharpen and standardise the language around passenger amplification and therapeutic entry point rather than relying heavily on the Trojan horse image.

3. Early in the manuscript the authors identify frequently amplified genes on chromosomal arms 1q and 8q and focus on 1q as a hotspot for amplified cell surface receptors. The authors then narrow down nineteen differentially expressed 1q cell surface protein genes and select those annotated as plasma membrane proteins, from which MPZL1 is chosen as a potentially targetable bystander amplification.

(A) Did you systematically compare the expression profiles and tractability of other 1q cell surface proteins such as ATP1B1, EFNA4, IGSF8, ADAM15, and others? Given that you argue that the exact role of MPZL1 in cancer biology is not essential for its use as a targeting moiety, what disqualified other candidates?

(B) Related to this, could MPZL1 be more than a pure passenger? The evidence presented in this section appears largely correlative, based on copy number frequency and expression. Have you examined whether MPZL1 copy number or expression associates with survival? Perhaps a clearer definition of “driver” versus “passenger” needs to be articulated.

4. The authors note that the pipeline relies on a single open access resource for membrane bound proteins. Why restrict yourself to one resource at the outset? It would be reassuring to know that the analysis has been cross validated against, or could readily incorporate, additional databases such as the Human Protein Atlas, UniProtKB, MemProtMD, UniTmp, or AmiGO 2.

Even if you prefer to keep the current pipeline unchanged for this manuscript, a short discussion of how expanding to multiple resources might affect target discovery, and whether MPZL1 would still have been prioritised, would clarify the robustness of your approach.

5. The authors use GEPIA2 to compare expression of the cell surface protein genes in tumour versus matched normal tissues, for example liver cancer versus normal liver or breast cancer versus normal breast. However, restricting analysis to counterpart tissues misses expression in other critical organs. For example, data from the Human Protein Atlas indicate rather ubiquitous MPZL1 expression across normal tissues. (A) It would strengthen the translational argument to address

this more directly, perhaps by showing broader normal tissue expression profiles at the RNA level.

Later in the manuscript, you present your own immunohistochemistry across a panel of normal tissues. (B) It would be helpful to reconcile these internal findings with the public databases. To what extent are they concordant or discordant, and how do you interpret any discrepancies?

In Figure 2D, where you compare normal and tumour tissue, the immunohistochemistry pattern is intriguing. (C) Are you proposing that MPZL1 is largely intracellular in normal physiology but becomes more prominently membrane bound in cancer? If so, can you speculate on a mechanism, for example altered trafficking, post translational modifications, or interactions with other partners? If not, clarifying what you think the immunohistochemistry is showing would help.

6. The authors score normal tissues as immunohistochemistry zero to two, but CAR T cells are known to be exquisitely sensitive to antigen density and can attack normal cells that express even low levels of target antigen, particularly in critical organs. (A) Could you comment more explicitly on how your immunohistochemistry scoring, and any quantitative estimates of antigen density if available, map onto likely CAR T cell recognition thresholds?

(B) More broadly, the functional data you present are largely in MPZL1 high cell lines and models. We see convincing efficacy in high antigen contexts, with multiple entities, isogenic pairs, effector to target titrations, cytokine and exhaustion phenotyping, but less about intermediate or low antigen density. This is clinically very relevant given the heterogeneity of MPZL1 expression across tumours.

7. You report that patient 8843 has a high MPZL1 immunohistochemistry score but does not show strong binding of MPZL1 antibody. Could you expand on your interpretation of this discrepancy? Does it suggest epitope variability, differences in glycosylation, or other post translational or structural heterogeneity across tumours? This seems highly relevant for both antibody and CAR design.

This point also intersects with the concern about antigen density and heterogeneity. The presence of immunohistochemistry positive yet weakly binding samples might have implications for on target efficacy and potential antigen escape.

8. Figure 2 contains extensive experimental work characterising MPZL1 as a cell surface protein. While this is impressive, much of this information might already be inferred from existing online resources. The purpose of such databases is partly to remove the need for exhaustive re validation.

It would be helpful to state explicitly whether this experimental characterisation was performed to resolve inconsistencies or ambiguities in the databases, to establish best practice in the context of CAR T development, or for another reason. Making this motivation explicit in the narrative could help the reader understand why this level of experimental detail is included in the main figures. (see point #1 – could this be supplement?)

Minor comments

1. Notation and terminology
In running text, chromosome 1q reads more smoothly than chr. 1q. If a short form is preferred, chr1q is cleaner.

Please use Mb for megabases throughout. (example: p 4 line 146)

The phrase describing MPZL1 antibody as a carrier for a therapeutic agent feels slightly imprecise in the CAR T context, where the antibody derived single chain fragment primarily serves as the targeting moiety rather than a classical carrier. Consider rephrasing for clarity, for example that it serves as a targeting domain for therapeutic effectors. (page 7 line 261-262)

2. Grammar check: Page 8 line 282 & Page 9 line 300. “new generated” is ungrammatical & comma after “immunohistochemistry”.

3. At one point (p5 line 173/174) you refer to the patient cohort as unique. As currently phrased, this could be read as implying that the tumours themselves are biologically unusual. I think what you mean, and what is indeed impressive, is that you have multiple modalities on the same tissue, for example copy number, expression, immunohistochemistry and so on, which is technically non-trivial and valuable.

I suggest rephrasing to emphasise that the dataset, namely multi modal profiling on matched samples, is unique, rather than the patients tumours themselves.

Reviewer #4

(Remarks to the Author)
This manuscript proposes MPZL1 as a potential target for CAR T-cell therapy.

Major Concerns

The authors claim that MPZL1 is overexpressed in tumor tissues but minimally expressed in normal tissues based on

immunohistochemistry (IHC) data generated using a rabbit monoclonal antibody (clone D17B10). However, it is unclear whether this antibody is truly specific to MPZL1 and does not cross-react with other human proteins. This specificity should ideally be validated using platforms such as Retrogenix Cell Microarray or Origene High-Density Protein Microarray. That said, performing such costly specificity assays with an antibody that is not intended for future CAR development may be of limited practical value. Consequently, many groups instead generate their own antibodies that are compatible with both IHC and CAR construction and then perform comprehensive specificity screening using Retrogenix or equivalent platforms. For clinical CAR T-cell development, regulatory agencies strongly recommend—or effectively require—that the antibody used for CAR generation undergo rigorous specificity validation. In the absence of such data, it is premature and undesirable to conclude that MPZL1 is an appropriate target for CAR T-cell therapy.

Optional Future Direction

As MPZL1 represents a novel target for CAR T-cell therapy, future studies could consider generating a mouse surrogate antibody, developing mouse CAR T cells targeting murine MPZL1, and evaluating antitumor efficacy and on-target/off-tumor toxicity in an immunocompetent mouse model. Such work would further strengthen the translational relevance of MPZL1-directed CAR T-cell therapy but is not essential for the conclusions of the current manuscript. The authors might include this point in the discussion.

Minor Concerns

1) It can be controversial to say MPZL1 the true ‘passenger’

The authors show that MPZL1 overexpression or knockout does not alter cell growth or colony formation in cell lines, supporting the hypothesis that MPZL1 behaves as a non-essential bystander in tumorigenesis. However, there are previous reports showing that MPZL1 promotes tumor cell proliferation, migration and invasion [Oncology reports, 2019, 42.2: 679-687. Cell research, 2014, 24.2: 204-217. Genes & Diseases, 2024, 11.5: 101085. Carcinogenesis, 2022, 43.10: 919-929. Human Gene Therapy, 2023, 34.11-12: 540-553.]. The authors might include this point in the discussion.

2) Additional methodological detail for monoclonal antibody generation

Describing more experimental methods and results will improve the confidence in the MPZL1-ChAb. Such as: Details of antigen, Number of mice immunized, immunization schedule (number of boosts and intervals), How the titer of immunization was determined and how the ‘strong titers’ threshold was defined.

3) In the discussion part, this article draws parallels to the case of claudin 18.2 and HER2, where expression is present in normal tissues but still compatible with therapy in carefully selected patients. However, MPZL1 expression is clearly detectable, even though it is often at low levels (Figure 2F). I would suggest slightly tempering strong safety statements and more explicitly emphasizing that safety remains to be established experimentally, such as in cross-reactive preclinical models.

4) Clarify differences between MPZL 1.23/1.44 and MPZL 1.24/1.66

It is not entirely clear to the reader what distinguishes MPZL1.23 from 1.44, or MPZL1.24 from 1.66, beyond the VL-VH versus VH-VL orientation.

5) Terminology for cytokine measurement

As Brefeldin A +Monensin are used to block secretion of cytokines and retain cytokines inside the cells, it may be more accurate to describe these data as cytokine ‘production’ or ‘generation’ or ‘intracellular accumulation’ rather than ‘release’ or ‘secretion’

6) Figure numbers appear to be swapped. (Line #229, 235)

Figure 2F > Figure 2E , Figure 2G > Figure 2F

Reviewer #5

(Remarks to the Author)
I co-reviewed this manuscript with one of the reviewers who provided the listed reports. This is part of the Nature Communications initiative to facilitate training in peer review and to provide appropriate recognition for Early Career Researchers who co-review manuscripts.

Version 1:

Reviewer comments:

Reviewer #1

(Remarks to the Author)

I am satisfied with the answers to my queries. I support publication.

Reviewer #2

(Remarks to the Author)
The authors have satisfactorily addressed my concerns. I have no other comments.

Reviewer #3

(Remarks to the Author)
Thank you for the thoughtful and comprehensive revision. I appreciate the care with which you addressed my comments. I found the revised framing much clearer, particularly the decision to move away from the "passenger" and "Trojan horse" terminology and to position MPZL1 more carefully as a target that does not appear to function as a classical growth driving oncogene under the conditions tested. The additional analyses using DepMap and the added in vivo comparisons strengthen this point.

I also appreciated the new human tissue explant experiments, which add useful translational context and help address concerns around tumour selectivity and potential activity in matched normal tissues. Importantly, I think the revised manuscript now does a better job of acknowledging the limitations of the current preclinical models and avoiding overstatement. The overall narrative also feels more focused after streamlining some of the more technical material.

Overall, I am satisfied that my main concerns have been addressed and I have no further major comments.

Reviewer #4

(Remarks to the Author)
The authors have adequately addressed all of my concerns. I believe the manuscript is now acceptable for publication.

Reviewer #5

(Remarks to the Author)
I co-reviewed this manuscript with one of the reviewers who provided the listed reports. This is part of the Nature Communications initiative to facilitate training in peer review and to provide appropriate recognition for Early Career Researchers who co-review manuscripts.

Open Access This Peer Review File is licensed under a Creative Commons Attribution 4.0 International License, which permits use, sharing, adaptation, distribution and reproduction in any medium or format, as long as you give appropriate credit to the original author(s) and the source, provide a link to the Creative Commons license, and indicate if changes were made.
In cases where reviewers are anonymous, credit should be given to 'Anonymous Referee' and the source.
The images or other third party material in this Peer Review File are included in the article’s Creative Commons license, unless indicated otherwise in a credit line to the material. If material is not included in the article’s Creative Commons

license and your intended use is not permitted by statutory regulation or exceeds the permitted use, you will need to obtain permission directly from the copyright holder.
To view a copy of this license, visit https://creativecommons.org/licenses/by/4.0/

REVIEWER COMMENTS Point-by-point reply

We thank all the reviewers for their constructive feedback and valuable suggestions.

We noticed that there were two main issues, which were shared by multiple reviewers, which we want to highlight and outline here how we addressed them:

Interpretation of MPZL1 as "passenger amplification"

In the revised manuscript, we have clarified how we define genes with canonical cancer-driving functions. Our working definition is based on functional genomics evidence demonstrating that perturbation of a gene directly alters tumor cell fitness, most commonly assessed through effects on proliferation or survival in large-scale loss-of-function screens or in experimental cancer models.

To avoid potential confusion, we have revised the manuscript and no longer refer to MPZL1 amplification as a "passenger" event. Instead, we now emphasize the available experimental evidence indicating that MPZL1 does not appear to function as a classical growth-driving oncogene.

First, we now include data from the Cancer Dependency Map (Supple. Figure S2A-F) showing that knockout of MPZL1 does not impair proliferation across a broad panel of ca. 1000 cancer cell lines. For comparison, we highlight perturbation effects for RPS11 a core essential gene, where genetic perturbation results in clear growth defects across all cell lines (A), for OR5L2 a non-essential gene, where KO does not result in clear growth defects across all cell lines (C), for canonical oncogenic dependencies such as ERBB2 (D), and KRAS (B), and synthetic lethal associations for MFRN2 (E), where genetic perturbation results in clear growth defects in subsets of cancer cell lines.

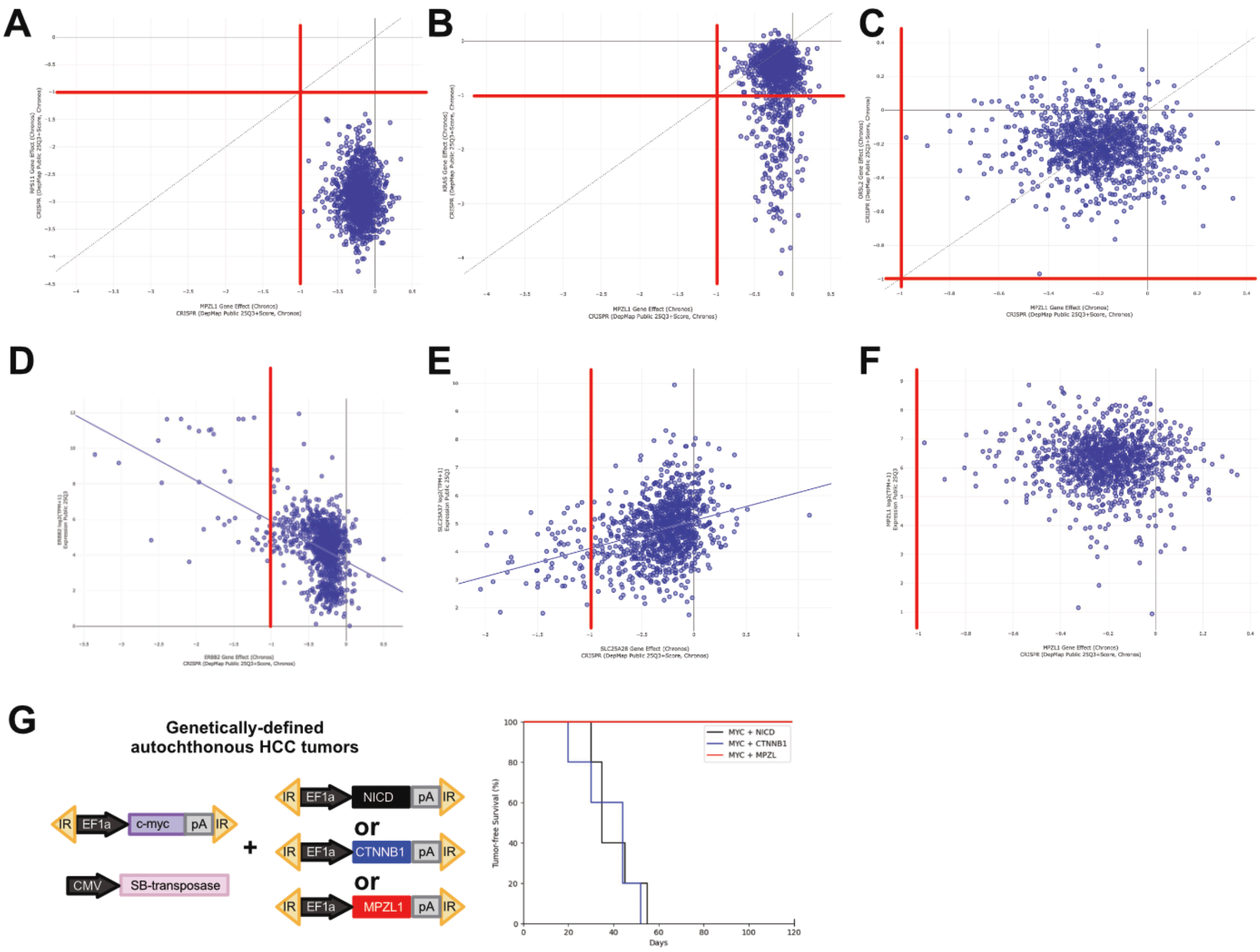

<u>Suppl. Figure S2.</u> MPZL1 is not a classical cancer driver.
(A) – (F) DepMap data analyses. Chronos score < -1 (red lines) indicates strong impairment of cell fitness across ~1000 cancer cell lines tested by means of CRISPR-KO screens (dots). **(A)** Chronos scores for MPZL1 KO (x-axis) versus RPS11 (core essential gene) KO (y-axis) **(B)** Chronos scores for MPZL1 KO (x-axis) versus KRAS (well-established cancer driver in the subset of mutant KRAS cells) KO (y-axis). **(C)** Chronos scores for MPZL1 KO (x-axis) versus OR5L2 (olfactory receptor; core non-essential gene) KO (y-axis). **(D)** Chronos scores for ERBB2 KO (x-axis) versus ERBB2 mRNA expression (y-axis).**(E)** Chronos scores for SLC25A28/MFRN2 KO (x-axis) versus SLC25A37/MFRN1 mRNA expression (y-axis). **(F)** Chronos scores for MPZL1 KO (x-axis) versus MPZL1 mRNA expression (y-axis). (**G) HTVI liver cancer mouse models tested**. MYC + MPZL1 did not results in tumor development. (n=5 animals).

Second, we provide additional in vivo evidence from our hydrodynamic tail vein injection liver cancer model. By testing MPZL1 overexpression in comparison to two known HCC-cancer drivers we show that, while expression of MYC together with either activated NOTCH1 (NICD) or mutant CTNNB1 results in rapid tumor development, we did not observe tumor formation upon co-expression of MYC and MPZL1 (Supple. Figure S2E). Furthermore, we now better highlight that MPZL1 overexpression did not alter tumorigenesis in the MYC + p53 knockout context (as already indicated in the original manuscript), nor did it induce lung metastasis in this model. We now state:

"…we first generated a cohort of MycOE;p53-/-;GFP mice and MycOE;p53-/-;MPZL1 mice without CAR-T cell treatment and found that tumor onset and survival of both

groups did not show significant differences… Further, detailed necropsy revealed no pulmonary metastases in both the MycOE;p53-/-;MPZL1 and MycOE;p53-/-;GFP cohorts, arguing against a critical role for MPZL1 in metastatic progression in this genotypically defined mouse model (data not shown).”

Taken together, these results suggest that MPZL1 does not fulfill the criteria of a classical cancer driver gene required for tumor initiation or maintenance under the experimental conditions tested.

Importantly, we fully acknowledge prior studies reporting oncogenic functions of MPZL1, including roles in migration and invasion. In line with these reports, we now explicitly discuss that MPZL1 whose perturbation does not affect cell proliferation may still contribute to other cancer-relevant phenotypes, such as metastatic behavior. We have revised the manuscript accordingly to better reflect this distinction.

“While additional work is required to definitively establish a causal relationship between MPZL1 expression and metastatic potential, as has previously been suggested [33], [36], [37], MPZL1-directed therapy may accordingly target tumor cells with elevated metastatic capacity lending further support for focusing on this cell surface receptor. “

And

“Yet, even if we did not observe associations between elevated MPZL1 expression and metastasis potential in the autochthonous liver cancer mouse model tested in our study, MPZL1 has been proposed to promote migratory and invasive capabilities [33], [36], [37]. Therefore, MPZL1-directed therapy may preferentially target tumor cells with enhanced metastatic capacity, potentially providing an added therapeutic benefit.”

Furthermore, we have included additional experimental work to evaluate MPZL1 CAR-T cell activity in a human tissue context (Figure 6, Supple. Figure S8). Specifically, we performed experiments using human tissue explant models including tumor samples and patient-matched normal tissues to assess CAR-T cell infiltration and effector responses. These additional experiments strengthen the translational relevance of the study and provide further support for the tumor-selective activity of MPZL1 CAR-T cells. In recognition of their contributions to these experiments, we have included additional co-authors.

Concern about antitumor efficacy and on-target/off-tumor toxicity

This concern was shared multiple reviewers and some of them suggested to test MPZL1 CAR-T cells in an immunocompetent mouse model. This approach was also initially pursued by us but we failed in identifying an appropriate murine antibody targeting MPZL1. Instead of relying on this approach we pursued an even more preclinical and translational approach to test for antitumor efficacy and on-target/off tumor toxicity by employing a human tissue explant model in collaboration with the group of Prof. Halama

(German Cancer Research Center, Heidelberg/ TRON, Mainz), which pioneered this experimental system.

Specifically, we utilized a fully human T cell–tracing tissue explant system, including both MPZL1-positive tumor samples from different entities and patient-matched healthy tissue samples (Figure 6, Supple. Figure S8). To this end, freshly resected explant sections were treated with CMFDA-labeled T cells (CLT) and cultivated overnight following examination of infiltration across different human patient samples (Figure 6A, see Methods). Further, T cell activity was assessed via granzyme B (GranB) immunofluorescence staining (Suppl. Figure S8C) and multiplex cytokine analysis. We observed that MPZL1 CAR-T cells displayed markedly higher infiltration into MPZL1 positive tumor explants compared with naïve and activated UT T cells, as well as CAR-T cells targeting carcinoembryonic antigen (CEA), ovarian cancer antigen 125 (CA125) or CD19 that we tested to additionally confirm MPZL1 CAR-T-cell specificity (Figure 6B, C). Positive tissue expression of MPZL1, CA125 and CEA in corresponding patient tissues was confirmed by immunohistochemistry (Suppl. Figure S8A, B). Of note, the high infiltration of MPZL1 CAR-T cells was accompanied by significantly elevated levels of effector molecules, including granzyme B (GranB), CXCL9 (MIG), CXCL10 (IP10), interferon gamma (IFNγ), tumor necrosis factor alpha (TNFα), and interleukin 2 (IL-2), relative to other CAR-T or UT T cells (Figure 6D, E). Importantly, comparison with patient-matched healthy tissue explants revealed lower MPZL1 CAR-T cell infiltration and significantly reduced effector molecule induction in normal tissues (Figure 6E-G). Together, these initial human tissue sample-based results indicate selective activity of MPZL1 CAR-T cells toward tumor cells as opposed to healthy patient-matched human tissue.

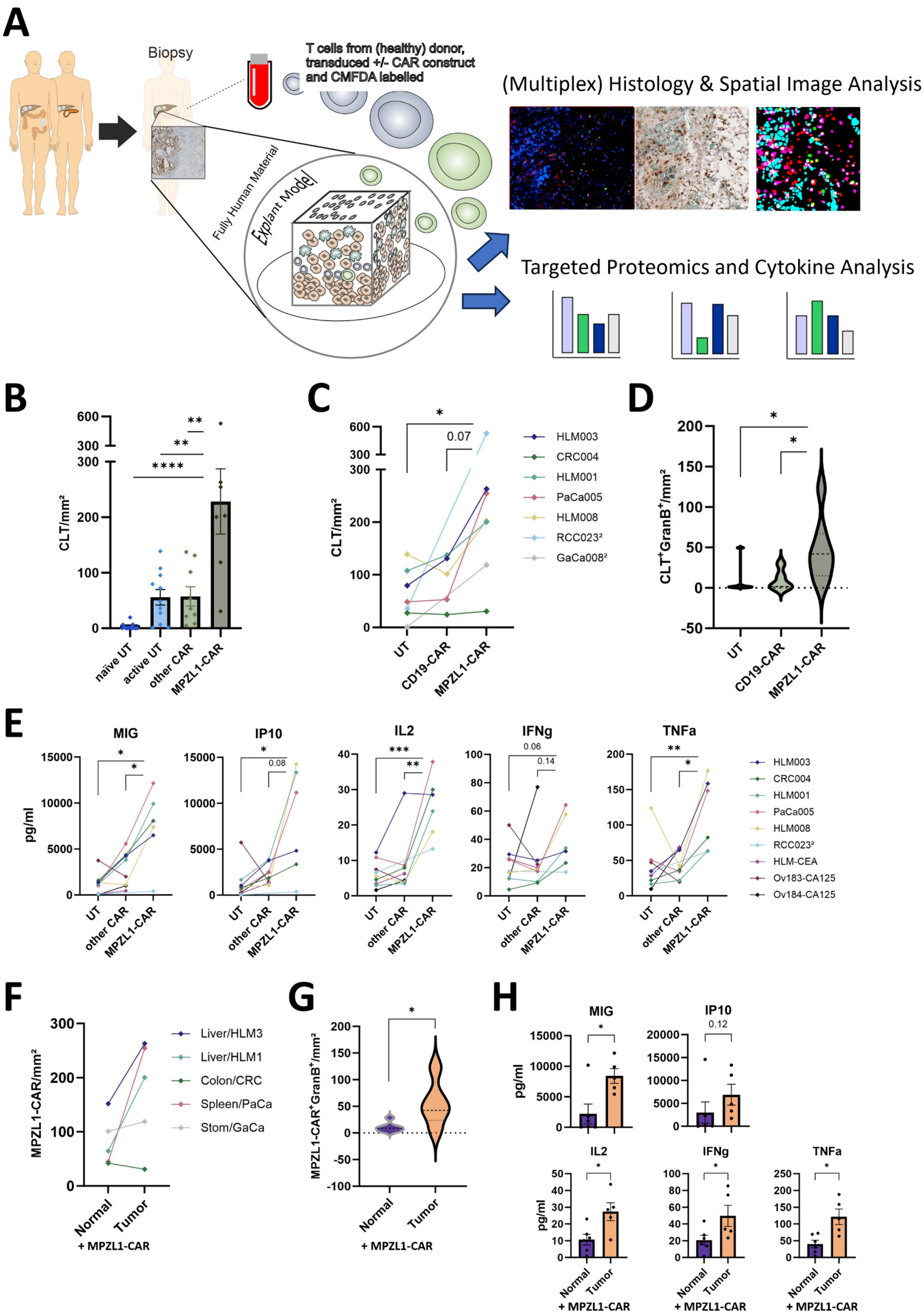

Figure 6: Tumor-specific activation of MPZL1 CAR-T cells in human tissue explants. (A) Schematic of the fully human explant model system for tracing adoptively transferred T cells. Freshly resected human biopsies are treated ex vivo with fluorescent Cell Tracker CMFDA-labeled T cells (CLT). Immunostaining and image analysis allow spatial exploration of T cell infiltration and effects. Targeted proteomics and cytokine analysis detect T cell activation and anti-tumor effects. **(B) – (E) Superior MPZL1 CAR-T cell infiltration and effects in human tumor explants.** In total, 35 patients with 6 different tumor entities (1xCRC: Colorectal Cancer, 29xHLM: Colorectal Cancer Liver Metastases, 1xPaCa: Pancreatic Ductal Adenocarcinoma, 2xOv: Ovarian Cancer, 1xGaCa: Gastric Cancer and 1xRCC: Renal Cancer) were treated with different CLT.

Positive tissue expression of corresponding antigens was confirmed for MPZL1, ovarian cancer-related tumor marker CA125 (CA125) and carcinoembryonic antigen (CEA) CAR-T cells (see Figure S8). **(B)** Infiltration of MPZL1 CAR-CLT (n=7) into human explants compared to naïve UT (n=25), active UT (n=11) and other CAR-CLT (CA125-CAR: n=2, CEA-CAR: n=2, CD19-CAR: n=5). Mean cell numbers per mm² are shown. **(C)** Comparison of MPZL1 CAR-CLT (n=7), CD19 CAR-CLT (n=5) and (active) UT-CLT (n=7) infiltration in explants of same patients. **(D)** Granzyme B (GranB)-positive CLT quantification in explants treated with MPZL1 CAR-CLT (n=7), CD19 CAR-CLT (n=5) and (active) UT-CLT (n=7). In total 7 patients (1xCRC, 3xHLM, 1xPaCa, 1xGaCa, 1xRCC) were treated. **(E)**. Cytokine concentrations measured in human explants treated with MPZL1 CAR-CLT (n=6), (active) UT-CLT (n=9) or other CAR-CLT (CA125-CAR: n=2, CEA-CAR: n=1, CD19-CAR: n=5). The same samples as shown in **(B)** were utilized .**(F) – (H) Marginal MPZL1 CAR-T cell infiltration and effects in human normal tissue explants.** In total, normal tissue explants of 6 patients (3x Liver, 1xColon, 1xSpleen, 1xStom=Stomach) and 5 corresponding tumors (2xHLM, 1xCRC, 1xGaCa, 1xPaCa) were treated with MPZL1 CAR-CLT. Corresponding MPZL1 tissue expression is shown in Figure S8. **(F)** Infiltration of MPZL1 CAR-CLT into normal tissue and corresponding tumor explants of same patients (n=5). **(G)** GranB-positive CLT quantification in normal tissue (n=6) and tumor explants (n=5) treated with MPZL1 CAR-CLT. **(H)**. Cytokine concentrations measured in normal tissue (n=6) and tumor explants (n=5) treated with MPZL1 CAR-CLT. **(B) – (H)** CLT quantification was performed after tissue processing and immunofluorescence staining of GranB following fully automatic fluorescence microscopy (nuclei=DAPI=blue, CLT=FITC=green, GranB=Cy5=red). Colored lines relate to explants of same patients. Error bars indicate standard error of the mean (SEM). Violine plots show median, 75th and 25th percentiles (middle, top and bottom line). Asterisks indicate significant Mann–Whitney U test (*$p < 0.05$, **$p < 0.01$, ***$p < 0.001$, ****$p < 0.0001$, ns = not significant). ²UT-CLT and CD19-CAR were not tested and comparison of MPZL1-CAR with untreated control of same patient shown.

Despite this encouraging new data from tissue explant assays, we have again highlighted the limitations of our current approaches:

“While our studies do not fully address systemic on-target/off-tumor toxicities—given the lack of cross-reactivity with murine MPZL1, the use of immunocompromised mouse models, and the limited availability of healthy patient-matched tissue samples from multiple organs suitable for tissue explant assays—existing examples from other tumor-associated antigens suggest that limited expression in normal tissues as opposed to markedly elevated tumor-specific expression can be compatible with tolerable safety profiles for surface protein targeting therapeutic approaches.”

Apart from these overarching points we also addressed all points raised by individual reviewers as outlined below in the detailed point-by-point response:

Reviewer #1 (Remarks to the Author):

Applicants zoom in on "passenger" genes that get co-amplified with bona fide oncogenes in cancer that could be targets for therapy. The focus on membrane proteins, given their targetability by antibodies or CART cells. While much "gold" has already been mined here, they come up with an interesting new target, MPZL1, and show that there is considerable tumor-selectivity in its expression. They then generate antibodies to the extracellular domain of the protein and use this to make CART cells. Their in vitro and in vivo data with these CART are compelling and well controlled, given that isogenic cell lines are used with and without expression of MPZL1. An inherent weakness of the study is that the CART does not recognize mouse Mpzl1, which does not allow for a full evaluation of the potential toxicity of a MPZL1 CART in the human setting. The authors acknowledge this shortcoming in the discussion and point towards other targets that have low expression in normal tissues also.

We thank the reviewer for the kind feedback and for pointing out that the in vitro and in vivo data with our MPZL1 CAR-T cells are compelling and well controlled.

We also agree that evaluation of MPZL1-directed CAR-T-cell therapy in immunocompetent models using murine-specific reagents would strengthen the translational assessment of our approach. However, as our current CAR construct specifically recognizes human MPZL1 and does not cross-react with the murine protein, such experiments would require the development of additional antibodies and corresponding murine CAR-T cells, which until now was unfortunately not successful, despite our continues attempts to achieve this goal.

We would therefore like to note that we have now tried to address this limitation by leveraging a preclinical model closely reflecting human biology, in that we utilized a fully human T cell–tracing tissue explant system, including both MPZL1-positive tumor samples from different entities and patient-matched healthy tissue samples (Figure 6, Supple. Figure S8). To this end, freshly resected explant sections were treated with CMFDA-labeled T cells (CLT) and cultivated overnight following examination of infiltration across different human patient samples (Figure 6A, see Methods). Further, T cell activity was assessed via granzyme B (GranB) immunofluorescence staining (Suppl. Figure S8C) and multiplex cytokine analysis. We observed that MPZL1 CAR-T cells displayed markedly higher infiltration into MPZL1 positive tumor explants compared with naïve and activated UT T cells, as well as CAR-T cells targeting carcinoembryonic antigen (CEA), ovarian cancer antigen 125 (CA125) or CD19 that we tested to additionally confirm MPZL1 CAR T-cell specificity (Figure 6B, C). Positive tissue expression of MPZL1, CA125 and CEA in corresponding patient tissues was confirmed by immunohistochemistry (Suppl. Figure S8A, B). Of note, the high infiltration of MPZL1 CAR-T cells was accompanied by significantly elevated levels of effector molecules, including granzyme B (GranB), CXCL9 (MIG), CXCL10 (IP10), interferon gamma (IFNγ), tumor necrosis factor alpha (TNFα), and interleukin 2 (IL-2), relative to other CAR-T or UT T cells (Figure 6D, E). Importantly, comparison with patient-matched healthy tissue explants revealed lower MPZL1 CAR-T cell infiltration and significantly reduced effector molecule induction in normal tissues (Figure 6E-G). Together, these initial human tissue

sample-based results indicate selective activity of MPZL1 CAR-T cells toward tumor cells as opposed to healthy patient-matched human tissue.

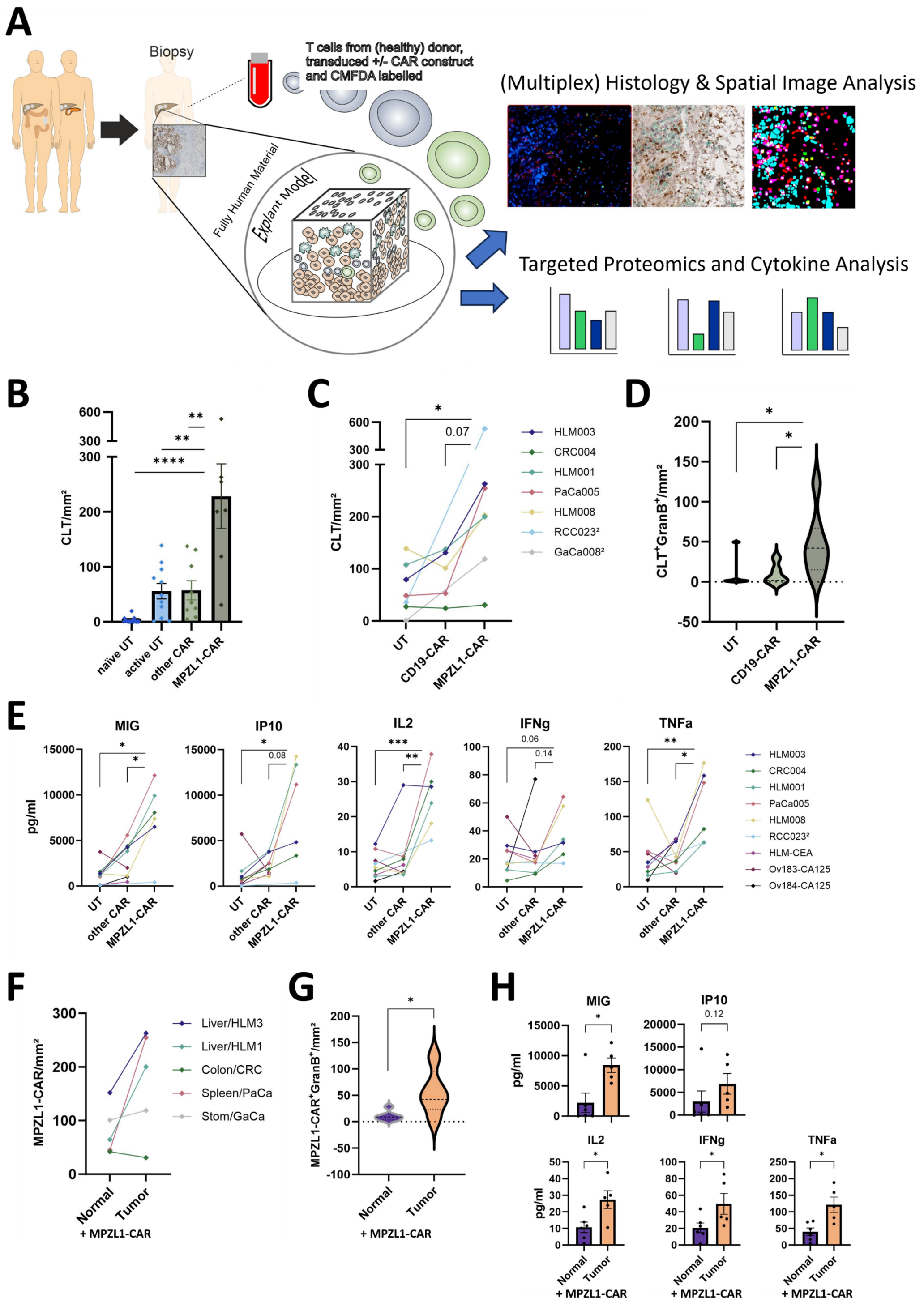

Figure 6: Tumor-specific activation of MPZL1 CAR-T cells in human tissue explants. (A) Schematic of the fully human explant model system for tracing adoptively transferred T cells. Freshly resected human biopsies are treated ex vivo with fluorescent Cell Tracker CMFDA-labeled T cells (CLT). Immunostaining and image analysis allow spatial exploration of T cell infiltration and effects. Targeted proteomics and cytokine analysis detect T cell activation and anti-tumor effects. **(B) – (E) Superior MPZL1 CAR-**

T cell infiltration and effects in human tumor explants. In total, 35 patients with 6 different tumor entities (1xCRC: Colorectal Cancer, 29xHLM: Colorectal Cancer Liver Metastases, 1xPaCa: Pancreatic Ductal Adenocarcinoma, 2xOv: Ovarian Cancer, 1xGaCa: Gastric Cancer and 1xRCC: Renal Cancer) were treated with different CLT. Positive tissue expression of corresponding antigens was confirmed for MPZL1, ovarian cancer-related tumor marker CA125 (CA125) and carcinoembryonic antigen (CEA) CAR-T cells (see Figure S8). **(B)** Infiltration of MPZL1 CAR-CLT (n=7) into human explants compared to naïve UT (n=25), active UT (n=11) and other CAR-CLT (CA125-CAR: n=2, CEA-CAR: n=2, CD19-CAR: n=5). Mean cell numbers per mm² are shown. **(C)** Comparison of MPZL1 CAR-CLT (n=7), CD19 CAR-CLT (n=5) and (active) UT-CLT (n=7) infiltration in explants of same patients. **(D)** Granzyme B (GranB)-positive CLT quantification in explants treated with MPZL1 CAR-CLT (n=7), CD19 CAR-CLT (n=5) and (active) UT-CLT (n=7). In total 7 patients (1xCRC, 3xHLM, 1xPaCa, 1xGaCa, 1xRCC) were treated. **(E)**. Cytokine concentrations measured in human explants treated with MPZL1 CAR-CLT (n=6), (active) UT-CLT (n=9) or other CAR-CLT (CA125-CAR: n=2, CEA-CAR: n=1, CD19-CAR: n=5). The same samples as shown in **(B)** were utilized .**(F) – (H) Marginal MPZL1 CAR-T cell infiltration and effects in human normal tissue explants.** In total, normal tissue explants of 6 patients (3x Liver, 1xColon, 1xSpleen, 1xStom=Stomach) and 5 corresponding tumors (2xHLM, 1xCRC, 1xGaCa, 1xPaCa) were treated with MPZL1 CAR-CLT. Corresponding MPZL1 tissue expression is shown in Figure S8. **(F)** Infiltration of MPZL1 CAR-CLT into normal tissue and corresponding tumor explants of same patients (n=5). **(G)** GranB-positive CLT quantification in normal tissue (n=6) and tumor explants (n=5) treated with MPZL1 CAR-CLT. **(H)**. Cytokine concentrations measured in normal tissue (n=6) and tumor explants (n=5) treated with MPZL1 CAR-CLT. **(B) – (H)** CLT quantification was performed after tissue processing and immunofluorescence staining of GranB following fully automatic fluorescence microscopy (nuclei=DAPI=blue, CLT=FITC=green, GranB=Cy5=red). Colored lines relate to explants of same patients. Error bars indicate standard error of the mean (SEM). Violine plots show median, 75th and 25th percentiles (middle, top and bottom line). Asterisks indicate significant Mann–Whitney U test (*p < 0.05, **p < 0.01, ***p < 0.001, ****p < 0.0001, ns = not significant). ²UT-CLT and CD19-CAR were not tested and comparison of MPZL1-CAR with untreated control of same patient shown.

Despite this encouraging new data from tissue explant assays, we have again highlighted the limitations of our current approaches:

"While our studies do not fully address systemic on-target/off-tumor toxicities—given the lack of cross-reactivity with murine MPZL1, the use of immunocompromised mouse models, and the limited availability of healthy patient-matched tissue samples from multiple organs suitable for tissue explant assays—existing examples from other tumor-associated antigens suggest that limited expression in normal tissues as opposed to markedly elevated tumor-specific expression can be compatible with tolerable safety profiles for surface protein targeting therapeutic approaches."

The authors should revise Figure 1J as the numbers are not aligned with the lanes in the gel. Please also specify what was used as a positive control (assuming P stands for positive control?).

Thank you for pointing this out. We have revised Fig 1J to align numbers with the lanes shown. We have indicated what was used as a positive control (PC) in the legend.

Reviewer #2 (Remarks to the Author):
In this study, the authors identify membrane protein MPZL1 as a potential therapeutic target for solid cancers. Through pan-cancer in silico analysis focused on cell surface genes regulated by copy number alterations, the authors identify 6 candidates and select MPZL1 for further investigation. They demonstrate membrane expression of MPZL1 across various solid cancers, generate an antibody (MPZL1-ChAb) targeting the extraceullar domains of MPZL1, and develop MPZL1 CAR-T cells. These CAR-T cells are then evaluated in xenograft models of human HCC and breast cancer, as well as in autochthonous HCC models. Overall, these findings are potentially interesting and clinically relevant. The manuscript presents potentially impactful work developing MPZL1 CAR-T and antibody modalities.

We thank the reviewer for highlighting that our manuscript presents potentially impactful work.

However, several conceptual and experimental gaps must be addressed before the manuscript can be considered for publication.

1, The authors need to provide a clear definition of how “driver” and “passenger” copy-number amplifications were distinguished in their analysis. The current rationale for classifying MPZL1 amplification as a passenger event is insufficient, especially given prior studies demonstrating that MPZL1 is frequently amplified and functionally promotes metastasis in liver cancer.

We thank the reviewer for raising this point. In the revised manuscript, we have clarified how we define genes with canonical cancer-driving functions. Our working definition is based on functional genomics evidence demonstrating that perturbation of a gene directly alters tumor cell fitness, most commonly assessed through effects on proliferation or survival in large-scale loss-of-function screens or in experimental cancer models.

To avoid potential confusion, we have revised the manuscript and no longer refer to MPZL1 amplification as a “passenger” event. Instead, we now emphasize the available experimental evidence indicating that MPZL1 does not appear to function as a classical growth-driving oncogene.

First, we now include data from the Cancer Dependency Map (Supple. Figure S2A-F) showing that knockout of MPZL1 does not impair proliferation across a broad panel of ca. 1000 cancer cell lines. For comparison, we highlight perturbation effects for RPS11 a core essential gene, where genetic perturbation results in clear growth defects across all cell lines (A), for OR5L2 a non-essential gene, where KO does not result in clear growth defects across all cell lines (C), for canonical oncogenic dependencies such as ERBB2 (D), and KRAS (B), and synthetic lethal associations for MFRN2 (E), where genetic perturbation results in clear growth defects in subsets of cancer cell lines.

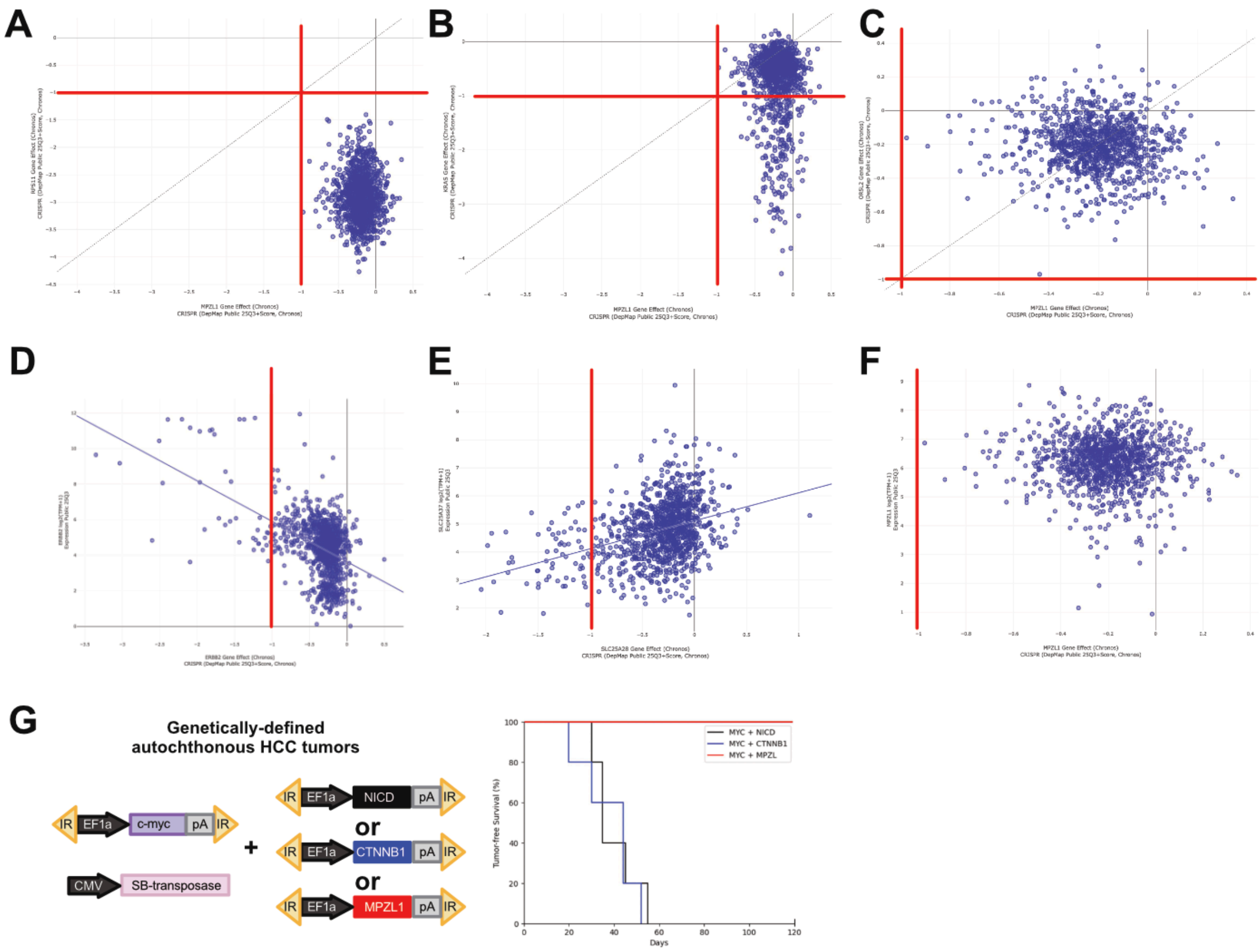

<u>Suppl. Figure S2.</u> MPZL1 is not a classical cancer driver.

(A) – (F) DepMap data analyses. Chronos score < -1 (red lines) indicates strong impairment of cell fitness across ~1000 cancer cell lines tested by means of CRISPR-KO screens (dots). **(A)** Chronos scores for MPZL1 KO (x-axis) versus RPS11 (core essential gene) KO (y-axis) **(B)** Chronos scores for MPZL1 KO (x-axis) versus KRAS (well-established cancer driver in the subset of mutant KRAS cells) KO (y-axis). **(C)** Chronos scores for MPZL1 KO (x-axis) versus OR5L2 (olfactory receptor; core non-essential gene) KO (y-axis). **(D)** Chronos scores for ERBB2 KO (x-axis) versus ERBB2 mRNA expression (y-axis).**(E)** Chronos scores for SLC25A28/MFRN2 KO (x-axis) versus SLC25A37/MFRN1 mRNA expression (y-axis). **(F)** Chronos scores for MPZL1 KO (x-axis) versus MPZL1 mRNA expression (y-axis). (**G) HTVI liver cancer mouse models tested**. MYC + MPZL1 did not results in tumor development. (n=5 animals).

Second, we provide additional in vivo evidence from our hydrodynamic tail vein injection liver cancer model. By testing MPZL1 overexpression in comparison to two known HCC-cancer drivers we show that, while expression of MYC together with either activated NOTCH1 (NICD) or mutant CTNNB1 results in rapid tumor development, we did not observe tumor formation upon co-expression of MYC and MPZL1 (Supple. Figure S2E). Furthermore, we now better highlight that MPZL1 overexpression did not alter

tumorigenesis in the MYC + p53 knockout context (as already indicated in the original manuscript), nor did it induce lung metastasis in this model. We now state:

"…we first generated a cohort of MycOE;p53-/-;GFP mice and MycOE;p53-/-;MPZL1 mice without CAR-T cell treatment and found that tumor onset and survival of both groups did not show significant differences… Further, detailed necropsy revealed no pulmonary metastases in both the MycOE;p53-/-;MPZL1 and MycOE;p53-/-;GFP cohorts, arguing against a critical role for MPZL1 in metastatic progression in this genotypically defined mouse model (data not shown)."

Taken together, these results suggest that MPZL1 does not fulfill the criteria of a classical cancer driver gene required for tumor initiation or maintenance under the experimental conditions tested.

Importantly, we fully acknowledge prior studies reporting oncogenic functions of MPZL1, including roles in migration and invasion. In line with these reports, we now explicitly discuss that MPZL1 whose perturbation does not affect cell proliferation may still contribute to other cancer-relevant phenotypes, such as metastatic behavior. We have revised the manuscript accordingly to better reflect this distinction.

"While additional work is required to definitively establish a causal relationship between MPZL1 expression and metastatic potential, as has previously been suggested [33], [36], [37], MPZL1-directed therapy may accordingly target tumor cells with elevated metastatic capacity lending further support for focusing on this cell surface receptor. "

And

"Yet, even if we did not observe associations between elevated MPZL1 expression and metastasis potential in the autochthonous liver cancer mouse model tested in our study, MPZL1 has been proposed to promote migratory and invasive capabilities [33], [36], [37]. Therefore, MPZL1-directed therapy may preferentially target tumor cells with enhanced metastatic capacity, potentially providing an added therapeutic benefit."

2, The authors conclude that MPZL1 is a non-essential gene and therefore suggest that MPZL1-ChAb is not expected to function as a therapeutic antibody. This conclusion requires much stronger evidence. For example, the authors should provide experimental data showing whether MPZL1-ChAb alone has anti-tumor effects in HCC or other MPZL1-high xenograft models. Reconcile their conclusion with the fact that MPZL1 has been reported to exert oncogenic functions in several cancers, including HCC and NSCLC.

Based on the experimental evidence outlined above, MPZL1 does not appear to function as a classical growth-driving oncogene whose perturbation directly alters tumor cell proliferation. Consistent with this notion, treatment of several cancer cell lines with our newly developed MPZL1-ChAb did not measurably affect cell growth in vitro (Suppl. Figure S4). These observations are in line with functional genomics data indicating that loss of MPZL1 does not impair proliferation across a broad panel of cancer cell lines in the Cancer Dependency Map (Suppl. Figure S2).

Based on these results, we reasoned that therapeutic strategies relying on direct inhibition of MPZL1 were unlikely to produce strong anti-tumor effects. Instead, we focused on exploiting the elevated surface expression of MPZL1 as a targeting handle for immune-based therapies and therefore prioritized the development of MPZL1-directed CAR-T cells. Indeed, our results demonstrate that MPZL1 CAR-T cells efficiently eradicate MPZL1-expressing tumors in vivo (Figure 5).

We fully acknowledge that we cannot completely exclude the possibility that MPZL1-ChAb could exert anti-tumor activity in specific tumor contexts or model systems. However, given the lack of detectable effects on tumor cell growth in our initial experiments, we did not pursue extensive in vivo evaluation of MPZL1-ChAb as a standalone therapeutic antibody in the context of our current manuscript.

3, Because MPZL1 is expressed in some normal tissues, albeit at lower levels, the authors need to evaluate the potential toxicities of MPZL1-targeting CAR-T cells. Assessment of toxicity in relevant preclinical models is critical. For the autochthonous HCC studies induced by HTVI, the authors use NSG mice, which lack an intact immune system. Given that CAR-T cell function and toxicity are highly influenced by the host immune environment, the authors should use immunocompetent C57BL/6 mice for the HTVI-based HCC model.

We agree that evaluation of MPZL1-directed CAR T-cell therapy in immunocompetent models using murine-specific reagents would further strengthen the translational assessment of our approach. However, as our current CAR construct specifically recognizes human MPZL1 and does not cross-react with the murine protein, such experiments would require the development of surrogate antibodies and corresponding murine CAR-T cells, which until now was unfortunately not successful, despite our continues attempts to achieve this goal.

We would therefore like to highlight that we have now tried to address this limitation by leveraging a preclinical model closely reflecting human biology, in that we utilized a fully human T cell–tracing tissue explant system, including both MPZL1-positive tumor samples from different entities and patient-matched healthy tissue samples (new Fig. 6A, Suppl. Fig. S8). To this end, freshly resected explant sections were treated with CMFDA-labeled T cells (CLT) and cultivated overnight following examination of infiltration across different human patient samples (Figure 6A, see Methods). Further, T cell activity was assessed via granzyme B (GranB) immunofluorescence staining (Suppl. Figure S8C) and multiplex cytokine analysis. We observed that MPZL1 CAR-T cells displayed markedly higher infiltration into MPZL1 positive tumor explants compared with naïve and activated UT T cells, as well as CAR-T cells targeting carcinoembryonic antigen (CEA), ovarian cancer antigen 125 (CA125) or CD19 that we tested to additionally confirm MPZL1 CAR T-cell specificity (Figure 6B, C). Positive tissue expression of MPZL1, CA125 and CEA in corresponding patient tissues was confirmed by immunohistochemistry (Suppl. Figure S8A, B). Of note, the high infiltration of MPZL1 CAR-T cells was accompanied by significantly elevated levels of effector molecules, including granzyme B (GranB), CXCL9 (MIG), CXCL10 (IP10), interferon gamma (IFNγ), tumor necrosis factor alpha (TNFα), and interleukin 2 (IL-2), relative to other CAR-T or

UT T cells (Figure 6D, E). Importantly, comparison with patient-matched healthy tissue explants revealed lower MPZL1 CAR-T cell infiltration and significantly reduced effector molecule induction in normal tissues (Figure 6E-G). Together, these initial human tissue sample-based results indicate selective activity of MPZL1 CAR-T cells toward tumor cells as opposed to healthy patient-matched human tissue.

Despite this encouraging new data from tissue explant assays, we have again highlighted the limitations of our current approaches:

"While our studies do not fully address systemic on-target/off-tumor toxicities—given the lack of cross-reactivity with murine MPZL1, the use of immunocompromised mouse models, and the limited availability of healthy patient-matched tissue samples from multiple organs suitable for tissue explant assays—existing examples from other tumor-associated antigens (e.g., HER2, CLDN18.2) suggest that limited expression in normal tissues as opposed to markedly elevated tumor-specific expression can be compatible with tolerable safety profiles for surface protein targeting therapeutic approaches."

Reviewer #3 (Remarks to the Author):

Summary

This manuscript presents an ambitious end-to-end study that starts from a pan-cancer multiomics screen for amplified cell surface proteins and culminates in the preclinical development of MPZL1-directed CAR T-cells. The authors propose that passenger somatic copy number amplifications affecting genes that encode cell surface proteins can be exploited therapeutically as "Trojan horse" targets, and they use MPZL1, a glycosylated cell surface receptor on chromosome 1q, as a worked example. MPZL1 is reported to be amplified in up to roughly seventy-five percent of cases in several solid cancers, with immunohistochemistry suggesting tumour restricted membrane localisation. The authors generate a monoclonal antibody against MPZL1, engineer MPZL1 directed CAR T cells, and demonstrate efficacy in vitro and in xenograft models.

Overall, this is a broad and technically impressive piece of work with clear translational intent. The end-to-end nature of the pipeline, spanning in silico target discovery, antibody and CAR generation, functional validation, and in vivo efficacy, makes the study feel self-contained and suitable for a high-impact translational journal. At the same time, the scope sometimes makes the manuscript feel like three papers in one. As a reader, I did feel some fatigue simply due to the volume of data. In my view, the main remaining issues relate to the narrative focus and conceptual framing around passenger amplifications and Trojan horse targets, and to translational nuances around MPZL1 expression in normal tissues, antigen density, and target selection.

We thank the reviewer for their positive assessment and for recognizing the breadth and translational significance of our work. We greatly appreciate the constructive feedback and encouragement, which helped us refine the presentation of our study.
We have extensively edited out manuscript to avoid overtly detailed descriptions of our efforts to validate antibodies and CAR-T cell properties in order to provide a better narrative focus and conceptual framing.

Major comments

1. The manuscript is very comprehensive, which is a strength, but it can at times feel like three papers in one. It reads as a target discovery study, an antibody and CAR engineering paper, and a translational in vivo efficacy paper, rather than a single tightly focused narrative. Important aspects of the work, particularly limitations, edge cases, and less tidy results, risk being buried within the volume of data. This may make it harder for readers outside the immediate subfield to identify and retain the main message. Some of the more technical or confirmatory experiments could be moved to the supplementary information without reducing the overall impact.

We have extensively edited our manuscript to provide a better narrative focus and conceptual framing.
We have moved more technical or confirmatory experiments and the respective description to the supplement (e.g. Figure 4E-H).

2. As a concept, the Trojan horse metaphor is somewhat confusing in this setting. Traditionally a Trojan horse is something dangerous hidden inside an apparently benign

structure that is deliberately brought inside. MPZL1 is an endogenous protein that the tumour naturally expresses. You are exploiting it as a vulnerability rather than smuggling in a deceptive payload. If one wants to stay with Greek metaphors, MPZL1 seems more analogous to an Achilles heel than a Trojan horse. This may be a matter of taste, but the current framing did make me pause. It may help to sharpen and standardise the language around passenger amplification and therapeutic entry point rather than relying heavily on the Trojan horse image.

We have edited our manuscript to avoid the Trojan horse metaphor and have clarified our definition of cancer-driving genes (see below). Accordingly, we have changed the title of our manuscript.

3. Early in the manuscript the authors identify frequently amplified genes on chromosomal arms 1q and 8q and focus on 1q as a hotspot for amplified cell surface receptors. The authors then narrow down nineteen differentially expressed 1q cell surface protein genes and select those annotated as plasma membrane proteins, from which MPZL1 is chosen as a potentially targetable bystander amplification. (A) Did you systematically compare the expression profiles and tractability of other 1q cell surface proteins such as ATP1B1, EFNA4, IGSF8, ADAM15, and others? Given that you argue that the exact role of MPZL1 in cancer biology is not essential for its use as a targeting moiety, what disqualified other candidates?

In our study, we focused on MPZL1 as an illustrative example for our conceptual framework. We did not systematically evaluate all other 1q-amplified cell surface proteins for CAR-T tractability, as the goal was to demonstrate proof-of-concept that amplified cell surface genes can serve as tumor-selective targets independent of their role as classical oncogenic drivers.
While other candidates such as ATP1B1, EFNA4, IGSF8, and ADAM15 may also represent promising targets, their evaluation is beyond the scope of this work and could be addressed in future studies building on the pipeline we describe.

(B) Related to this, could MPZL1 be more than a pure passenger? The evidence presented in this section appears largely correlative, based on copy number frequency and expression. Have you examined whether MPZL1 copy number or expression associates with survival? Perhaps a clearer definition of "driver" versus "passenger" needs to be articulated.

In the revised manuscript, we clarified our definition of genes with canonical cancer-driving functions. Specifically, we consider a gene a classical driver if perturbation directly alters tumor cell fitness, most commonly assessed through effects on proliferation or survival in large-scale loss-of-function screens or experimental cancer models.

To avoid potential confusion, we no longer refer to MPZL1 amplification as a "passenger" event. Instead, we emphasize that available experimental evidence indicates that MPZL1 does not function as a classical growth-driving oncogene.

First, we include data from the Cancer Dependency Map showing that knockout of MPZL1 does not impair proliferation across a broad panel of over 1000 cancer cell lines. In contrast, KO of canonical cancer drivers such as KRAS and ERBB2 show clear growth defects in subsets of cell lines (Supple. Figure S2).

Second, in our hydrodynamic tail vein injection (HTVI) liver cancer model, expression of MYC together with either activated NOTCH1 or mutant CTNNB1 induces rapid tumor development, whereas co-expression of MYC and MPZL1 did not result in tumor formation (Supple. Figure S2E). Similarly, MPZL1 overexpression in the MYC + p53 knockout context did not alter tumorigenesis or induce lung metastasis.

Collectively, these results suggest that MPZL1 does not fulfill the criteria of a classical cancer driver required for tumor initiation or maintenance under the conditions tested.

We note that analyzing associations between MPZL1 copy number or expression and patient survival would likewise be largely correlative and not based on direct experimental evidence. Importantly, we acknowledge prior studies reporting possible oncogenic functions of MPZL1 in migration and invasion. Accordingly, we now discuss that genes like MPZL1, whose perturbation does not affect proliferation, may still contribute to other cancer-relevant phenotypes, such as metastatic behavior, and have revised the manuscript to highlight this distinction.

“While additional work is required to definitively establish a causal relationship between MPZL1 expression and metastatic potential, as has previously been suggested [33], [36], [37], MPZL1-directed therapy may accordingly target tumor cells with elevated metastatic capacity lending further support for focusing on this cell surface receptor. “

and

“Yet, even if we did not observe associations between elevated MPZL1 expression and metastasis potential in the autochthonous liver cancer mouse model tested in our study, MPZL1 has been proposed to promote migratory and invasive capabilities [33], [36], [37]. Therefore, MPZL1-directed therapy may preferentially target tumor cells with enhanced metastatic capacity, potentially providing an added therapeutic benefit.”

4. The authors note that the pipeline relies on a single open access resource for membrane bound proteins. Why restrict yourself to one resource at the outset? It would be reassuring to know that the analysis has been cross validated against, or could readily incorporate, additional databases such as the Human Protein Atlas, UniProtKB, MemProtMD, UniTmp, or AmiGO 2. Even if you prefer to keep the current pipeline unchanged for this manuscript, a short discussion of how expanding to multiple resources might affect target discovery, and whether MPZL1 would still have been prioritised, would clarify the robustness of your approach.

We agree with the reviewer that integrating multiple independent resources can increase the robustness of target identification. In the revised manuscript, we now explicitly note that, in addition to the mass spectrometry–derived Cell Surface Protein Atlas that

provided our initial resource, we consulted publicly available databases, including UniProt and the Human Protein Atlas, to confirm that MPZL1 is surface-exposed, complementing our own IHC-based validation.

Specifically, we now state:

“This analysis identified 90 cell surface protein (CSP)-coding genes (Suppl. Table S1), as amplified in up to 30% of tumors, with a strong clustering on chromosome 1q (n = 77), 20 of which were likewise identified to localize to the plasma membrane in the Human Protein Atlas.”

To provide additional context, a direct comparison between the Human Protein Atlas (2,452 cell surface protein genes) and the Cell Surface Protein Atlas (1,492 genes) reveals an overlap of 401 genes. Our 90 candidate genes within amplified regions are a subset of this overlapping set of which 20 were observed in both databases.

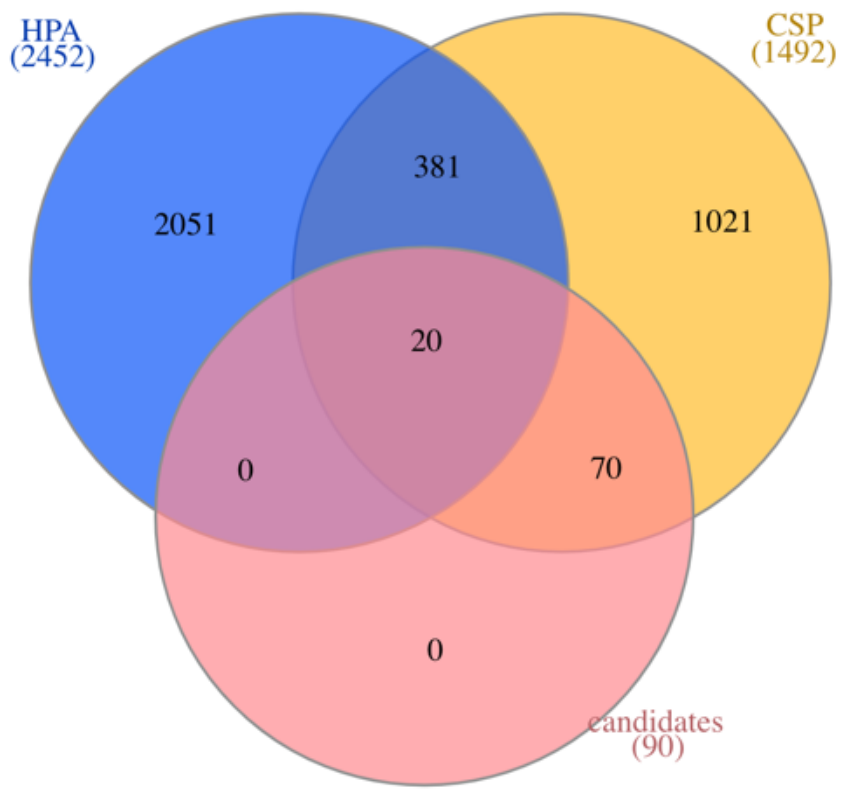

HPA (Human Protein Atlas): 2452 genes classified as localized to the cell membrane.
CSP (spectrometry–derived Cell Surface Protein Atlas) 1492 genes classified as localized to the cell membrane.

Overlap between HPA and CSP: 401 genes.

20 of the 90 candidate genes identified within amplified regions intersect with both HPA and CSP.

Further, we state that

“Integrating differential expression analysis (GEPIA2, [27]) with additional protein localization data (**UniProt**) [28], [29] revealed that 19 chr1q CSP genes (Suppl. Table S2) were ≥2-fold upregulated in tumors versus normal tissues across these entities, of which **six localize preferentially to the plasma membrane…”**

i.e UniProt data indicated membrane localization for only 6 of these 19 genes for which membrane associations were predicted based on HPA and CSP.

This simple comparison highlights substantial differences between publicly available databases, underscoring that experimental characterization—as performed in our study—is essential to resolve inconsistencies or ambiguities and to provide confidence in target identification (see below).

5. The authors use GEPIA2 to compare expression of the cell surface protein genes in tumour versus matched normal tissues, for example liver cancer versus normal liver or breast cancer versus normal breast. However, restricting analysis to counterpart tissues

misses expression in other critical organs. For example, data from the Human Protein Atlas indicate rather ubiquitous MPZL1 expression across normal tissues. (A) It would strengthen the translational argument to address this more directly, perhaps by showing broader normal tissue expression profiles at the RNA level.

To strengthen the translational argument while not solely relying on correlative expression analysis and instead obtaining direct experimental evidence, we have now included data from a preclinical model closely reflecting human biology, in that we utilized a fully human T cell–tracing tissue explant system, including both MPZL1-positive tumor samples from different entities and patient-matched healthy tissue samples.
To this end, freshly resected explant sections were treated with CMFDA-labeled T cells (CLT) and cultivated overnight following examination of infiltration across different human patient samples (Figure 6A, see Methods). Further, T cell activity was assessed via granzyme B (GranB) immunofluorescence staining (Suppl. Figure S8C) and multiplex cytokine analysis. We observed that MPZL1 CAR-T cells displayed markedly higher infiltration into MPZL1 positive tumor explants compared with naïve and activated UT T cells, as well as CAR-T cells targeting carcinoembryonic antigen (CEA), ovarian cancer antigen 125 (CA125) or CD19 that we tested to additionally confirm MPZL1 CAR T-cell specificity (Figure 6B, C). Positive tissue expression of MPZL1, CA125 and CEA in corresponding patient tissues was confirmed by immunohistochemistry (Suppl. Figure S8A, B). Of note, the high infiltration of MPZL1 CAR-T cells was accompanied by significantly elevated levels of effector molecules, including granzyme B (GranB), CXCL9 (MIG), CXCL10 (IP10), interferon gamma (IFNγ), tumor necrosis factor alpha (TNFα), and interleukin 2 (IL-2), relative to other CAR-T or UT T cells (Figure 6D, E). Importantly, comparison with patient-matched healthy tissue explants revealed lower MPZL1 CAR-T cell infiltration and significantly reduced effector molecule induction in normal tissues (Figure 6E-G). Together, these initial human tissue sample-based results indicate selective activity of MPZL1 CAR-T cells toward tumor cells as opposed to healthy patient-matched human tissue.

We believe that direct experimental evidence more convincingly strengthens the translational argument. Yet, despite this encouraging new data from tissue explant assays, we are well aware of the limitations and therefore state:

"While our studies do not fully address systemic on-target/off-tumor toxicities—given the lack of cross-reactivity with murine MPZL1, the use of immunocompromised mouse models, and the limited availability of healthy patient-matched tissue samples from multiple organs suitable for tissue explant assays—existing examples from other tumor-associated antigens suggest that limited expression in normal tissues as opposed to markedly elevated tumor-specific expression can be compatible with tolerable safety profiles for surface protein targeting therapeutic approaches."

Later in the manuscript, you present your own immunohistochemistry across a panel of normal tissues. (B) It would be helpful to reconcile these internal findings with the public databases. To what extent are they concordant or discordant, and how do you interpret any discrepancies?

We have now included a statement to better describe our efforts to analyze MPZL1 protein expression in a panel of normal tissues and a large number of tumor samples using a commercially available MPZL1 antibody distinct from that used in the Human Protein Atlas (HPA) datasets. We highlight, that immunohistochemistry was performed using a scoring strategy that emphasizes membrane localization. By doing so, we provide a complementary perspective on MPZL1 expression, leveraging a different antibody whose specificity for human MPZL1 we have validated using a panel of MPZL1 knockout cell lines.

“Next, we queried the Human Protein Atlas (HPA), which reports immunohistochemistry (IHC) data from tissue microarrays of 144 normal individuals across 44 tissue types and 216 cancer patients across 20 cancer types which indicated predominantly cytoplasmic MPZL1 expression with a granular pattern in several tissues and suggested that high MPZL1 expression is associated with unfavorable prognosis in several tumor types (e.g., HCC; https://www.proteinatlas.org/ENSG00000197965-MPZL1). To provide a complementary perspective on MPZL1 protein expression, we directly assessed MPZL1 expression in human tissues by performing IHC on paraffin-embedded samples using a commercial antibody whose specificity we validated in MPZL1 knockout as well as forced MPZL1 overexpression cell lines (Suppl. Fig. S3). Our analyses encompassed 2,244 resected cancers across six tumor entities and 30 normal tissues (three independent samples per tissue; Table 3) (Figure 2C-F). As we observed strong cell surface MPZL1 expression in multiple tumor samples, we developed a scoring system for MPZL1 levels at the plasma membrane (Figure 2D), analogous to the well-established HER2 scoring system used in breast cancer [39].

In Figure 2D, where you compare normal and tumour tissue, the immunohistochemistry pattern is intriguing. (C) Are you proposing that MPZL1 is largely intracellular in normal physiology but becomes more prominently membrane bound in cancer? If so, can you speculate on a mechanism, for example altered trafficking, post translational modifications, or interactions with other partners? If not, clarifying what you think the immunohistochemistry is showing would help.

As shown in Figures 2C–E, MPZL1 membrane expression is increased in malignant tissues compared to normal tissues, where expression is generally weaker and predominantly intracellular. While our data indicate that MPZL1 undergoes glycosylation (Suppl. Figure S3), one possible explanation is altered trafficking in tumor cells, potentially leading to enhanced retention at the plasma membrane via endosomal recycling pathways. We attempted to test this mechanism in cancer cell lines using Brefeldin A to block ER → Golgi → membrane trafficking and performing FACS analysis with our new antibody, but these experiments were inconclusive. Given the lack of direct experimental evidence, we refrain from directly proposing mechanistic models to explain the increased surface expression in tumors we observed in our IHC analyses.

Yet, in the discussion section, we also note that our newly developed MPZL1-ChAb does not recognize murine MPZL1 and is not functional in IHC or Western blot but highly specific in FACS. While speculative, similar considerations may apply to the

commercially available antibody used for our IHC analyses as well as the antibody used to detect MPZL1 in the HPA dataset. This could reflect recognition of conformational or discontinuous epitopes or sensitivity to tumor-specific glycopatterns as mentioned in the discussion section.

6. The authors score normal tissues as immunohistochemistry zero to two, but CAR T cells are known to be exquisitely sensitive to antigen density and can attack normal cells that express even low levels of target antigen, particularly in critical organs. (A) Could you comment more explicitly on how your immunohistochemistry scoring, and any quantitative estimates of antigen density if available, map onto likely CAR T cell recognition thresholds?

We thank the reviewer for highlighting the potential sensitivity of CAR-T cells to even low levels of antigen expression in normal tissues. Based on our data, MPZL1 is expressed at substantially higher levels on the plasma membrane of tumor cells compared with healthy tissues, suggesting the existence of a potential therapeutic window.

It is important to note that IHC is a semi-quantitative method that provides spatial information on protein expression but does not yield absolute measures of antigen density (e.g., molecules per cell) and is therefore insufficient to predict CAR-T cell recognition thresholds. While our IHC data indicate markedly lower MPZL1 expression in normal tissues, we fully acknowledge that even low antigen expression could trigger CAR-T cell activity, raising safety concerns, particularly in critical organs. Addressing these safety considerations would be essential prior to any clinical application and represents an important area for future preclinical studies.

As outlined above we have now included data from a preclinical model closely reflecting human biology, in that we utilized a fully human T cell–tracing tissue explant system, including both MPZL1-positive tumor samples from different entities and patient-matched healthy tissue samples. These analysis indicate selective activity of MPZL1 CAR-T cells toward tumor cells as opposed to healthy patient-matched human tissue (Figure 6). Yet, the limited number of samples does not allow for the definition of thresholds related to our scoring system indicative of CAR-T cell activity.

B) More broadly, the functional data you present are largely in MPZL1 high cell lines and models. We see convincing efficacy in high antigen contexts, with multiple entities, isogenic pairs, effector to target titrations, cytokine and exhaustion phenotyping, but less about intermediate or low antigen density. This is clinically very relevant given the heterogeneity of MPZL1 expression across tumours.

We thank the reviewer for highlighting this point. Indeed, our functional studies were largely performed in models with discernable MPZL1 expression, reflecting the majority of tumors with substantial MPZL1 expression, as well as corresponding isogenic knockout cell lines that were considerably less sensitive to CAR-T–mediated killing.
These results underscore the importance of patient stratification based on strong MPZL1 expression and suggest that tumors with intermediate or low MPZL1 levels may indeed exhibit reduced responsiveness.

7. You report that patient 8843 has a high MPZL1 immunohistochemistry score but does not show strong binding of MPZL1 antibody. Could you expand on your interpretation of this discrepancy? Does it suggest epitope variability, differences in glycosylation, or other post translational or structural heterogeneity across tumours? This seems highly relevant for both antibody and CAR design. This point also intersects with the concern about antigen density and heterogeneity. The presence of immunohistochemistry positive yet weakly binding samples might have implications for on target efficacy and potential antigen escape.

We appreciate the reviewer's comment and agree that this discrepancy is potentially relevant. However, given the lack of experimental evidence, proposing specific mechanistic explanations such as epitope variability, differences in glycosylation, etc. is highly speculative.

We would rather like to highlight that the experimental framework used to compare antibody binding with immunohistochemical MPZL1 staining is technically challenging. For this analysis, we obtained matched samples consisting of cryopreserved dissociated tumor cells (DTCs) and corresponding FFPE tumor tissue blocks from five breast cancer patients undergoing tumor resection.

Importantly, dissociated tumor cells represent a heterogeneous population derived from the tumor tissue, and the number of viable cells available for analysis was limited. Under these technically constrained conditions, we observed concordant results between MPZL1 immunohistochemistry and antibody binding in four of the five patient samples analyzed. We therefore interpret the observed discrepancy in patient 8843 with caution and consider it within the expected variability of this technically demanding comparison.

Nevertheless, the concordance observed in the majority of samples indicates that MPZL1 detected by immunohistochemistry generally corresponds to antibody-accessible surface MPZL1, suggesting that the epitope recognized by our antibody is present and accessible in most tumors analyzed.

8. Figure 2 contains extensive experimental work characterising MPZL1 as a cell surface protein. While this is impressive, much of this information might already be inferred from existing online resources. The purpose of such databases is partly to remove the need for exhaustive re validation. It would be helpful to state explicitly whether this experimental characterisation was performed to resolve inconsistencies or ambiguities in the databases, to establish best practice in the context of CAR T development, or for another reason. Making this motivation explicit in the narrative could help the reader understand why this level of experimental detail is included in the main figures. (see point #1 – could this be supplement?)

As indicated above (point 4), substantial differences between publicly available databases can exist, and as such we strongly encourage direct experimental characterization.

We have now included a statement related to our effort to analyze MPZL1 protein expression in our own collection of samples and a commercially available MPZL1 by means of IHC. We highlight, that immunohistochemistry was performed using a scoring strategy that emphasizes membrane localization. By doing so, we provide a complementary perspective on MPZL1 expression, leveraging a different antibody whose specificity for human MPZL1 we have validated using a panel of MPZL1 knockout cell lines.

“Next, we queried the Human Protein Atlas (HPA), which reports immunohistochemistry (IHC) data from tissue microarrays of 144 normal individuals across 44 tissue types and 216 cancer patients across 20 cancer types which indicated predominantly cytoplasmic MPZL1 expression with a granular pattern in several tissues and suggested that high MPZL1 expression is associated with unfavorable prognosis in several tumor types (e.g., HCC; https://www.proteinatlas.org/ENSG00000197965-MPZL1). To provide a complementary perspective on MPZL1 protein expression, we directly assessed MPZL1 expression in human tissues by performing IHC on paraffin-embedded samples using a commercial antibody whose specificity we validated in MPZL1 knockout as well as forced MPZL1 overexpression cell lines (Suppl. Fig. S3). Our analyses encompassed 2,244 resected cancers across six tumor entities and 30 normal tissues (three independent samples per tissue; Table 3) (Figure 2C-F). As we observed strong cell surface MPZL1 expression in multiple tumor samples, we developed a scoring system for MPZL1 levels at the plasma membrane (Figure 2D), analogous to the well-established HER2 scoring system used in breast cancer [39].”

Minor comments

1. Notation and terminology
In running text, chromosome 1q reads more smoothly than chr. 1q. If a short form is preferred, chr1q is cleaner. Please use Mb for megabases throughout. (example: p 4 line 146)

We have adjusted our manuscript text and the figures accordingly.

The phrase describing MPZL1 antibody as a carrier for a therapeutic agent feels slightly imprecise in the CAR T context, where the antibody derived single chain fragment primarily serves as the targeting moiety rather than a classical carrier. Consider rephrasing for clarity, for example that it serves as a targeting domain for therapeutic effectors. (page 7 line 261-262)

The edited version of our manuscript avoids this phrasing.

2. Grammar check: Page 8 line 282 & Page 9 line 300. “new generated” is ungrammatical & comma after “immunohistochemistry”.

We have adjusted our manuscript accordingly.

3. At one point (p5 line 173/174) you refer to the patient cohort as unique. As currently phrased, this could be read as implying that the tumours themselves are biologically unusual. I think what you mean, and what is indeed impressive, is that you have multiple modalities on the same tissue, for example copy number, expression, immunohistochemistry and so on, which is technically non-trivial and valuable. I suggest rephrasing to emphasise that the dataset, namely multi modal profiling on matched samples, is unique, rather than the patients tumours themselves.

We have adjusted our manuscript accordingly.

Reviewer #4 (Remarks to the Author):

This manuscript proposes MPZL1 as a potential target for CAR T-cell therapy.

Major Concerns

The authors claim that MPZL1 is overexpressed in tumor tissues but minimally expressed in normal tissues based on immunohistochemistry (IHC) data generated using a rabbit monoclonal antibody (clone D17B10). However, it is unclear whether this antibody is truly specific to MPZL1 and does not cross-react with other human proteins. This specificity should ideally be validated using platforms such as Retrogenix Cell Microarray or Origene High-Density Protein Microarray. That said, performing such costly specificity assays with an antibody that is not intended for future CAR development may be of limited practical value. Consequently, many groups instead generate their own antibodies that are compatible with both IHC and CAR construction and then perform comprehensive specificity screening using Retrogenix or equivalent platforms. For clinical CAR T-cell development, regulatory agencies strongly recommend—or effectively require—that the antibody used for CAR generation undergo rigorous specificity validation. In the absence of such data, it is premature and undesirable to conclude that MPZL1 is an appropriate target for CAR T-cell therapy.

Optional Future Direction

As MPZL1 represents a novel target for CAR T-cell therapy, future studies could consider generating a mouse surrogate antibody, developing mouse CAR T cells targeting murine MPZL1, and evaluating antitumor efficacy and on-target/off-tumor toxicity in an immunocompetent mouse model. Such work would further strengthen the translational relevance of MPZL1-directed CAR T-cell therapy but is not essential for the conclusions of the current manuscript. The authors might include this point in the discussion.

For the MPZL1 rabbit monoclonal antibody (clone D17B10), as well as our newly developed MPZL1-ChAb, we confirm specificity using a panel of MPZL1 knockout and overexpression cell lines. Although platforms such as the Retrogenix Cell Microarray or the Origene High-Density Protein Microarray can provide orthogonal validation, knockout cells represent a cost-effective, robust and physiologically relevant model for assessing antibody specificity.

That being said, we agree that evaluation of MPZL1-directed CAR-T cell therapy in immunocompetent models using murine-specific reagents would further strengthen the translational assessment of this approach. However, as our current CAR construct specifically recognizes human MPZL1 and does not cross-react with the murine protein, such experiments would require the development of surrogate antibodies and corresponding murine CAR-T cells, which until now was not successful.

To avoid premature conclusions regarding clinical translation, we have revised the manuscript accordingly. Our aim is not to suggest that MPZL1-directed CAR-T cell therapy is immediately ready for clinical testing, but rather to demonstrate a general strategy for identifying and exploiting amplified cell surface proteins as therapeutic entry

points. Specifically, our study describes the identification of MPZL1 as a candidate target, the generation of an antibody recognizing its extracellular domain, the development of MPZL1-specific CAR-T cells, and the demonstration that these CAR-T cells efficiently eradicate MPZL1-expressing tumors in multiple experimental systems. In addition, we have performed additional experiments using human tissue explant assays that reveal increased tumor-specific activity of MPZL1 CAR-T cells compared with normal tissues.

Collectively, these findings provide a proof-of-concept framework for identifying and functionally validating new therapeutic targets independent of prior knowledge of their involvement in canonical cancer hallmarks.

We explicitly state:
"… our studies do not fully address systemic on-target/off-tumor toxicities—given the lack of cross-reactivity with murine MPZL1, the use of immunocompromised mouse models, and the limited availability of healthy patient-matched tissue samples from multiple organs suitable for tissue explant assays.."

and aim to focus on the proof-of-concept aspect of our approach by stating:

"Although additional studies will be required to fully address potential safety considerations, the successful development of MPZL1 CAR-T cell that eradicate MPZL1 positive tumors cells described here, demonstrates the feasibility of this strategy and provides a blueprint for targeting amplified cell surface proteins in human cancers."

Minor Concerns

1) It can be controversial to say MPZL1 the true 'passenger'

The authors show that MPZL1 overexpression or knockout does not alter cell growth or colony formation in cell lines, supporting the hypothesis that MPZL1 behaves as a non-essential bystander in tumorigenesis. However, there are previous reports showing that MPZL1 promotes tumor cell proliferation, migration and invasion [Oncology reports, 2019, 42.2: 679-687. Cell research, 2014, 24.2: 204-217. Genes & Diseases, 2024, 11.5: 101085. Carcinogenesis, 2022, 43.10: 919-929. Human Gene Therapy, 2023, 34.11-12: 540-553.]. The authors might include this point in the discussion.

In the revised manuscript, we clarified our definition of genes with canonical cancer-driving functions. Specifically, we consider a gene a classical driver if perturbation directly alters tumor cell fitness, most commonly assessed through effects on proliferation or survival in large-scale loss-of-function screens or experimental cancer models.

To avoid potential confusion, we no longer refer to MPZL1 amplification as a "passenger" event. Instead, we emphasize that available experimental evidence indicates that MPZL1 does not function as a classical growth-driving oncogene.

First, we include data from the Cancer Dependency Map showing that knockout of MPZL1 does not markedly impair proliferation across a broad panel of over 1000 cancer cell lines. In contrast, canonical oncogenic dependencies such as KRAS and ERBB2 show clear growth defects in subsets of cell lines upon perturbation (Supple. Figure S2).

Second, in our hydrodynamic tail vein injection (HTVI) liver cancer model, expression of MYC together with either activated NOTCH1 or mutant CTNNB1 induces rapid tumor development, whereas co-expression of MYC and MPZL1 did not result in tumor formation (Supple. Figure S2). Similarly, MPZL1 overexpression in the MYC + p53 knockout context did not alter tumorigenesis or induce lung metastasis.

Collectively, these results suggest that MPZL1 does not fulfill the criteria of a classical cancer driver required for tumor initiation or maintenance under the conditions tested.

Importantly, we acknowledge prior studies reporting possible oncogenic functions of MPZL1 in migration and invasion. Accordingly, we now discuss that genes like MPZL1, whose perturbation does not affect proliferation, may still contribute to other cancer-relevant phenotypes, such as metastatic behavior, and have revised the manuscript to highlight this distinction.

“While additional work is required to definitively establish a causal relationship between MPZL1 expression and metastatic potential, as has previously been suggested [33], [36], [37], MPZL1-directed therapy may accordingly target tumor cells with elevated metastatic capacity lending further support for focusing on this cell surface receptor. “

and

“Yet, even if we did not observe associations between elevated MPZL1 expression and metastasis potential in the autochthonous liver cancer mouse model tested in our study, MPZL1 has been proposed to promote migratory and invasive capabilities [33], [36], [37]. Therefore, MPZL1-directed therapy may preferentially target tumor cells with enhanced metastatic capacity, potentially providing an added therapeutic benefit.”

2) Additional methodological detail for monoclonal antibody generation

Describing more experimental methods and results will improve the confidence in the MPZL1-ChAb. Such as: Details of antigen, Number of mice immunized, immunization schedule (number of boosts and intervals), How the titer of immunization was determined and how the ‘strong titers’ threshold was defined.

We have added a more detailed explanation of the experimental methods in the Methods section:

“Generation of an antibody targeting MPZL1
The hybridoma technology was used to generate antibodies against the extracellular domain of MPLZ1 protein. Briefly, 6 mice (3 each from strain Balb/C or C57BL6/N) were immunized with the extracellular fragment of human MPZL1 protein (extracellular domain of human MPZLq protein; aa 36-165: SALEV YTPKEIFVAN GTQGKLTCKF

KSTSTTGGLT SVSWSFQPEG ADTTVSFFHY SQGQVYLGNY PPFKDRISWA GDLDKKDASI NIENMQFIHN GTYICDVKNP PDIVVQPGHI RLYVVEKENL PVFPV in E. coli (pQE-vector system, HIS tag) by 4 subcutaneous injections (day 0 with Freund's Adjuvant complete, day 7 with Freund's Adjuvant incomplete, day 28 and 32 with Quil A, day 33 test sera, day 34 fusion; The immune response was tested by Western blot using cell lysates form overexpressed hMPZL1 fused with a tag. B lymphocytes of the animal showing the strongest immune response in Western blotting were isolated and fused with immortal myeloma cells to generate hybridoma clones capable of producing unique monoclonal antibodies (mAbs), and the clones were evaluated via FACS. The identification of a positive clone was followed by two rounds of subcloning, and the most specific subclone against MPZL1 was selected. Subsequently, the RNA from this hybridoma was sequenced to identify the variable heavy (V_H) and *kappa* light (V_L) chains (Table 4). These sequences were cloned in frame into two independent human backbone vectors coding for the constant heavy (C_H) and *kappa* light (C_L) chains [40], respectively. Finally, PEI-mediated transfection of HEK293 cells with these two plasmids led to the production of MPZL1-ChAb, composed of a human constant domain and a mouse variable region."

3) In the discussion part, this article draws parallels to the case of claudin 18.2 and HER2, where expression is present in normal tissues but still compatible with therapy in carefully selected patients. However, MPZL1 expression is clearly detectable, even though it is often at low levels (Figure 2F). I would suggest slightly tempering strong safety statements and more explicitly emphasizing that safety remains to be established experimentally, such as in cross-reactive preclinical models.

We agree with the reviewer and now explicitly state:
"… our studies do not fully address systemic on-target/off-tumor toxicities—given the lack of cross-reactivity with murine MPZL1, the use of immunocompromised mouse models, and the limited availability of healthy patient-matched tissue samples from multiple organs suitable for tissue explant assays"

as well as

"…additional studies will be required to fully address potential safety considerations, "

4) Clarify differences between MPZL 1.23/1.44 and MPZL 1.24/1.66
It is not entirely clear to the reader what distinguishes MPZL1.23 from 1.44, or MPZL1.24 from 1.66, beyond the VL-VH versus VH-VL orientation.

We have now included the relevant information into the methods section and the legend to Supple. Figure S4 :

"… MPZL1.24 and MPZL1.66 contain one additional amino acid at the C-terminus compared with MPZL1.23 and MPZL1.44, reflecting the inclusion of terminal nucleotides of the MPZL1-ChAb sequence whose coding status could not be unambiguously resolved during sequence verification."

5) Terminology for cytokine measurement

As Brefeldin A +Monensin are used to block secretion of cytokines and retain cytokines inside the cells, it may be more accurate to describe these data as cytokine ‘production’ or ‘generation’ or ‘intracellular accumulation’ rather than ‘release’ or ‘secretion’

We have adjusted the relevant sections and avoid wordings such as ‘release’ or ‘secretion’.

“Brefeldin A and Monensin (protein transport inhibitor cocktail, Invitrogen) was added to the co-cultures to retain the cytokines within the cells.”

6) Figure numbers appear to be swapped. (Line #229, 235)
Figure 2F > Figure 2E , Figure 2G > Figure 2F

We have adjusted the Figure numberings.

Reviewer #5 (Remarks to the Author):

I co-reviewed this manuscript with one of the reviewers who provided the listed reports. This is part of the Nature Communications initiative to facilitate training in peer review and to provide appropriate recognition for Early Career Researchers who co-review manuscripts.

Thank you for co-reviewing.
